# ITM2B Truncation Promotes Migrasome Formation to Accelerate Renal Cell Carcinoma Growth

**DOI:** 10.1002/advs.202511683

**Published:** 2025-11-30

**Authors:** Qi‐tao Chen, Qiao‐ling Huang, Ming‐zhi Han, Xue‐hui Hong, De‐yi Feng, Lu‐ming Yao, Wen‐bin Hong, Yue Chen, Ya‐ying Huang, Hang‐zi Chen, Qiao Wu

**Affiliations:** ^1^ State Key Laboratory of Cellular Stress Biology School of Life Sciences, Xiamen University Xiamen Fujian 361102 China; ^2^ Department of Gastrointestinal Surgery, Zhongshan Hospital of Xiamen University School of Medicine, Xiamen University Xiamen Fujian 361004 China

**Keywords:** caspase‐7, integral membrane protein 2B (ITM2B), migrasomes, renal cell carcinoma (RCC), tetraspanin 4 (TSPAN4)

## Abstract

Integral membrane protein 2B (ITM2B), a transmembrane protein, frequently undergoes cleavage. The physiological functions of ITM2B are primarily studied in the context of neurological disorders, but their roles in cancers are largely overlooked. Here, it is demonstrated that in renal cell carcinoma (RCC) cells, N‐terminal truncation of ITM2B facilitates migrasome swelling through the recruitment of TSPAN4 and promotes migrasome formation. Moreover, ITM2B truncation acts as a carrier, sorting active caspase‐7 into migrasomes for migracytosis. The active caspase‐7‐enriched migrasomes are then taken up by macrophages, leading to caspase‐7‐induced IL‐6 secretion from macrophages, which eventually aggravates RCC growth through a feedback mechanism. Physiologically, hyperuricemia enhances ITM2B cleavage to aggravate RCC growth. Clinically, RCC tissues tend to produce ITM2B truncations compared with corresponding para‐carcinoma tissues. Moreover, compared with the urine from normal volunteers, that from RCC patients contains higher levels of ITM2B truncation‐enriched migrasomes. This study not only highlights novel functions of ITM2B truncation in migrasome formation and active caspase‐7 migracytosis but also elucidates the role of hyperuricemia in RCC progression via regulation of the ITM2B truncation–migrasome axis.

## Graphical abstract

This study identifies truncated ITM2B as a regulator of migrasome formation in renal cell carcinoma (RCC) cells. The ITM2B truncation not only facilitates migrasome formation by recruiting TSPAN4, but also sorts active caspase‐7 into migrasomes. The internalization of active caspase‐7‐enriched migrasomes by macrophages stimulates IL‐6 secretion to promote RCC growth. Pathologically, hyperuricemia promotes ITM2B‐dependent migrasome formation to accelerate RCC progression.

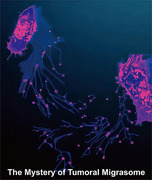

## 1 Introduction

The migrasome, discovered a decade ago, is a vesicular organelle grown up on the retraction fibers (RFs) of migrating cells.^[^ ^1^ ^]^ As cells migrate, RFs are pulled from the trailing edge of cells, and migrasomes are subsequently formed at the ends or intersections of RFs. The tightly regulated process of migrasome formation requires the coordinated actions of various factors in both time and space, including the pairing of integrins with the extracellular matrix (ECM), the assembly of sphingomyelin synthase 2 (SMS2) foci, and the TSPAN4‐enriched macrodomain. The interactions between integrins and ECM proteins provide adhesion for RF tethering.^[^ ^2^, ^3^ ^]^ The assembly of SMS2 foci on the basal membrane at the leading edge of migrating cells promotes sphingomyelin synthesis to predetermine the sites for migrasome formation.^[^ ^4^ ^]^ Eventually, the TSPAN4‐enriched macrodomain is assembled for membrane swelling, leading to migrasome formation.^[^ ^5^ ^]^ Live‐cell imaging and biomimetic systems have revealed two stages in migrasome formation, including a stage initially devoid of TSPAN4 and a stage subsequently enriched with TSPAN4.^[^ ^6^ ^]^ While these studies provide valuable insights into the network involved in migrasome formation, how these processes are regulated to govern migrasome formation under physiological and pathological conditions requires further elucidation.

Migracytosis refers to a process by which cargoes are released from the cell body through migrasomes. Once they undergo migracytosis, cargoes are taken up by recipient cells and perform multiple functions.^[^ ^7^ ^]^ Proteomic analysis of protein cargoes revealed significant differences between migrasomes and the cell body,^[^ ^8^ ^]^ implying the existence of selective sorting mechanisms. However, the precise mechanisms underlying cargo sorting into migrasomes remain largely unknown. The physiological functions of migrasomes have recently been partially elucidated and include coordinating organ morphogenesis during zebrafish gastrulation, promoting embryonic angiogenesis during chicken embryo development, facilitating clot formation at injury sites, and maintaining mitochondrial quality by transporting damaged mitochondria into migrasomes.^[^ ^9^, ^10^, ^11^, ^12^ ^]^ Cancer cells produce migrasomes in vivo, as observed using digital adaptive optical scanning light‒field mutual iterative tomography.^[^ ^13^ ^]^ A recent study demonstrated that tumor‐derived mRNAs and proteins are transferred from tumor cells to osteoclasts via migrasomes, resulting in tumoral bone metastasis; in contrast, blocking migrasome‐mediated communication between tumor cells and osteoclasts by tetracycline‐modified nanoliposomes prevents bone metastasis.^[^ ^14^ ^]^ This study provides evidence of migrasome function and a strategy for targeting cancer cells. However, the functions of migrasomes derived from cancer cells remain to be fully elucidated.

ITM2B is a single‐pass type II transmembrane protein.^[^ ^15^ ^]^ A typical characteristic of ITM2B is its susceptibility to hydrolysis by various proteases, resulting in the generation of distinct truncations with different functions. ITM2B is synthesized as a 266 amino acid precursor and is cleaved by the proprotein convertases (PPCs) ADAM10 and SPPL2a/b at different sites. PPCs, including furin, PACE4, LPC, and PC5/6, cleave ITM2B to generate the ITM2B^1‐243^ and ITM2B^244‐266^ truncations, and Gly60 of ITM2B is essential for its cleavage by SPPL2a/b.^[^ ^15^, ^16^, ^17^ ^]^ However, the precise sites at which ITM2B is cleaved by ADAM10 remain unknown. The physiological functions of ITM2B have long been studied in the context of neurological dysfunction, and diverse truncations of ITM2B play regulatory roles in processing amyloid‐beta precursor protein (APP). In humans, *ITM2B* gene mutations lead to two types of autosomal dominant dementia, namely, familial British dementia and familial Danish dementia, characterized by progressive dementia, cerebellar ataxia, and other clinical symptoms.^[^ ^15^, ^18^, ^19^ ^]^ Although the broad expression of ITM2B and its cleavage in tissues have been demonstrated, its physiological functions, particularly in cancer development, have been ignored and need further investigation.

Renal cell carcinoma (RCC), the most common type of kidney cancer, is highly infiltrated with immune cells, which are closely associated with RCC progression.^[^ ^20^, ^21^, ^22^ ^]^ Although RCC cells have an ability to release migrasomes,^[^ ^14^ ^]^ whether RCC cells influence immune cells via migrasomes remains unreported. In the present study, we elucidated unique functions of ITM2B truncation in RCC cells via the promotion of migrasome formation and sorting active caspase‐7 into migrasomes, which subsequently regulated IL‐6 secretion from macrophages and established a feedback loop to support RCC growth. Physiological hyperuricemia induced ITM2B cleavage, which further accelerated tumor growth in a mouse model. Clinically, RCC patients maintained high levels of ITM2B truncation, and urine from RCC patients had higher levels of migrasomes enriched with ITM2B truncation. As such, this study provides novel insights and a potential target for clinical RCC therapy through the modulation of migrasome.

## 2 Results

### 2.1 Truncated ITM2B Promotes the Growth of Renal Cell Carcinoma

**Figure 1:**
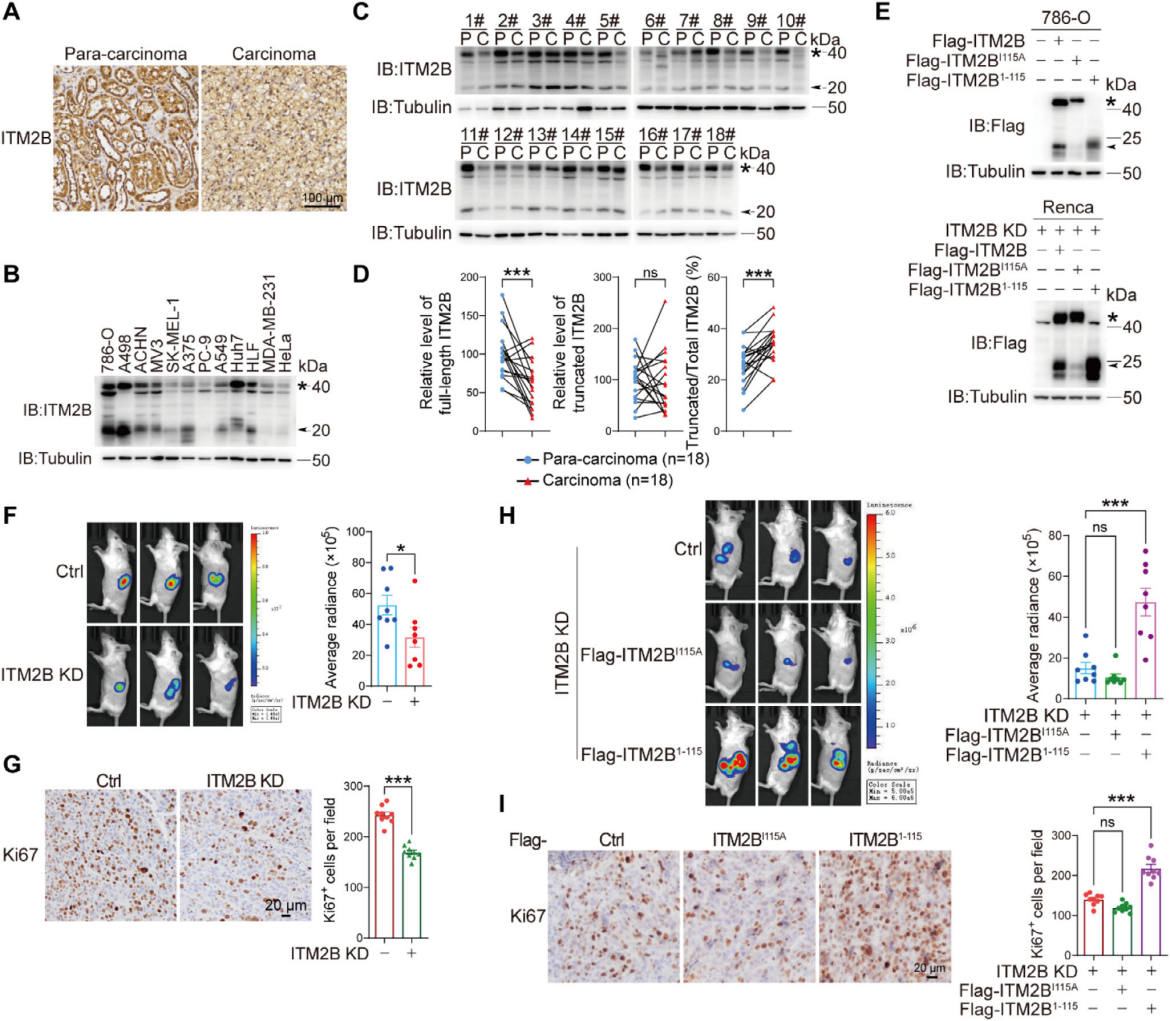
Truncated‐ITM2B promotes the growth of RCC. A) Immunohistochemical analysis of ITM2B expression in clinical RCC tissues and corresponding para‐carcinoma tissues. B) The cleavage of endogenous ITM2B was detected in various cancer cell lines. **C,D)** ITM2B expression was detected in carcinoma (abbreviated as C) and corresponding para‐carcinoma tissues (abbreviated as P) from RCC patients C). The expression levels of full‐length ITM2B and ITM2B truncation were quantified with ImageJ D) (*n* = 18 patients). E) Control (empty vector), ITM2B, ITM2B^I115A,^ and ITM2B^1‐115^ were overexpressed in 786‐O cells (top) and ITM2B‐knockdown Renca cells (bottom). **F,G)** Control and ITM2B‐knockdown luciferase‐expressing Renca cells were conducted to orthotopic allografts. Tumor growth was indicated and quantified by the luciferase signals (F, *n* = 8 mice). Ki67 expression levels were indicated, and fields (*n* = 9) from three independent tumor tissues were randomly choose to quantify Ki67 expression G). **H,I)** Indicated Renca cells were conducted to orthotopic allografts. Tumor growths (H, *n* = 8 mice) and the expression levels of Ki67 (I, *n* = 9 fields) were shown. Data were shown as the mean ± s.e.m. ^*^ *p *< 0.05, ^**^ *p *< 0.01, ^***^ *p *< 0.001.

The functions of ITM2B have long been focused on regulating neurological dysfunction, but are overlooked in the context of cancer development. Through analysis of *ITM2B* mRNA expression using the cancer genome atlas (TCGA) databases, we found significantly higher *ITM2B* mRNA levels in kidney tissues and their corresponding tumor tissues (Figure S1A, Supporting Information). In cancer cell line encyclopedia (CCLE) database,^[^ ^23^ ^]^ the renal cancer cell lines exhibited the highest ITM2B protein level among various cancer cell lines (Figure S1B, Supporting Information). Immunohistochemical analysis of ITM2B expression in carcinoma tissues and corresponding para‐carcinoma tissues of RCC patients indicated that the ITM2B protein level in carcinoma tissues decreased compared to that in para‐carcinoma tissues (**Figure** **1**A). These findings suggest the potential involvement of ITM2B in kidney tissue and related cancers, such as RCC. It is well accepted that ITM2B is expressed frequently in both full‐length and truncated forms.^[^ ^15^ ^]^ To detect different forms of ITM2B simultaneously, we generated an antibody capable of clearly detecting both endogenous full‐length (indicated by *) and truncated‐ITM2B (indicated by arrows, migrating at ≈20 kDa). The ITM2B protein levels detected in mouse further validated a higher ITM2B protein level in kidney tissue compared to other tissues (Figure S1C, Supporting Information). In various cancer cell lines, ITM2B was found to possess a broad‐spectrum cleavage property, and RCC cells such as 786‐O, A498, and ACHN exhibited relatively high ITM2B expression levels, particularly in the truncated‐ITM2B case (Figure 1B). Furthermore, we collected 18 pairs of clinical RCC samples, including renal cell carcinoma samples and corresponding para‐carcinoma samples, to analyze ITM2B expression patterns. The results revealed lower levels of full‐length ITM2B in the carcinoma samples than those in the para‐carcinoma samples, whereas the levels of truncated‐ITM2B remained high in the carcinoma samples (Figure 1C). The quantitative analysis of the western blot further supported a decreasing trend for full‐length ITM2B but not for truncated‐ITM2B in the carcinoma samples (Figure 1D, left & middle). Moreover, the ratio of truncated‐ITM2B to total ITM2B (i.e., full‐length plus truncated‐ITM2B) tended to increase (Figure 1D, right), suggesting that RCC cells tend to produce more ITM2B truncations.

To identify the cleavage sites in ITM2B, we constructed different fragment mutants. The truncation produced by ITM2B^1‐105^ was smaller than that produced by wild‐type ITM2B, whereas the truncation produced by ITM2B^1‐115^ displayed a comparable molecular weight (Figure S1D, Supporting Information). These findings suggested that the cleavage sites of ITM2B are located between site 106 and site 115. In this region, separate mutations of all residues (except for three alanine residues) into alanine (A) revealed that only ITM2B^I115A^ displayed a full‐length status with the same molecular weight as ITM2B, while truncated‐ITM2B was minimally detected (Figure S1E, Supporting Information). These results indicate that I115 in ITM2B is an unreported site for ITM2B cleavage. The amino acid sequences of human and mouse ITM2B exhibit a high degree of homology (>95%) (Figure S1F, Supporting Information), and the I115 site of ITM2B is conserved across different species (Figure S1G, Supporting Information). In both human RCC 786‐O cells and mouse RCC Renca cells, ITM2B^1‐115^ and ITM2B^I115A^ exhibited identical patterns of ITM2B cleavage (Figure 1E). We further used ITM2B^1‐115^ (which displays only truncated ITM2B) and ITM2B^I115A^ (which appears as full‐length ITM2B) to investigate their functions in RCC. In the Renca cell‐derived orthotopic renal cell carcinoma mouse model, ITM2B knockdown retarded tumor growth (Figure 1F) through the inhibition of tumor cell proliferation, as indicated by Ki67 immunostaining (Figure 1G). Furthermore, ITM2B^1‐115^ overexpression in ITM2B‐knockdown Renca cells significantly promoted tumor growth, whereas ITM2B^I115A^ did not (Figure 1H), as reflected by corresponding Ki67 expression levels (Figure 1I). Clearly, truncated ITM2B effectively promotes RCC growth.

### 2.2 Migrasomes Contribute to ITM2B Truncation‐Promoted Tumor Growth

**Figure 2:**
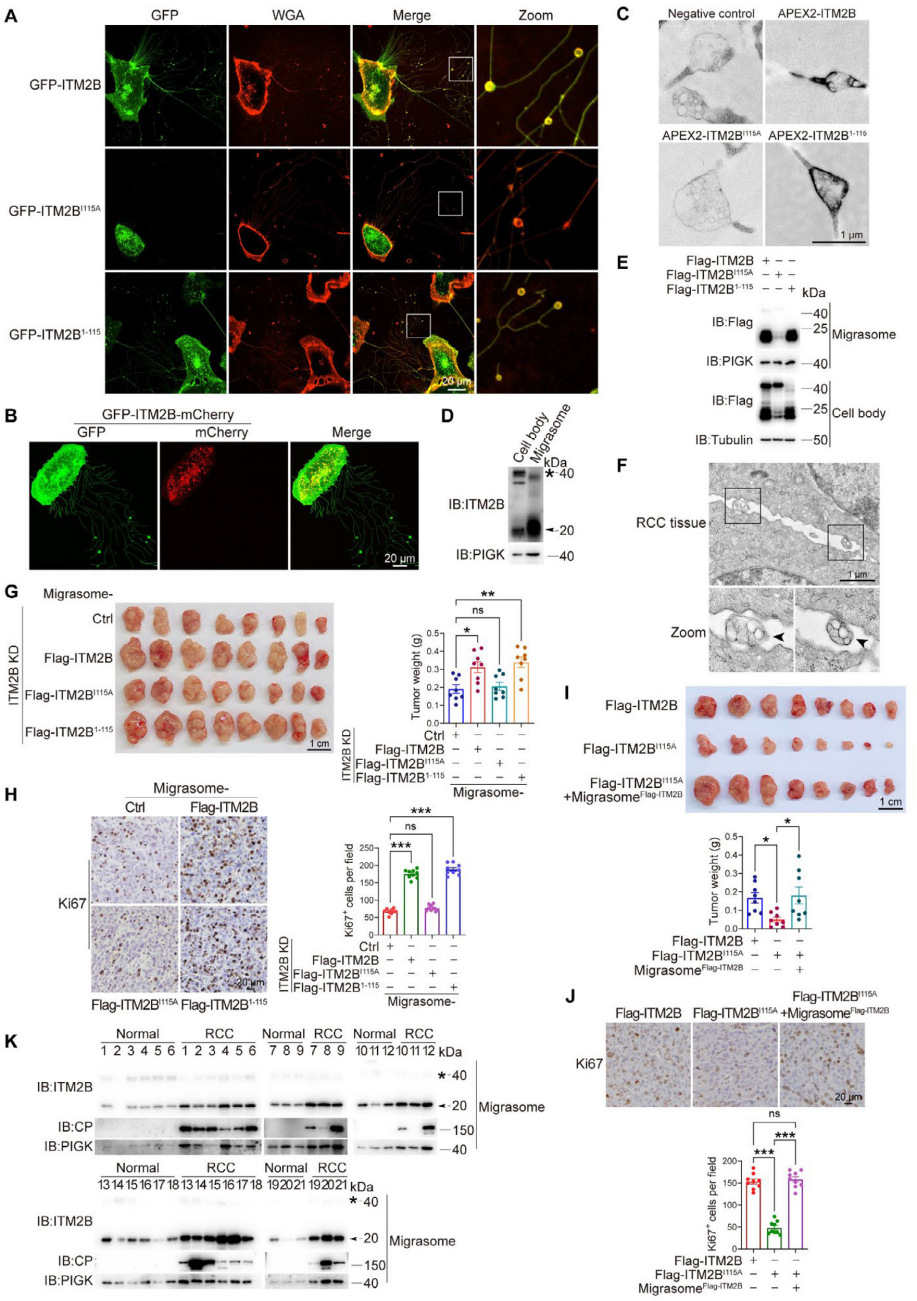
ITM2B truncation promotes RCC growth through migrasome. A) GFP‐ITM2B‐, GFP‐ITM2B^I115A^‐, and GFP‐ITM2B^1‐115^‐expressing 786‐O cells were stained with WGA. B) GFP‐ITM2B‐mCherry was expressed in 786‐O cells, live‐cell imaging was applied to visualize its subcellular location. C) APEX2‐ITM2B, APEX2‐ITM2B^I115A,^ and APEX2‐ITM2B^1‐115^ were expressed in 786‐O cells, and the APEX2 signals in migrasomes of these cells were visualized under transmission electron microscope. Empty vector without APEX2‐tag was used as a negative control. D) Cell body and migrasome of 786‐O cells were prepared, and the endogenous ITM2B was indicated. E) Flag‐tagged ITM2B, ITM2B^I115A,^ and ITM2B^1‐115^ were expressed in 786‐O cells, and the cell body and migrasome were prepared. F) Fresh tissues from orthotopic allografts were collected to visualize in situ migrasomes in allografts using electron microscopy. **G,H)** Renca cells were subcutaneously inoculated into the flank of BALB/c mice. The migrasomes derived from different groups of Renca cells as indicated were injected into peritumoral regions. Tumor growths and weights were indicated (G, *n* = 8 mice), the expression levels of Ki67 (H, *n* = 9 fields) in tumor tissues were indicated. **I,J)** Flag‐ITM2B and Flag‐ITM2B^I115A^ were reintroduced into ITM2B‐knockdown Renca cells, and the cells were subcutaneously inoculated into the flank of BALB/c mice. Migrasomes derived from Flag‐ITM2B‐expressing Renca cells were collected and injected into peritumoral regions of Flag‐ITM2B^I115A^‐expressing allografts. Tumor growths (I, *n* = 8 mice) and the expression levels of Ki67 (J, *n* = 9 fields) were indicated. K) Migrasomes in 100 mL early morning urine from normal volunteer (*n* = 21) and RCC patients (*n* = 21) were purified. Indicated proteins were detected in uric migrasomes. Data were shown as the mean ± s.e.m. ^*^ *p *< 0.05, ^**^ *p *< 0.01, ^***^ *p *< 0.001.

To further explain the unique function of truncated ITM2B in tumor growth, we first employed immunofluorescence analysis to elucidate the subcellular localization of ITM2B and its mutations. WGA (wheat‐germ agglutinin) is a fluorescent dye for indication of migrasomes^[^ ^24^ ^]^ and TSPAN4 is a protein marker for migrasomes.^[^ ^1^ ^]^ It could be shown that both GFP‐ITM2B and GFP‐ITM2B^1‐115^ displayed a remarkable colocalization with WGA‐stained or TSPAN4‐positive migrasomes at the ends or intersections of RFs, whereas ITM2B^I115A^ displayed punctate structures only within cells (**Figure** **2**A; Figure S2A, Supporting Information). Imaging of the GFP‐ITM2B‐mCherry fusion protein also revealed that the GFP‐tagged N‐terminal truncation of ITM2B (green), but not full‐length ITM2B (yellow, containing both GFP and mCherry), could be transferred to the migrasomes (Figure 2B). APEX2, a broadly applicable tag used to increase electron microscopy contrast,^[^ ^25^ ^]^ was genetically fused to ITM2B or its mutants in 786‐O cells. Electron microscopy clearly revealed the presence of APEX2 signals in migrasomes of both ITM2B‐ and ITM2B^1‐115^‐expressing cells but not in migrasomes of ITM2B^I115A^‐expressing cells (Figure 2C). These results consistently support the localization of truncated ITM2B in migrasomes. Furthermore, migrasomes were extracted from 786‐O cells, and their characteristic features were visualized using a transmission electron microscope (Figure S2B, Supporting Information). Western blotting analysis revealed that migrasome markers (CPQ and PIGK) were enriched, whereas exosome markers (CD63 and TSG101) were scarce in the migrasomes (Figure S2C, Supporting Information), thereby validating the purity of the isolated migrasomes. In the cell body fractions, both full‐length ITM2B and truncated‐ITM2B were present, whereas only truncated‐ITM2B was enriched in the migrasome fractions (Figure 2D). When different ITM2B mutants were expressed in 786‐O cells, truncated‐ITM2B was abundant in migrasomes derived from ITM2B‐ and ITM2B^1‐115^‐expressing cells but not ITM2B^I115A^‐expressing cells (Figure 2E). Furthermore, the presence of typical migrasome‐like structures was also validated in Renca cells‐derived orthotopic allografts (Figure 2F). We thus postulated that the localization of truncated‐ITM2B within migrasomes may contribute to its protumoral function.

To further verify the above proposal, different groups of migrasomes were injected subcutaneously into Renca cell‐derived allografts. Since ITM2B or its mutants did not influence the size of migrasomes (Figure S2D, Supporting Information) and migrasomes from different groups with equivalent protein mass exhibited comparable particle counts (≈1.35 × 10^8^ particles of migrasomes contain 10 µg of protein) (Figure S2E, Supporting Information), we injected migrasomes with equal protein masses into the allografts. The results indicated that migrasomes from ITM2B^I115A^‐expressing Renca cells had no effect on the growth of allografts; in contrast, migrasomes from ITM2B^1‐115^‐ or wild‐type ITM2B‐expressing Renca cells significantly promoted tumor growth (Figure 2G). Ki67 expression also showed the expected results in different groups (Figure 2H), suggesting that ITM2B truncation accelerated tumor growth through migrasomes. Additionally, compared with the subcutaneous allografts derived from ITM2B^WT^‐expressing Renca cells, ITM2B^I115A^‐expressing Renca cell‐derived allografts presented delayed tumor growth and reduced Ki67 expression; however, the injection of migrasomes derived from ITM2B^WT^‐expressing Renca cells effectively rescued tumor growth and tumor cell proliferation to the levels of ITM2B^WT^‐expressing allografts (Figure 2I,J). ITM2B or its mutants had no impact on cell cycle of RCC cells (Figure S2F, Supporting Information). These results suggest that migrasomes rescue the defect of ITM2B truncation in RCC. Collectively, a series of results in mouse models demonstrates that truncated ITM2B may promote RCC growth via migrasomes.

Because the kidney is a key organ of the urinary system, we collected urine from normal volunteers and clinical RCC patients to analyze levels of migrasomes and ITM2B expression. The results revealed that more migrasomes were present in the urine of RCC patients than in the urine of healthy volunteers (Figure S2G,H, Supporting Information). In migrasomes extracted from RCC patient urine, CP, an RCC‐specific marker,^[^ ^26^, ^27^ ^]^ was exclusively detected. Moreover, truncated ITM2B was also more abundant than it was in normal samples (Figure 2K). These results indicate for the first time that ITM2B truncation is a vital regulator of tumor migrasomes and suggest that numerous tumor cell‐derived migrasomes are produced during human RCC progression.

### 2.3 ITM2B Truncation Regulates Migrasome Formation

**Figure 3:**
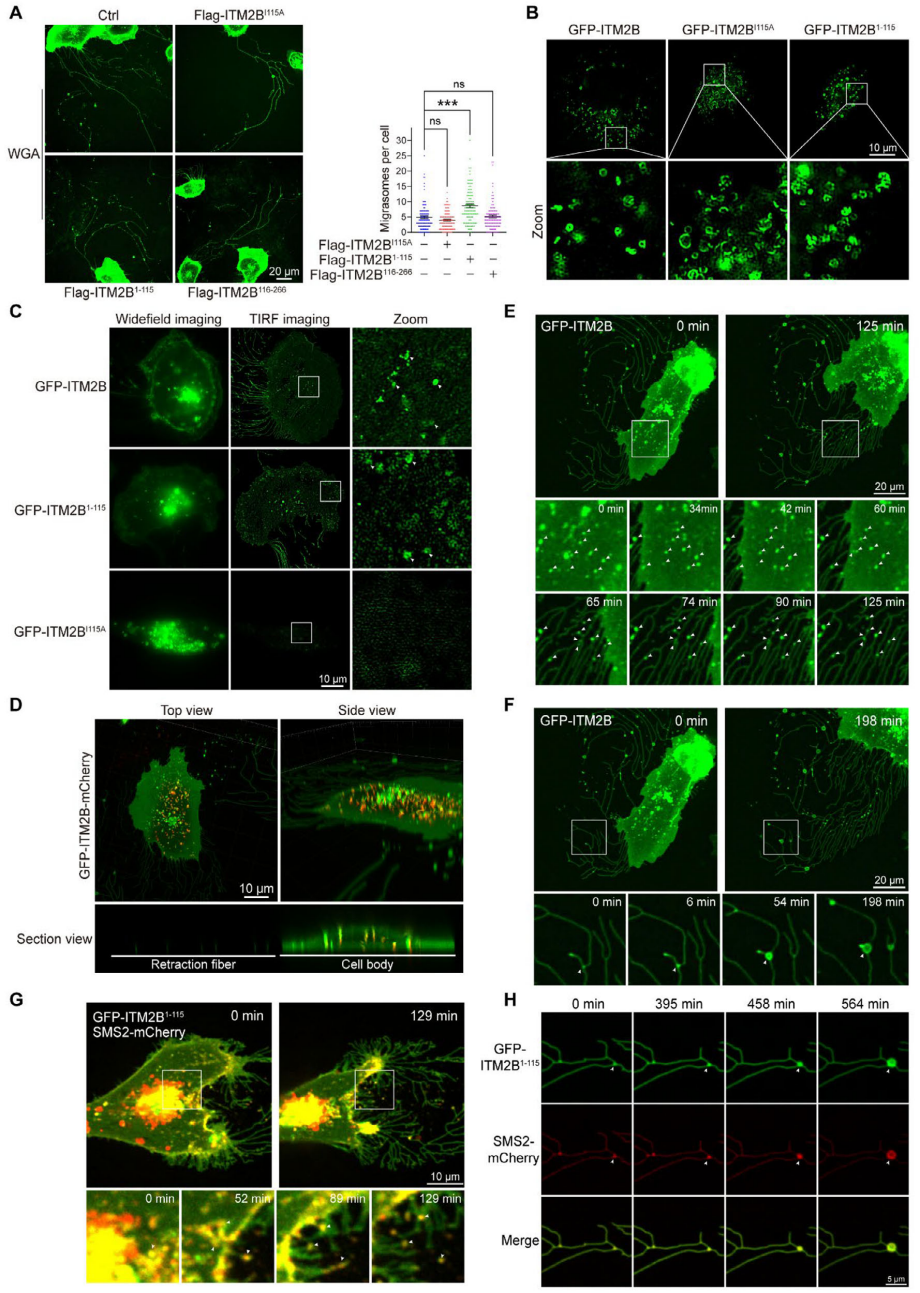
ITM2B truncation promotes migrasome formation. A) Control (empty vector), ITM2B^I115A^, ITM2B^1‐115^, and ITM2B^116‐266^ were overexpressed in 786‐O cells, and the cells were stained with WGA to show corresponding migrasomes (left). The migrasome numbers with 100 cells were counted in each group (right). B) Super‐resolution SIM was applied to visualize GFP‐positive dot structures in indicated 786‐O cells. C) GFP‐ITM2B‐, GFP‐ITM2B^I115A^‐, and GFP‐ITM2B^1‐115^‐expressing 786‐O cells were separately subjected to widefield imaging and TIRF imaging in the same cells of each group. D) 3D imaging to visualize the spatial distribution of GFP‐ITM2B‐mCherry in living 786‐O cells. **E,F)** Time‐lapse imaging of GFP‐ITM2B‐expressing 786‐O cells exhibited the transfer process of ITM2B from inside cells to RFs (E, indicated by arrows), and the process of ITM2B‐mediated migrasome swelling (F, indicated by arrows). G) GFP‐ITM2B^1‐115^ and SMS2‐mCherry were expressed in 786‐O cells. Time‐lapse imaging was applied to visualize the transfer process of ITM2B^1‐115^ (green) and SMS2 (red) from cell body to RFs. H) Time‐lapse imaging was applied to visualize the GFP‐ITM2B truncation‐ and SMS2‐positive migrasome formation. Data were shown as the mean ± s.e.m. ^*^ *p *< 0.05, ^**^ *p *< 0.01, ^***^ *p *< 0.001.

We further investigated how ITM2B truncation regulates migrasome formation. Immunostaining of cells using WGA revealed that knockdown of endogenous ITM2B led to a decrease in migrasome numbers in 786‐O cells (Figure S3A, Supporting Information). Moreover, only ITM2B^1‐115^, but not ITM2B^I115A^ or ITM2B^116‐266^ (the C‐terminal fragment after ITM2B cleavage), markedly increased the migrasome numbers compared with those of the empty vector control despite comparable expression levels of ITM2B mutants (**Figure** **3**A; Figure S3B, Supporting Information). These results suggest a novel role of ITM2B^1‐115^ truncation in promoting migrasome formation. The mechanism underlying the localization of truncated‐ITM2B in migrasomes was subsequently investigated. Super‐resolution imaging revealed that ITM2B and its mutants were present in hollow vesicles inside cells (Figure 3B). Electron microscopy also revealed that the APEX2‐ITM2B, ‐ITM2B^1‐115^ and ‐ITM2B^I115A^ signals appeared on the outer membrane of the intracellular vesicles (i.e., the cytoplasmic side) (Figure S3C, Supporting Information), which is consistent with the characteristic localization of type II transmembrane proteins, in which the N‐terminus of the protein is proximal to the cytoplasm.^[^ ^28^ ^]^ TIRF (total internal reflection fluorescence) imaging of GFP‐ITM2B‐mCherry further revealed that N‐terminal ITM2B truncation‐containing vesicles (green), but not full‐length ITM2B‐containing vesicles (yellow), could be transferred to the bottom of cells (Figure S3D, Supporting Information). Similarly, vesicles containing ITM2B^1‐115^ but not ITM2B^I115A^ were attached to the basal membrane (TIRF imaging) (Figure 3C). In the case of co‐staining of both ITM2B truncation and TSG101 (a classical marker for MVBs^[^ ^29^ ^]^), TIRF imaging demonstrated that these ITM2B truncation‐enriched vesicles are distinct from MVBs (Figure S3E). These results imply that only ITM2B^1‐115^ truncation‐containing vesicles adhere to the bottom layer of cells. Therefore, it seems that only the ITM2B truncation is transported to plasma membrane through vesicles. Indeed, 3D imaging to elucidate the spatial distribution of ITM2B revealed that full‐length ITM2B (GFP‐ITM2B‐mCherry) was distributed inside cells as vesicular structures (yellow), whereas the N‐terminal truncated‐ITM2B was located at the plasma membrane and extended to RFs and migrasomes (green, Figure 3D; Movie S1, Supporting Information).

We next applied time‐lapse imaging to investigate the transport process of ITM2B and found that numerous ITM2B‐containing intracellular vesicles adhered to the basal membrane and remained immobile. Once the cells moved away, ITM2B truncation entered RFs (Figure 3E; Movie S2, Supporting Information). As time progressed, these ITM2B truncation‐enriched sites at RFs became swollen and then developed to migrasomes (Figure 3F; Movie S3, Supporting Information). In the GFP‐ITM2B‐mCherry‐expressing cells, only N‐terminal ITM2B truncation could adhere to the cell bottom via vesicle transportation and subsequently arrived at RFs, whereas full‐length ITM2B (yellow, showing both the GFP and mCherry signals simultaneously) and C‐terminal ITM2B (red, showing the mCherry signal) remained inside the cells (Figure S3F and Movie S4, Supporting Information). Therefore, it seems that the ITM2B truncation is transferred to RFs and migrasomes through vesicle transportation.

A recent study indicated that immobile foci formed by SMS2 on the basal membrane of migrating cells determine migrasome formation sites.^[^ ^4^ ^]^ We further investigated whether ITM2B truncation‐enriched vesicles localize to SMS2 foci inside cells. Indeed, the ITM2B truncation colocalized and interacted with SMS2 inside cells (Figure S3G,H, Supporting Information). TIRF imaging confirmed that ITM2B truncation‐enriched vesicles localize to SMS2 foci on the cell basal membrane (Figure S3I, Supporting Information, GFP‐ITM2B^1‐115^‐containing vesicles (green), indicated by arrows, SMS2‐mCherry foci (red)). As cells migrated, ITM2B^1‐115^ and SMS2 remained and entered RFs (Figure 3G; Movie S5, Supporting Information), subsequently facilitating migrasome swelling on RFs (Figure 3H; Movie S6, Supporting Information). These results indicate that ITM2B truncation is transferred to predefined migrasome formation sites (i.e., SMS2 foci) at the basal membrane of migrating cells, where it contributes to migrasome swelling and formation.

### 2.4 ITM2B Truncation Promotes Migrasome Swelling Through the Recruitment of TSPAN4

**Figure 4:**
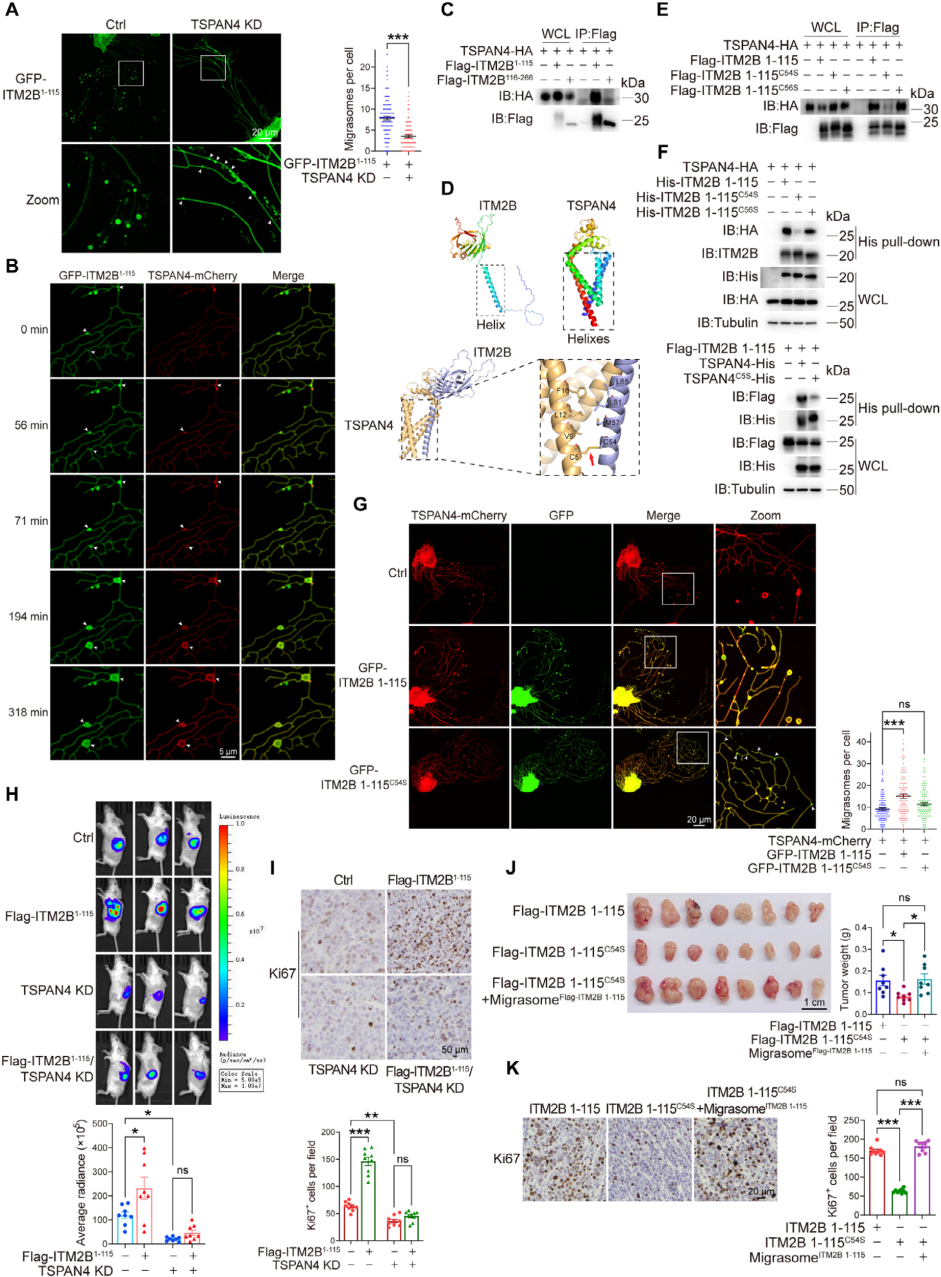
ITM2B truncation recruits TSPAN4 to facilitate migrasome formation. A) TSPAN4 was knocked down in GFP‐ITM2B^1‐115^‐expressing 786‐O cells, live‐cell imaging was applied to observe the GFP‐ITM2B^1‐115^ migrasomes (left). Arrows indicate the GFP‐ITM2B^1‐115^ puncta on RFs. Migrasome numbers were counted with 100 cells in each group (right). B) GFP‐ITM2B^1‐115^ and TSPAN4‐mCherry were expressed in 786‐O cells, time‐lapse imaging indicated GFP‐ITM2B^1‐115^ enriched at migrasome formation sites prior to TSPAN4‐mCherry. C) ITM2B fragments and TSPAN4 were expressed in 786‐O cells, their interactions were detected. D) Top, predicted structures of ITM2B and TSPAN4 by AlphaFold2. Bottom, molecular modeling of ITM2B binding to TSPAN4. E) ITM2B truncation or its point mutants and TSPAN4 were expressed in 786‐O cells, their interactions were detected. F) TSPAN4, together with ITM2B truncation and their point mutants as indicated, were expressed in 786‐O cells. The His pull‐down assay indicated the covalent binding between ITM2B truncation and TSPAN4. G) Control (empty vector), GFP‐ITM2B 1–115 and GFP‐ITM2B 1–115^C54S^ were separately transfected into the TSPAN4‐mCherry‐expressing 786‐O cells (left). Arrows indicated GFP‐ITM2B 1–115^C54S^ puncta without TSPAN4 enrichment on retraction fibers. Migrasome numbers were counted with 100 cells in each group (right). **H,I)** Flag‐ITM2B^1‐115^ was overexpressed in TSPAN4‐knockdown luciferase‐expressing Renca cells. The cells were orthotopically injected into mice. Tumors in kidney (H, *n* = 8 mice) and the expression levels of Ki67 (I, *n* = 9 fields) were indicated. **J,K)** Flag‐ITM2B 1–115 and Flag‐ITM2B 1–115^C54S^ were reintroduced into ITM2B‐knockdown Renca cells, and the cells were subcutaneously inoculated into the flank of BALB/c mice. Migrasomes derived from Flag‐ITM2B 1‐115‐expressing Renca cells were collected and injected into peritumoral regions of Flag‐ITM2B 1–115^C54S^‐expressing allografts. Tumor growths and weights (J, *n* = 8 mice) and the expression levels of Ki67 (K, *n* = 9 fields) were indicated. Data were shown as the mean ± s.e.m. ^*^ *p *< 0.05, ^**^ *p *< 0.01, ^***^ *p *< 0.001.

To elucidate how ITM2B truncation promotes migrasome swelling, a series of experiments was conducted. First, we observed a subset of ITM2B^1‐115^‐enriched vesicles moving toward the intersections of RFs (Figure S4A and Movie S7, Supporting Information), which further facilitated migrasome swelling (Figure S4B and Movie S8, Supporting Information), suggesting that these truncated ITM2B‐enriched vesicles may provide some materials for migrasome swelling. Second, given that different tetraspanins (TSPANs) enhance migrasome formation,^[^ ^5^ ^]^ we assessed the mRNA expression levels of seven main TSPANs in RCC cells and found that TSPAN4 had the highest mRNA level in both human and mouse RCC cells (Figure S4C, Supporting Information). The assembly of the TSPAN4‐enriched macrodomain is essential for membrane swelling and migrasome formation.^[^ ^5^, ^6^ ^]^ Live‐cell imaging revealed that TSPAN4 knockdown greatly impaired migrasome formation in the context of ITM2B^1‐115^ overexpression (**Figure** **4**A), as evidenced by reduced migrasome swelling even in the ITM2B^1‐115^‐enriched RFs (Figure 4A, zoom, indicated by arrow). Moreover, time‐lapse imaging revealed that ITM2B^1‐115^ enriched in migrasome formation sites before TSPAN4 (Figure 4B; Movie S9, Supporting Information). It is plausible that ITM2B^1‐115^ localization at migrasome formation sites may recruit TSPAN4 to these sites, subsequently initiating migrasome swelling.

We further investigated the interaction pattern between ITM2B and TSPAN4. A co‐IP assay revealed a strong interaction of TSPAN4 with ITM2B^1‐115^ but not with ITM2B^116‐266^ (Figure 4C), suggesting that the ITM2B^1‐115^ truncation is capable of recruiting TSPAN4. ITM2B and TSPAN4 contain one and four transmembrane helical structural domains, respectively.^[^ ^5^, ^15^ ^]^ We employed AlphaFold2 to generate their respective structures (Figure 4D, top). To identify the key residues responsible for their interaction, we generated a fusion protein with a 200‐glycine junction using AlphaFold2 for structure prediction and Rosetta for structural relaxation. The predicted structure of the complex indicates that Cys5 of TSPAN4 and Cys54 of ITM2B potentially form a disulfide bridge, thereby ensuring robust binding capacity between these two proteins (Figure 4D, bottom). Given the conservation of both Cys5 of TSPAN4 and Cys54 of ITM2B across different species (Figure S4D), these cysteines may play crucial roles in mediating their interaction. To validate these predicted sites, mutations with Cys5 of TSPAN4 and Cys54 of ITM2B were separately introduced. Co‐IP assays revealed that the ITM2B 1–115^C54S^ mutant (based on the ITM2B^1‐115^ truncation) effectively disrupted the interaction between ITM2B^1‐115^ and TSPAN4 (Figure 4E). In a pull‐down assay, washing buffer containing 8 m urea was used to provide a denaturation environment to prevent noncovalent binding between proteins. The results demonstrated the presence of covalent binding between ITM2B truncation and TSPAN4, as reducing agents such as β‐ME and TCEP could destroy this covalent binding (Figure S4E). Furthermore, both the Cys54 mutant of the ITM2B truncation (ITM2B 1–115^C54S^) and the Cys5 mutant of TSPAN4 (TSPAN4^C5S^) effectively abolished this covalent binding (Figure 4F). These results confirm the disulfide bond‐dependent interaction between Cys54 of ITM2B and Cys5 of TSPAN4. Consequently, ITM2B^1‐115^ overexpression significantly promoted migrasome formation, whereas the Cys54 mutation in ITM2B truncation mutants failed to do so (Figure 4G). Moreover, ITM2B 1–115^C54S^ resulted in more TSPAN4‐negative puncta in RFs (Figure 4G, zoom, indicated by arrows), indicating that the impairment of the Cys54 mutant in the ITM2B truncation led to the recruitment of TSPAN4. Clearly, the interaction between ITM2B truncation and TSPAN4 is critical for promoting migrasome swelling and formation.

Finally, we determined the role of TSPAN4‐dependent migrasome formation in the protumoral function of ITM2B truncation. In orthotopic allografts, ITM2B^1‐115^ overexpression in Renca cells notably promoted tumor growth, accompanied by increased Ki67 expression, whereas the knockdown of TSPAN4 in Renca cells to inhibit migrasome formation blocked the protumoral function of ITM2B^1‐115^, with decreased Ki67 expression levels (Figure 4H,I). Furthermore, ITM2B 1–115^C54S^‐expressing Renca cell‐derived subcutaneous allografts, which are deficient in migrasome formation, exhibited delayed tumor growth and decreased Ki67 expression compared with ITM2B 1‐115‐expressing allografts. The administration of migrasomes from ITM2B 1‐115‐expressing Renca cells effectively rescued the defects of ITM2B 1–115^C54S^‐expressing allografts in mice (Figure 4J,K). Together, these data support the role of TSPAN4 in combination with ITM2B truncation to promote RCC growth through migrasomes.

### 2.5 ITM2B Truncation‐containing Vesicles Selectively Sort Active Caspase‐7 into Migrasomes

**Figure 5:**
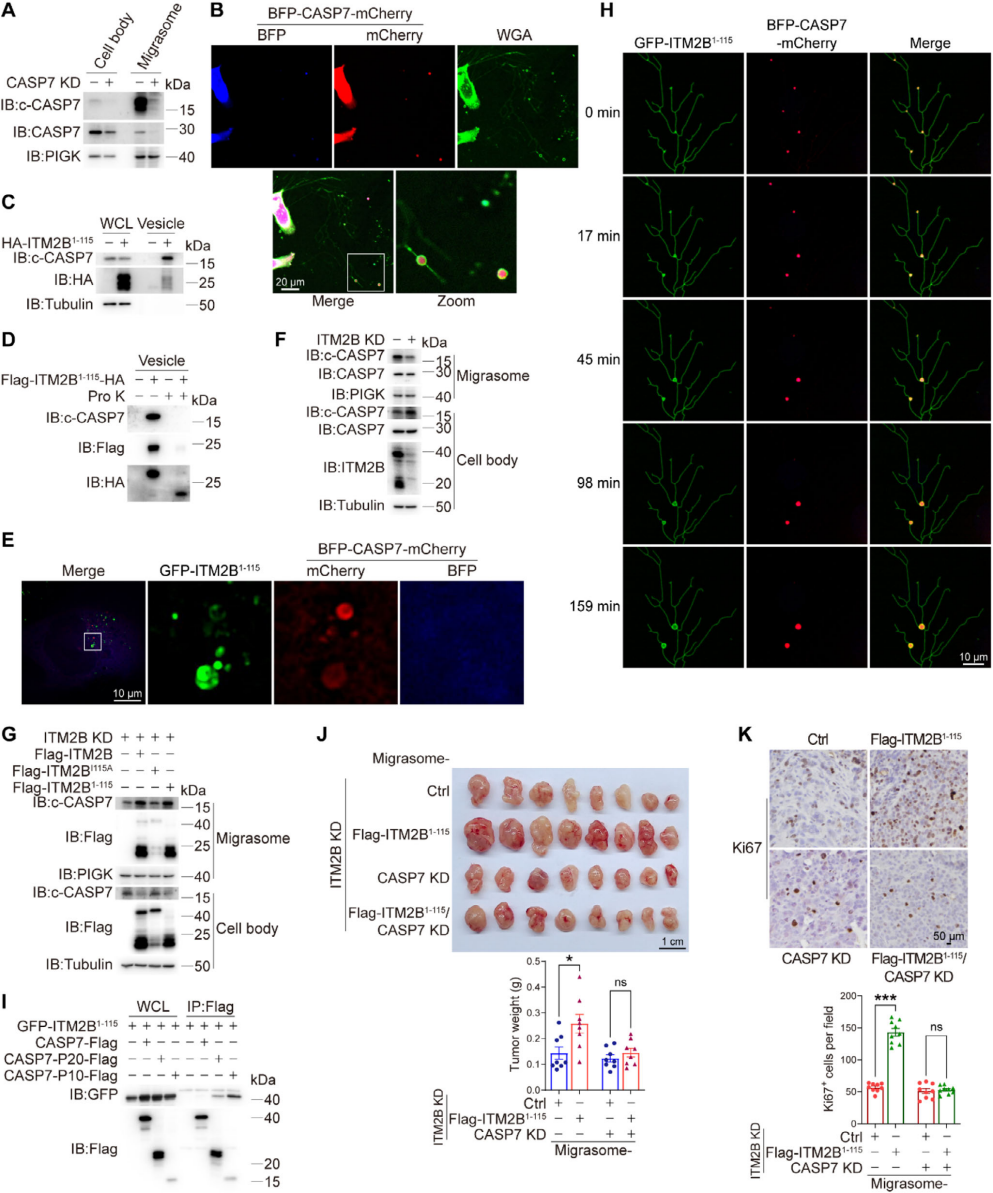
ITM2B Truncation Promotes the Migracytosis of Active Caspase‐7. A) The cell body and migrasome were prepared in 786‐O cells with or without caspase‐7 knockdown, the expression levels of c‐CASP7 and CASP7 were detected. B) BFP‐CASP7‐mCherry‐expressing 786‐O cells were stained with WGA, and live‐cell imaging was applied to show the different statuses of caspase‐7 in cell body and migrasome. C) HA‐ITM2B^1‐115^ was stably expressed in 786‐O cells, and HA‐ITM2B^1‐115^‐containg vesicles were isolated via immunocapture and detected by western blotting. D) Flag‐ITM2B^1‐115^‐HA was stably expressed in 786‐O cells. Anti‐Flag magnetic beads were used to immunocapture Flag‐ITM2B^1‐115^‐HA‐containing vesicles, and the vesicles were incubated with protease K in vitro. E) GFP‐ITM2B^1‐115^ and BFP‐CASP7‐mCherry were expressed in 786‐O cells, and super‐resolution SIM was applied to visualize their subcellular locations. F) The cell body and migrasome from indicated 786‐O cells were prepared, the levels of c‐CASP7 and ITM2B were detected. G) Control (empty vector), ITM2B, ITM2B^I115A^, and ITM2B^1‐115^ were reintroduced into ITM2B‐knockdown 786‐O cells. The cell body and migrasome were prepared. H) GFP‐ITM2B^1‐115^ and BFP‐CASP7‐mCherry were expressed in 786‐O cells. Time‐lapse imaging indicated c‐CASP7 accumulation accompanying with GFP‐ITM2B^1‐115^ enrichment in migrasomes. I) ITM2B^1‐115^, together with CASP7 or its subunits, was expressed in 786‐O cells, the interaction between ITM2B and CASP7 or its subunits was determined. **J,K)** Renca cells were subcutaneously inoculated into the flank of BALB/c mice. The migrasomes derived from different groups of Renca cells as indicated were injected into peritumoral regions. Tumor growths and weights (J, *n* = 8 mice), the expression levels of Ki67 (K, *n* = 9 fields) were indicated. Data were shown as the mean ± s.e.m. ^*^ *p *< 0.05, ^**^ *p *< 0.01, ^***^ *p *< 0.001.

If ITM2B truncation‐containing intracellular vesicles adhere to the basal membrane of cells for subsequent transfer into migrasomes, they may serve as carriers to sort certain factors (proteins) from the cell body to migrasomes. To verify this hypothesis, we carried out label‐free quantitative proteomics to compare the migrasome proteins changed between control and ITM2B‐knockdown 786‐O cells, as well as those between ITM2B‐knockdown 786‐O cells and the corresponding 786‐O cells that were re‐introduced with ITM2B^1‐115^. These altered proteins separately represent the potential factors regulated by endogenous ITM2B and overexpressed ITM2B truncation. Concurrently, we isolated ITM2B truncation‐containing intracellular vesicles and performed mass proteomic analysis to identify proteins that were carried by these vesicles. Through a collaborative analysis of the reduced proteins in ITM2B‐knockdown migrasomes (Figure S5A, Supporting Information, red circle), increased proteins in overexpressed ITM2B^1‐115^ migrasomes (Figure S5A, Supporting Information, blue circle) and the proteins present in the vesicles (Figure S5A, Supporting Information, green circle), we identified 78 overlapping proteins (Figure S5A,B, Supporting Information) that might be selectively sorted into migrasomes through ITM2B truncation‐containing intracellular vesicles. Among these proteins, caspase‐7, one of the most significantly decreased in ITM2B‐knockdown migrasomes (Figure S5C, Supporting Information), attracted our attention. We unexpectedly found that active caspase‐7 (i.e., c‐CASP7, cleaved caspase‐7), rather than full‐length caspase‐7 (CASP7), was enriched in migrasomes derived from both human and mouse RCC cells (**Figure** **5**A; Figure S5D, Supporting Information), suggesting a preferential sorting mechanism for active caspase‐7 by ITM2B truncation‐enriched migrasomes.

Given that caspase‐7 activation requires proteolytic removal of the N‐terminal peptide,^[^ ^30^ ^]^ we utilized a BFP‐CASP7‐mCherry fusion protein to indicate the localization of caspase‐7, in which active caspase‐7 contains mCherry alone and full‐length caspase‐7 contains both BFP and mCherry. The cell body clearly exhibited full‐length caspase‐7 (purple, overlapped by BFP and mCherry), whereas the migrasomes clearly exhibited active caspase‐7 (red, zoom) (Figure 5B; Figure S5E, Supporting Information). To further determine whether ITM2B truncation‐containing intracellular vesicles could serve as carriers to transport active caspase‐7 from the cell body to the migrasome, we used high‐affinity magnetic immunocapture to purify ITM2B^1‐115^‐containing vesicles, in which c‐CASP7 was enriched in ITM2B^1‐115^‐containing vesicles (Figure 5C). Furthermore, the purified Flag‐ITM2B^1‐115^‐HA‐containing vesicles were subjected to incubation with protease K in vitro. The results showed that c‐CASP7 and Flag‐tagged N‐terminal ITM2B^1‐115^ were sensitive to protease K digestion; in contrast, HA‐tagged C‐terminal ITM2B^1‐115^ exhibited resistance (Figure 5D). These findings suggest that active caspase‐7, similar to the N‐terminus of the ITM2B^1‐115^ truncation, is localized on the external surface of ITM2B truncation‐containing vesicles. Super‐resolution imaging further confirmed the colocalization of c‐CASP7 (red) with ITM2B^1‐115^‐containing vesicles (green), which exhibited hollow structures (Figure 5E), supporting that c‐CASP7 is localized on the outer surface of these vesicles. Therefore, it is likely that active caspase‐7 selectively associates with the outer surface of ITM2B truncation‐containing vesicles and is ultimately sorted into migrasomes.

We next investigated whether ITM2B is essential for active caspase‐7 sorting into migrasomes. When ITM2B was knocked down, c‐CASP7 levels in the migrasomes obviously decreased, whereas it increased in the cell bodies (Figure 5F). In ITM2B‐knockdown cells, ITM2B^1‐115^ overexpression effectively facilitated the sorting of c‐CASP7 into migrasomes, similar to ITM2B, whereas ITM2B^I115A^ failed to do so (Figure 5G). Time‐lapse imaging revealed that with ITM2B‐mediated migrasome swelling, more c‐CASP7 accumulated in migrasomes (Figure 5H; Movie S10, Supporting Information). These results consistently support that ITM2B truncation is critical for active caspase‐7 sorting into migrasomes.

During caspase‐7 activation, full‐length caspase‐7 undergoes cleavage into the p20 and p10 subunits, which subsequently assemble into the active caspase‐7 homodimer.^[^ ^30^ ^]^ Co‐IP assays revealed that ITM2B^1‐115^ strongly interacted with the p10 subunit of CASP7 but not with full‐length CASP7 (Figure 5I), implying that ITM2B truncation may selectively tether active caspase‐7 to the outer surface of vesicles. The knockdown of TSPAN4 led to a decrease in c‐CASP7‐containing migrasome formation and an increase in c‐CASP7 in cell bodies (Figure S5F, Supporting Information). However, the interaction between ITM2B^1‐115^ and the p10 subunit of CASP7 was not affected by knocking down TSPAN4 (Figure S5G, Supporting Information), further emphasizing that TSPAN4 is specifically responsible for membrane swelling but does not affect ITM2B truncation‐mediated active caspase‐7 tethering to vesicles. As expected, the Cys54 mutation of ITM2B^1‐115^ did not impair its ability to tether c‐CASP7 to vesicles through interaction (Figure S5H,I, Supporting Information). These results demonstrated that the interaction with active caspase‐7 facilitates ITM2B truncation ability of tethering active caspase‐7 to the outer surface of vesicles within the cell body (Figure S5J, Supporting Information), thereby promoting the entry of active caspase‐7 into migrasomes through ITM2B truncation‐containing vesicles. Therefore, in addition to regulating migrasome formation, ITM2B truncation also serves as a carrier for sorting cargoes into migrasomes.

Finally, the effects of ITM2B truncation‐induced active caspase‐7 migracytosis on tumor growth were also determined. Although migrasomes generated from re‐expressing ITM2B^1‐115^ in ITM2B‐knockdown cells accelerated tumor growth and Ki67 expression, knocking down caspase‐7 in parallel cells abolished these functions of ITM2B^1‐115^ (Figure 5J,K), which not only confirmed the protumoral roles of active caspase‐7 but also suggested that ITM2B truncation facilitated tumor growth, probably by facilitating active caspase‐7 migracytosis.

### 2.6 Migracytosis of Active Caspase‐7 Induces IL‐6 Secretion from Macrophages

**Figure 6:**
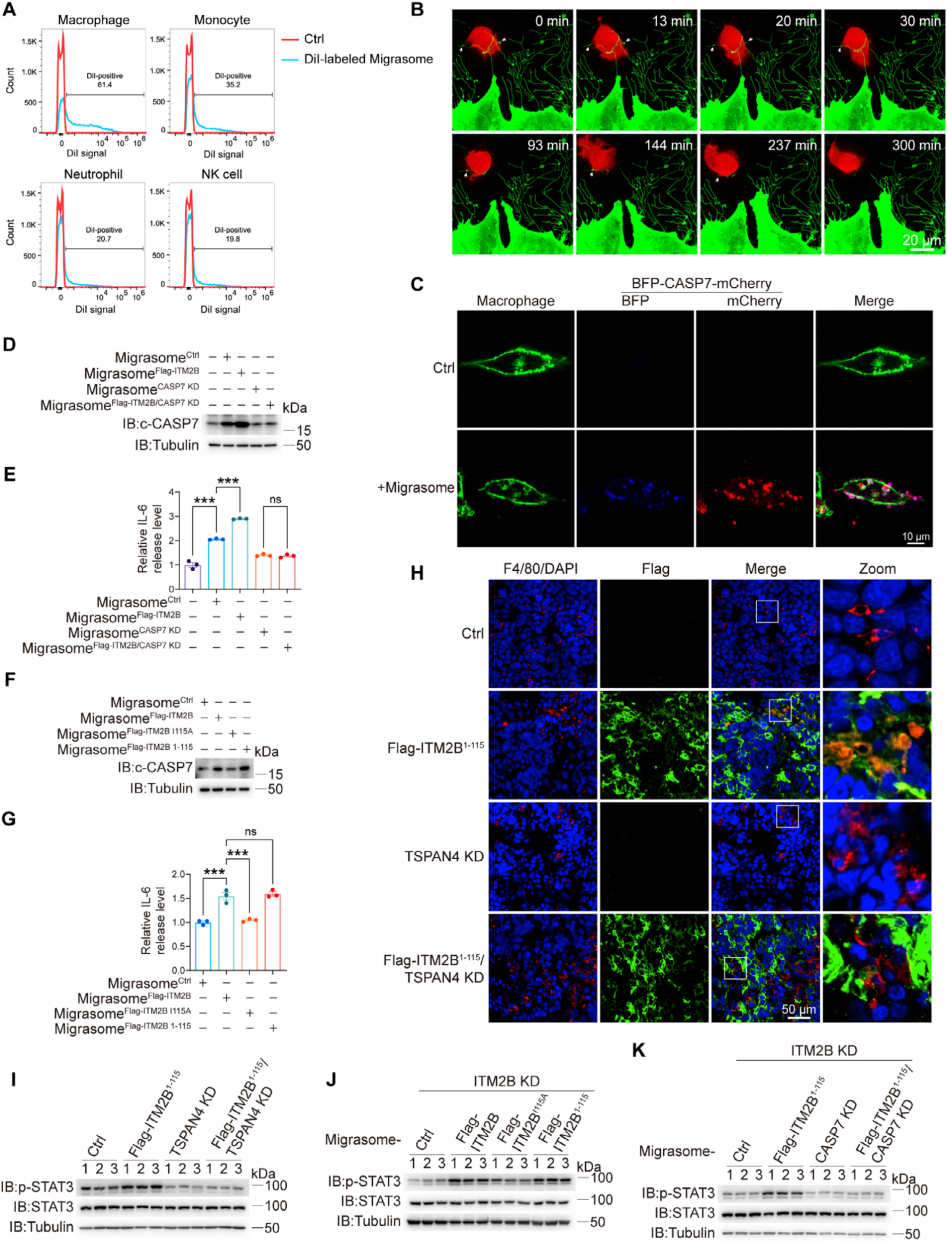
Active caspase‐7 from RCC cells promotes IL‐6 secretion from macrophages. A) Cells as indicated were incubated with DiI‐labeled migrasomes for 12 h, and then the DiI signals were detected by flow cytometry. HL‐60 cells were incubated with 1.3% DMSO for 5 days to differentiate into neutrophils. THP‐1 cells were incubated with PMA to differentiate into macrophages. B) mCherry‐expressing THP‐1 cells were pretreated with PMA (100 ng mL^−1^) for 24 h to differentiate into macrophages, then GFP‐ITM2B^1‐115^‐expressing 786‐O cells were co‐cultured with macrophages for 16 h. Time‐lapse imaging was applied to visualize the uptake of tumor cell‐derived migrasomes by macrophages. C) Migrasomes derived from BFP‐CASP7‐7‐mCherry‐expressing 786‐O cells were used to incubated with THP‐1‐differentiated macrophages for 24 h. WGA was used to stain the macrophages. **D,E)** THP‐1‐differentiated macrophages were incubated with migrasomes derived from ITM2B‐overexpressing or CASP7‐knockdown 786‐O cells for 24 h, the expression levels of c‐CASP7 were detected D). The culture mediums of indicated cells were collected to detect the levels of IL‐6 secretion (E, *n* = 3). **F,G)** Macrophages were incubated with migrasomes derived from overexpressing different ITM2B mutants as indicated in 786‐O cells. the expression levels of c‐CASP7 F) and corresponding IL‐6 secretion (G, *n* = 3) were detected. **H,I)** Tumor tissues from Figure 4H were stained separately with F4/80 (macrophage maker), Flag‐antibody, and DAPI (nucleus) to indicate the uptake of Flag‐ITM2B^1‐115^ migrasomes by macrophages H). The activation of IL‐6/STAT3 pathway in tumor tissues from Figure 4H were detected I). J) The activation of IL‐6/STAT3 pathway in tumor tissues from Figure 2G was detected. K) The activation of IL‐6/STAT3 pathway in tumor tissues from Figure 5J was detected. Data were shown as the mean ± s.e.m. ^*^ *p *< 0.05, ^**^ *p *< 0.01, ^***^ *p *< 0.001.

The functions of ITM2B truncation in assisting active caspase‐7 migracytosis were further determined. Considering the critical roles of migrasomes in intercellular communication, ITM2B truncation‐dependent migrasomes from RCC cells may be internalized by other cell types within the tumor microenvironment (TME). We purified migrasomes from 786‐O cells and then the migrasomes were stained with DiI dye, a widely used dye for membrane staining.^[^ ^31^ ^]^ When the DiI‐labeled migrasomes were incubated with different immune cell lines for 12 h, macrophages that frequently accumulate in the TME exhibited the strongest DiI signal (**Figure** **6**A), suggesting that macrophages have the highest ability to internalize RCC cells‐derived migrasomes among these immune cells. Indeed, time‐lapse imaging revealed that ITM2B^1‐115^‐containing migrasomes were taken up by macrophages (Figure 6B; Movie S11, Supporting Information). Importantly, when migrasomes obtained from BFP‐CASP7‐mCherry‐expressing 786‐O cells were extracted and incubated with macrophages (green), the results clearly indicated that the macrophages also absorbed c‐CASP7 (red) originally from 786‐O cells (Figure 6C). These results provide evidence that ITM2B‐induced migracytosis of active caspase‐7 from RCC cells may impact the function of macrophages in the TME.

As caspase‐7 participates in cell death of various cell types and inflammation of macrophages,^[^ ^30^ ^]^ we first examined whether active caspase‐7‐enriched migrasomes induce cell death in macrophages and other stroma cells. The results revealed that incubation with active caspase‐7‐enriched migrasomes derived from 786‐O cells did not cause death in various cell lines (Figure S6A, Supporting Information). It has been reported that active caspase‐7 cleaves PARP1 in macrophages to increase proinflammatory gene expression.^[^ ^32^ ^]^ Among these proinflammatory factors, IL‐6 is recognized as a highly multifunctional cytokine in the TME that exacerbates cancer development.^[^ ^33^ ^]^ Analysis of publicly available single‐cell RNA sequencing (scRNA‐seq) data revealed that macrophages are the predominant cell type expressing IL‐6 in clinical RCC (Figure S6B, Supporting Information) and that IL‐6 expression is negatively correlated with the prognosis of clinical RCC patients according to TCGA data (Figure S6C, Supporting Information). As expected, c‐CASP7‐enriched migrasomes derived from 786‐O cells induced the cleavage of PARP1 in macrophages (Figure S6D, Supporting Information), leading to elevated IL‐6 mRNA and protein expression, as well as IL‐6 secretion; however, when incubated with CASP7‐deficient migrasomes, macrophages failed to exert these effects (Figure S6E,F, Supporting Information). Active caspase‐7 in Renca cells‐derived migrasomes also induced IL‐6 expression and secretion in primary mouse macrophages (Figure S6G,H, Supporting Information). Furthermore, we incubated macrophages with intact migrasomes or lysed migrasomes (destroyed by ultrasound to release active caspase‐7), and found that only intact migrasomes could increase the level of active caspase‐7 in macrophages (Figure S6I, Supporting Information). The use of Z‐VAD to block endogenous c‐CASP7 production did not alter the increase in c‐CASP7 levels in macrophages upon incubation with 786‐O‐derived migrasomes (Figure S6J, Supporting Information), suggesting the direct transfer of active caspase‐7 protein from RCC cell‐derived migrasomes to macrophages. Together, these results demonstrate that the migracytosis of active caspase‐7 from RCC cells facilitates IL‐6 expression and secretion from macrophages.

We further investigated the role of ITM2B in regulating IL‐6 secretion by macrophages through active caspase‐7 migracytosis in RCC cells. Macrophages were incubated with migrasomes extracted from different groups of 786‐O cells. The overexpression of ITM2B, which enhances the migracytosis of c‐CASP7 in 786‐O cells, resulted in increased c‐CASP7 levels in macrophages and IL‐6 secretion from macrophages. Conversely, caspase‐7 knockdown in 786‐O cells blocked ITM2B‐induced c‐CASP7 production in macrophages and IL‐6 secretion from macrophages (Figure 6D,E). Furthermore, the overexpression of both ITM2B and ITM2B^1‐115^, but not ITM2B^I115A^, induced increased c‐CASP7 migracytosis, which was taken up by macrophages, resulting in increased IL‐6 secretion (Figure 6F,G). Similar phenomena were also validated in primary mouse macrophages incubated with mouse RCC Renca cells‐derived migrasomes (Figure S6K–N, Supporting Information). These results demonstrate that ITM2B truncation‐induced active caspase‐7 migracytosis in RCC cells can be taken up by macrophages, leading to increased IL‐6 secretion to remodel the TME.

The uptake of migrasomes derived from RCC cells by macrophages was also observed in orthotopic allograft tumor tissues, as shown in Figure 4H. The signal of Flag‐ITM2B^1‐115^, which was originally expressed in Renca cells, could be clearly detected in macrophages within the TME (Figure 6H). Importantly, this transfer of Flag‐ITM2B^1‐115^ from tumor cells to macrophages could be effectively intercepted by knocking down TSPAN4 in tumor cells (Figure 6H). Given that the secretory cytokine IL‐6 binds to its receptor to activate downstream STAT3 phosphorylation,^[^ ^34^ ^]^ we used phosphorylated STAT3 as an indicator of IL‐6 signaling activity in the TME. The overexpression of ITM2B^1‐115^ resulted in elevated p‐STAT3 levels in tumor tissues; again, this increase could be abolished by TSPAN4 knockdown (Figure 6I). These results provide compelling evidence for the occurrence of migrasome‐mediated lateral protein transfer between tumor cells and macrophages in vivo. Moreover, injection of migrasomes derived from Renca cells expressing ITM2B^1‐115^ but not ITM2B^I115A^ effectively activated IL‐6 signaling in tumor tissues from corresponding allografts (Figures 2G and 6J). Additionally, the knockdown of caspase‐7 in Renca cells impeded the ability of Renca cell‐derived migrasomes to activate IL‐6 signaling in allografts (Figures 5J and 6K). Considering the well‐established protumoral effects of IL‐6 signaling on tumor growth,^[^ ^33^, ^35^ ^]^ it can be concluded that ITM2B truncation‐dependent migrasomes transfer active caspase‐7 from RCC cells to macrophages, where active caspase‐7 promotes IL‐6 secretion and subsequently activates IL‐6 signaling in RCC in a feedback manner, thereby exacerbating RCC growth.

### 2.7 Pathological Hyperuricemia Promotes RCC Growth through Migrasome‐Mediated Mechanisms

**Figure 7:**
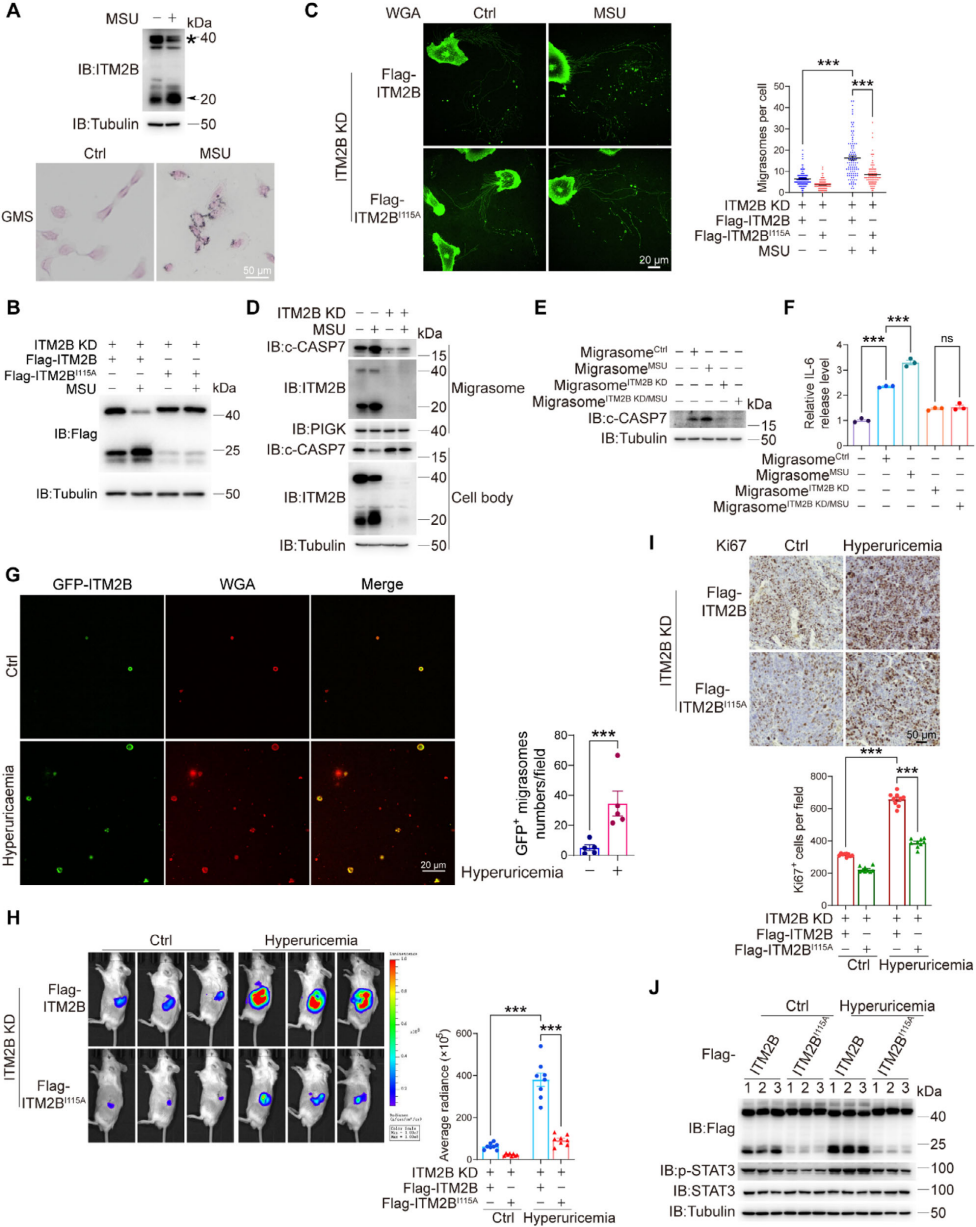
Hyperuricemia facilitates ITM2B cleavage in RCC Cells. A) 786‐O cells were treated with MSU crystal (400 µg mL^−1^) for 3 h and then cultured with fresh medium until 12 h, the endogenous ITM2B expression was indicated (top). Gomori's methenamine silver (GMS) was applied to stain the urate crystals in 786‐O cells (bottom). B) Flag‐ITM2B‐ or Flag‐ITM2B^I115A^‐expressing 786‐O cells were treated with MSU crystal, ITM2B cleavage was detected. C) Flag‐ITM2B‐ or Flag‐ITM2B^I115A^‐expressing 786‐O cells were treated with MSU crystal, and WGA was applied to stain migrasomes (left). Migrasome numbers with 100 cells were counted in each group (right). D) ITM2B‐knockdown 786‐O cells were treated with MSU crystal. The cell body and migrasome were collected and detected. **E,F)** THP‐1‐differentiated macrophages were incubated with migrasomes derived from indicated 786‐O cells for 24 h. The expression levels of c‐CASP7 E) and corresponding IL‐6 secretion (F, *n* = 3) were detected. G) In hyperuricemia tumor‐bearing BALB/c mice, urine was collected and incubated on FN‐precoated dishes. WGA was applied to stain migrasomes, and GFP‐positive migrasomes were counted in 200 µm × 200 µm fields (*n* = 5 mice). **H–J)** Flag‐ITM2B and Flag‐ITM2B^I115A^ were reintroduced into ITM2B‐knockdown luciferase‐expressing Renca cells. The cells were conducted to orthotopic allografts with or without hyperuricemia induction. Tumors in kidney were indicated and quantified by luciferase signals H). The expression levels of Ki67 in kidney tumor were indicated and quantify (I, *n* = 9 fields). ITM2B cleavage and the activation of IL‐6/STAT3 pathway were indicated J). Data were shown as the mean ± s.e.m. ^*^ *p *< 0.05, ^**^ *p *< 0.01, ^***^ *p *< 0.001.

The clinicopathological process associated with ITM2B cleavage remains unreported. The typical features of clinical RCC have been reported to be closely related to several syndromes, including lipid droplet accumulation,^[^ ^36^ ^]^ hyperglycemia^[^ ^37^ ^]^ and hyperuricemia.^[^ ^38^ ^]^ We thus assessed whether these pathological processes are associated with the ability of migrasomes to increase the risk of RCC and poor survival in patients. RCC cells were incubated with oleic acid (OA, which induces intracellular lipid droplet accumulation),^[^ ^39^ ^]^ high glucose (which causes hyperglycemia) or monosodium urate (MSU) crystals (which are the products of hyperuricemia).^[^ ^40^ ^]^ The MSU crystals, but not OA or high glucose, obviously promoted migrasome formation in 786‐O cells (Figure S7A,B, Supporting Information). Incubation of MSU crystals induced the crystals deposition in 786‐O cells and facilitated the cleavage of endogenous ITM2B (**Figure** **7**A). Clearly, MSU crystals potentially increase ITM2B cleavage to promote migrasome formation.

The functions of MSU crystals in ITM2B cleavage were further demonstrated. First, MSU crystals facilitated ITM2B cleavage but not ITM2B^I115A^ cleavage (Figure 7B). Consequently, MSU crystals induced migrasome formation in ITM2B‐expressing cells, whereas the increase in migrasomes was significantly attenuated in ITM2B^I115A^‐expressing cells (Figure 7C). This finding emphasizes the generation of ITM2B truncation, which is essential for the functions of MSU crystals. Furthermore, MSU crystals facilitated the migracytosis of c‐CASP7 in control 786‐O cells, which was accompanied by increased ITM2B truncation, whereas the increase in c‐CASP‐7 migracytosis was abolished when ITM2B was knocked down (Figure 7D). The incubation of macrophages with different groups of migrasomes derived from 786‐O cells indicated that MSU crystals enhanced the delivery of c‐CASP7 from RCC cells to macrophages and then promoted the secretion of IL‐6 from macrophages. However, ITM2B knockdown in 786‐O cells diminished these regulatory effects of MSU crystals (Figure 7E,F). Similar phenomena were also demonstrated in primary macrophages incubated with mouse RCC Renca cells‐derived migrasome (Figure S7C,D, Supporting Information). These results demonstrate that MSU crystals increase c‐CASP7 migracytosis via ITM2B. Taken together, MSU crystal‐induced ITM2B cleavage promotes migrasome formation and migracytosis of active caspase‐7, and the released active caspase‐7 is subsequently taken up by macrophages, leading to increased IL‐6 secretion.

We finally determined whether physiological hyperuricemia aggravated the growth of RCC tumors through ITM2B truncation in vivo. Using GFP‐ITM2B‐expressing Renca cells to establish orthotopic allografts, we clearly observed GFP‐positive migrasomes in mouse urine. A combination of potassium oxonate and hypoxanthine to induce hyperuricemia^[^ ^41^ ^]^ significantly elevated the concentration of serum uric acid and led to MSU crystals deposition in allografts (Figure S7E, Supporting Information), leading to an increase in the number of GFP‐positive migrasomes (Figure 7G). These findings demonstrate that physiological hyperuricemia promotes migrasome formation in RCC cells. To verify whether hyperuricemia aggravates RCC tumor growth through inducing ITM2B cleavage, we separately utilized ITM2B‐expressing and ITM2B^I115A^‐expressing Renca cells to inoculate orthotopic allografts, and the mice were subsequently administered with potassium oxonate and hypoxanthine for 18 days to induce hyperuricemia. Hyperuricemia effectively promoted tumor growth and Ki67 expression in ITM2B‐expressing allografts, whereas these promoting functions of hyperuricemia were attenuated in ITM2B^I115A^‐expressing allografts (Figure 7H,I), confirming the essential role of ITM2B cleavage in mediating the aggravating effect of hyperuricemia on RCC growth. Tumor tissues obtained from the corresponding allografts clearly revealed that hyperuricemia facilitated ITM2B cleavage in ITM2B‐expressing allografts but not in ITM2B^I115A^‐expressing allografts. Consequently, hyperuricemia increased IL‐6 pathway activity in ITM2B‐expressing allografts, whereas this effect was diminished in ITM2B^I115A^‐expressing allografts (Figure 7J). Together, these in vivo results demonstrate that physiological hyperuricemia‐induced ITM2B cleavage promotes tumor growth through migrasome formation and migracytosis of c‐CASP7.

## 3 Discussion

**Figure 8:**
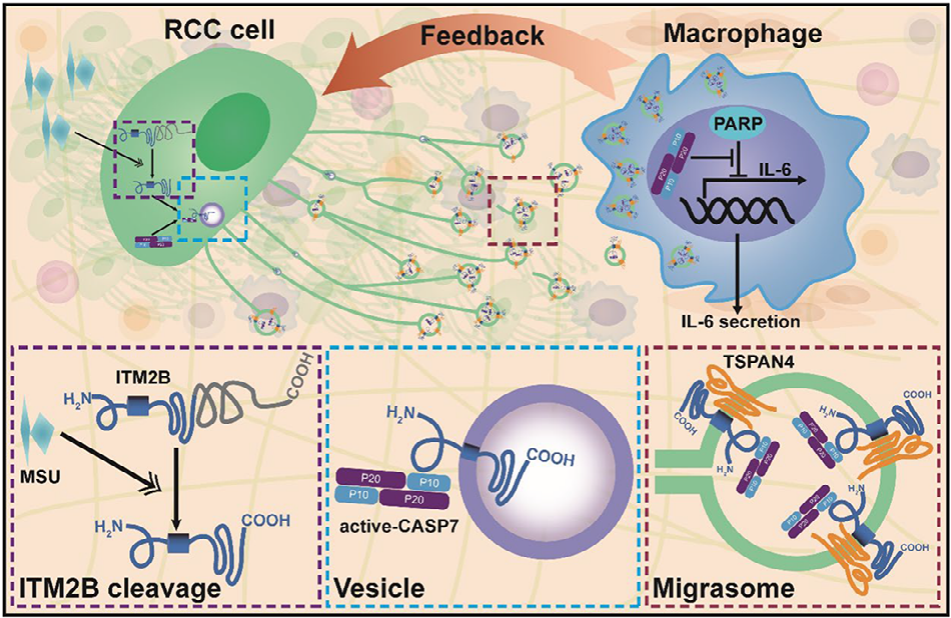
Intercellular communication between RCC Cells and macrophages.

Migrasomes have been found to act as extracellular organelles involved in intercellular communication.^[^ ^42^ ^]^ However, whether RCC cells produce migrasomes to regulate tumor progression remains unknown. Here, we demonstrated that ITM2B truncation plays a protumoral role by inducing migrasome formation and active caspase‐7 migracytosis. At the initial stage, truncated ITM2B was transferred to the migrasome formation sites via vesicle transportation and interacted with SMS2, subsequently recruiting TSPAN4 to promote migrasome swelling and formation. On the other hand, the truncated ITM2B protein interacted with active caspase‐7 to tether it to the outer surface of ITM2B truncation‐containing vesicles, thereby facilitating its sorting into migrasomes for migracytosis. As a result, the caspase‐7‐enriched migrasomes were taken up by macrophages, leading to IL‐6 secretion from macrophages to further promote RCC growth in a feedback manner (**Figure** **8**). In a mouse model, physiological hyperuricemia enhanced ITM2B cleavage to facilitate migrasome formation and activate caspase‐7 migracytosis, thereby exacerbating RCC growth. In the clinic, RCC patients were found to have increased levels of ITM2B truncation‐enriched migrasomes in their urine. Overall, this study highlights a novel role of ITM2B truncation in remodeling the TME through the induction of migrasome formation and active caspase‐7 migracytosis and explains hyperuricemia‐induced poor survival in RCC patients via the ITM2B truncation–migrasome axis.

Our data indicated that ITM2B truncation recruits TSPAN4 to migrasome formation sites through a covalent binding, thereby ensuring its effect on migrasome swelling. However, due to a lack of a commercially applicable TSPAN4 antibody, we could not further validate the endogenous interaction between ITM2B truncation and TSPAN4. In addition to promoting migrasome formation, another novel function of truncated ITM2B as a carrier to sort active caspase‐7 into migrasomes has been demonstrated. In RCC cells, the ITM2B truncation selectively interacted with the p10 subunit of active caspase‐7 but not with full‐length caspase‐7, tethering active caspase‐7 to the outer surface of ITM2B truncation‐containing vesicles. The transport process of truncated ITM2B from the vesicle to the migrasome membrane potentially involves a fusion event. Specifically, the contents within the vesicles are transferred to the extracellular compartment, while the outer surface of the vesicles is transported to the interior of the migrasome, leading to the wrapping of caspase‐7 within the migrasome. This finding is consistent with the notion of classical membrane fusion between vesicles and the plasma membrane.^[^ ^43^, ^44^ ^]^ Yu's group recently reported that secretory proteins wrapped in vesicles are transported into migrasomes and are subsequently secreted through SNARE‐mediated fusion between vesicles and the migrasome membrane.^[^ ^45^ ^]^ Together, these findings depict a pattern of vesicle‐associated cargo release via migracytosis. In fact, the transfer of vesicle cargoes to destinations includes cargo sorting, vesicle transportation and tethering process. Protein coats,^[^ ^46^ ^]^ the ESCRT complex^[^ ^47^ ^]^ and protein modifications^[^ ^31^, ^48^ ^]^ mediate cargo sorting. Rab GTPases and motor proteins can organize vesicle transportation,^[^ ^49^ ^]^ and tethering proteins, such as the NRZ complex, Golgi complex, and EEA1, tether vesicles to specific organelles.^[^ ^50^ ^]^ The mechanisms by which ITM2B truncation‐containing vesicles are directed into predefined migrasome formation sites are still a pending question and deserve further exploration.

Caspase‐7, functioning as a back‐up for caspase‐3, together with caspase‐3 itself are recognized as apoptotic executioners.^[^ ^51^ ^]^ However, a growing body of evidence suggests that the function of caspase‐7 differs from that of caspase‐3 under inflammatory conditions.^[^ ^30^ ^]^ In LPS‐induced inflammasome activation, caspase‐7, rather than caspase‐3, is responsible for PARP1 cleavage, leading to proinflammatory gene expression.^[^ ^32^ ^]^ The current study revealed that active caspase‐7‐enriched migrasomes from RCC cells had no effect on stromal cell death but promoted the expression of proinflammatory genes, such as IL‐6, in macrophages. These results emphasize the essential role of caspase‐7 in inflammation through migracytosis. The proinflammatory cytokine IL‐6 has multiple effects and is associated with poor survival in cancers.^[^ ^33^ ^]^ We also found a negative correlation between IL‐6 expression and RCC survival. Single‐cell RNA sequencing revealed that macrophages were the predominant IL‐6‐expressing cell type in RCC and that active caspase‐7 migracytosis promoted IL‐6 activity in the TME. In summary, ITM2B truncation‐triggered active caspase‐7 migracytosis hyperactivates the IL‐6 pathway in RCC, eventually resulting in a feedback effect that exacerbates RCC progression. Considering that other immune cells also internalize a certain quantity of tumoral migrasomes and that migrasomes can perform functions even without being internalized by recipient cells,^[^ ^12^ ^]^ further exploring the potential effects of tumoral migrasomes on other immune cells is needed.

ITM2B molecules frequently undergo cleavage to perform distinct functions. In neurodegenerative diseases, ITM2B and its truncations play distinct roles in processing amyloid‐beta precursor (APP). Specifically, the ITM2B^1‐243^ truncation protein interacts with APP to inhibit amyloid beta peptide (Aβ) production, the ITM2B^244‐266^ truncation protein prevents Aβ aggregation, and full‐length ITM2B promotes Aβ clearance.^[^ ^15^ ^]^ In fact, ITM2B cleavage also occurs in various tumor cell lines, but the functions of ITM2B truncations remain unknown. In RCC cells, the ITM2B^1‐115^ truncation, but not the full‐length ITM2B or ITM2B^116‐266^ truncation, is transferred to RFs and promotes migrasome formation and migracytosis of active caspase‐7. Compared with para‐carcinoma samples, clinical RCC samples presented lower full‐length ITM2B levels but maintained high ITM2B truncation levels, further supporting the protumoral role of ITM2B truncation. Another intriguing question is whether the decrease in full‐length ITM2B is simply a byproduct of the cleavage of ITM2B to produce ITM2B truncation or plays other roles in regulating RCC progression. This requires further investigation.

MSU crystals, which form during physiological hyperuricemia, were found to increase ITM2B cleavage to produce ITM2B truncation in RCC cells, leading to migrasome formation and active caspase‐7 migracytosis. On the basis of these findings, the combination of potassium oxonate and hypoxanthine to induce hyperuricemia in vivo further verified that hyperuricemia indeed utilizes the ITM2B truncation–migrasome axis to aggravate RCC growth, highlighting the importance of ITM2B truncation‐induced migrasomes under physiological and pathological conditions. We noticed that MSU crystals exerted a promotion of migrasome formation in ITM2B^I115A^‐expressing RCC cells, implying that additional mechanisms are involved in MSU crystal‐induced migrasome formation. Of course, the mechanism underlying MSU crystals facilitates ITM2B cleavage remains elusive, and the protease mediating ITM2B cleavage needs further identification. The hyperuricemia prevalence rate exceeds 20% in both sexes.^[^ ^52^ ^]^ Tumor lysis syndrome during cancer therapy is an emergency in which cancer cells are lysed to release purine nucleotides, leading to hyperuricemia.^[^ ^53^, ^54^ ^]^ Therefore, hyperuricemia frequently occurs in clinical RCC patients. Recently, a prospective cohort study of 444,462 participants revealed an increased risk of kidney cancer among participants with higher serum uric acid levels,^[^ ^38^ ^]^ and a retrospective study of 905 renal cell carcinoma patients revealed that increased serum uric acid levels were negatively associated with patient survival.^[^ ^55^ ^]^ These studies suggest that hyperuricemia is associated with poor RCC survival. Our findings that hyperuricemia is linked with ITM2B truncation‐regulated migrasome formation and that both migrasomes and ITM2B truncation maintain higher levels in the urine of RCC patients are anticipated to provide crucial insights. Specifically, blocking ITM2B truncation may represent a potential therapeutic strategy for managing complications related to RCC. Moreover, ITM2B truncation‐containing migrasomes detected in urine may serve as a noninvasive means to monitor RCC progression.

## 4 Experimental Section

### Antibodies and Reagents

The goat anti‐mouse (#115‐035‐003) and anti‐rabbit (#111‐035‐003) antibodies were purchased from Jackson ImmunoResearch Inc. Anti‐CD63 (#ab271286), anti‐PIGK (#ab201693), anti‐F4/80 (#ab6640) and anti‐Ki67 (#ab16667) antibodies were purchased from Abcam. Anti‐TSG101 antibody (#A5789) and anti‐IL‐6 (#A27935) were purchased from ABclonal. Anti‐mouse IgG Alexa Fluor 488 (#4408), anti‐caspase‐7 (#12827), and anti‐cleaved caspase‐7 (Asp198) (#9491) antibodies were purchased from Cell Signaling Technology. Anti‐phospho‐STAT3 Tyr705 antibody (#AT0604) was purchased from Engibody Biotechnology. Anti‐GFP antibody (#GNI4110‐GP‐S) was purchased from GNI. Anti‐rat IgG Alexa Fluor 594 (#A11007) was purchased from Thermo Fisher Scientific. Anti‐STAT3 antibody (#06‐596) was purchased from Merck Millipore. Anti‐His antibody (#HRP‐66005) and anti‐CP (#21131‐1‐AP) were purchased from Proteintech. Anti‐Flag (#F1804), anti‐HA (#H9658), anti‐CPQ (#HPA023235), and anti‐Tubulin (#T0198) antibodies were purchased from Sigma‐Aldrich. The polyclonal rabbit antibody against ITM2B was generated by immunizing rabbits with prokaryotic human ITM2B peptide (aa 1–60) expressed in *E. coli*.

Phosphatase inhibitor cocktail (#K1012, #K1013) and Z‐VAD (#A1902) were purchased from APExBIO. PMSF (#A610425) and PI (propidium iodide, #A601112) were purchased from BioBasicInc. β‐ME (β‐mercaptoethanol, #60‐24‐2) was purchased from Aladdin. TCEP (T7087) was purchased from TargetMol. DiI (#C1036) was purchased from Beyotime. Anti‐Flag magnetic beads (#B26102) were purchased from Bimake. CF488A WGA (Wheat Germ Agglutinin) (#29022) was purchased from Biotium. Rhodamine‐WGA (#FS0540) was purchased from FUSHENBIO. Ni‐NTA agarose (#HY‐K0210), protease inhibitors cocktail (#HY‐K0010), and protein G‐sepharose (#HY‐K0214) were purchased from MedChemExpress. Proteinase K (#V900887) was purchased from Sigma‐Aldrich. HA magnetic beads (#88836) was purchased from Thermo Fisher Scientific. Recombinant Human Fibronectin (#40113ES03) was purchased from YEASEN. HRP/DAB detection IHC kit (#HC301) was purchased from Vazyme.

### Cell Culture and Transfection

The human RCC cell lines 786‐O (#CL‐0010), ACHN (#CL‐0021), and the mouse RCC cell line Renca (#CL‐0568) were purchased from Procell Life Science&Technology and maintained according to manufacturer's instructions. The human RCC cell line A498 (#HTB‐44), human melanoma cell line SK‐MEL‐1 (#HTB‐67), human cervical carcinoma cell line HeLa (#CRM‐CCL‐2), human monocyte‐like cell line THP‐1 (#TIB‐202), human embryonic kidney cell line 293T (#CRL‐11268) and human acute promyelocytic leukemia cells HL‐60 (#CCL‐240) were obtained from American Type Culture Collection (ATCC). The human melanoma cell line A375 (#TCHu155), human hepatoma cell line Huh7 (#TCHu182), and human non‐small cell lung cancer cell line A549 (#TCHu150) were obtained from the Cell Bank in the Chinese Academy of Sciences. The human lung cancer cell line PC‐9 (#IM‐H125), human breast cancer cell line MDA‐MB‐231 (#IM‐H026), human melanoma cell line MV3 (#IM‐H175), and NK cell line NK‐92 (#IM‐H200) were purchased from Xiamen Immocell Biotechnology Co., Ltd. The human hepatoma cell line HLF (#CTCC‐007‐0154) was obtained from Meisen Cell. Cells were cultured in Dulbecco's modified Eagle's medium (DMEM) (#02‐5062EJ, Gibco) for the SK‐MEL‐1, Hela, 293T, A375, Huh7, A549, ACHN, MDA‐MD‐231, MV3 and HLF cell lines or RPMI‐1640 medium (#P210LV, BasalMedia) for the A498, THP‐1, HL‐60, Renca, 786‐O and PC‐9 cell lines, supplemented with 10% fetal bovine serum (#BC‐SE‐FBS08, Bio‐channel), 100 IU penicillin (#A600135, BioBasicInc) and 100 mg mL^−1^ streptomycin (#A610494, BioBasicInc). Cells were maintained in a humidified incubator equilibrated with 5% CO_2_ at 37 °C. Transfections were performed using ViaFect Transfection Reagent (#E4982, Promega) for 786‐O cells according to the manufacturer's instructions, and the calcium–phosphate precipitation method for 293T cells.

### Plasmid Constructions

The cDNA sequences for ITM2B fused with Flag, HA, His, GFP, mCherry or APEX2 tags were inserted into pLenti vector (Addgene 22255). The cDNA sequences for TSPAN4 fused with HA, His or mCherry tags were inserted into pLenti vector. The cDNA sequences for SMS2 fused with C‐terminal Flag, mCherry or BFP tags were separately inserted into pLenti vector. The cDNA sequences for full length caspase‐7, or p10 and p20 subunit of caspase‐7 fused with C‐terminal Flag tag were inserted into pLenti vector. Single point or deletion mutations in ITM2B or TSPAN4 were constructed using the QuikChange method. All plasmids were verified by sequencing and purified by TIANprep Mini Plasmid Kit (#DP106, Tiangen). The primers that were used to make these plasmids are available upon request.

### Lentivirus Packaging and Infecting

Lentivirus‐based vector pLenti and pLKO.1 were separately used to construct gene overexpression and knockdown lentivirus. pLenti and pLKO.1 vector inserted with target sequence was transfected together with corresponding packaging plasmids into HEK293T cells and cultured for 48 h with adding non‐essential amino acids (NEAA) (#S220JV, BasalMedia) to generate lentivirus. Lentivirus‐containing supernatants were then filtered with 0.45 µm filters and collected. Target cells were infected by corresponding lentivirus for 24 h with adding 10 µg mL^−1^ polybrene (#H9268, Sigma‐Aldrich). The Cells were screened with puromycin (#ant‐pr‐1, InvivoGen) to ensure the infecting efficiencies. The oligonucleotide sequences for the shRNA in pLKO.1 are provided in Supporting Information (Table S1, Supporting Information).

### RNA Extraction and Quantitative Real‐Time PCR

RNA isolater Tatal RNA Extraction Reagent (#R401‐01, Vazyme) was applied to extract total RNA. Subsequently, the gDNA Remover and ABScript Neo RT Master Mix (#RK20433, ABclonal) were applied for reverse transcriptions. Complementary DNAs were used as templates, and *β‐actin* was used as a normalization control. NovoStart SYBR qPCR Supermix Plus (#E096‐01A, Novoprotein) was used to perform quantitative real‐time PCR experiments. The primer sequences are provided in Supporting Information (Table S2, Supporting Information).

### Mouse Models

BALB/c mice were obtained from the Slac Laboratory Animal Center (Shanghai, China) and housed under standard conditions at the Laboratory Animal Center of Xiamen University. All animal experiments were approved by the Animal Ethics Committee of Xiamen University, and all tumor burdens didn't exceed the permitted limits of animal ethics.

The orthotopic renal cancer model was performed as previously described^[^ ^56^ ^]^ with a modification to guarantee successful injection. In brief, an incision on both dermis and peritoneum to expose the left kidney of mouse was first performed, then 1 × 10^6^ luciferase‐expressing Renca cells (resuspended in 50 µL PBS) were injected into the kidney. The needle was hold in kidney for 10 s to reduce the backflow or leak of Renca cells after injecting, and then sutured the incision. Around 25 days after inoculation, the mice were intraperitoneally injected with 3 mg D‐luciferin (#DD1210‐03, Vazyme, dissolved in PBS for 15 mg/mL) for 10 min and imaged with PerkinElmer IVIS Lumina III system to indicate tumor burdens. Then the mice were euthanized for necropsy.

For the subcutaneous model with migrasomes treatment, 1 × 10^6^ Renca cells were resuspended in 100 µL PBS and inoculated into the flank of 8‐week‐old male or female BALB/c mice. Seven days after tumor inoculation, PBS (50 µL) or 5 µg migrasomes (resuspended in 50 µL PBS) were administered peritumorally into the allografts. These injections were repeated every three days until day 18 or 21. Then the mice were euthanized for necropsy.

The hyperuricemia mouse model was conducted with the combination of potassium oxonate (#P137112, Aladdin) and hypoxanthine (#H108384, Aladdin) as previously described.^[^ ^41^ ^]^ Potassium oxonate was dissolved in PBS (warming at 60 °C and ultrasonic) and hypoxanthine was resuspended in saline (warming at 60 °C and ultrasonic) separately. Hyperuricemia mice were administrated with intraperitoneal potassium oxonate (100 mg^−1^kg^−1^day^−1^) and intragastrical hypoxanthine (500 mg ^−1^kg^−1^day^−1^), and the control group mice were administered with an equal volume of PBS and saline. The administration of potassium oxonate and hypoxanthine was carried out from the day after the implantation of orthotopic RCC cells until the mice were euthanized.

### Immunohistochemical Staining and HE Staining

Tumor tissues were fixed with 4% formaldehyde solution. Leica TP1020 Automatic Benchtop Tissue Processor was applied to the dehydration of tissues. Subsequently, tissues were embedded with paraffin. Paraffin‐embedded tissues were sectioned and then deparaffinized with ethanol and xylene. The tissues sections were rehydrated for further immunohistochemical staining. DAB Detection Kit was applied to stain corresponding proteins according to manufacturer's instructions.

### Gomori's Methenamine Silver Stain for observation of Urate Crystals

Urate stain kit (GMS method, G3030, SolarBio) was employed to indicate the MSU crystal deposited in RCC cells and tissues. RCC cells were fixed in absolute ethanol for 1 h, the staining procedure was performed according to manufacturer's instructions. Tissues were fixed in absolute ethanol for 18 h and then embedded in paraffin. The sections were stained according to manufacturer's instructions.

### Imaging

For confocal imaging, all migrasome observations were performed using living cell imaging since the integrity of retraction fibers and migrasomes was susceptible to the process of fixing cells. Cells were seeded in glass‐bottom dishes precoated with 4 µg mL^−1^ fibronectin for 18–24 h. If necessary, WGA (2 µg mL^−1^) was introduced into culture mediums 10 min before imaging. The images were collected by IXplore SpinSR (Olympus). For other subcellular localization observation, cells were first fixed and labeled with corresponding antibodies, then the images were collected by IXplore SpinSR (Olympus).

SIM‐Ultimate (CSR Biotech) was applied for super‐resolution imaging and TIRF (total internal reflection fluorescence) imaging. Imaging was processed with the corresponding elements of SIM‐Ultimate.

### Electron Microscopy

For morphological analysis of purified migrasomes, purified migrasomes were placed onto the carbon/formvar‐coated grids and underwent negative staining with 1% uranyl acetate. The sample was observed under an electron microscope (JEM‐2100HC (JEOL)).

To analyze APEX2‐fused constructions, in situ transmission electron microscopy was applied to observe the bottom layers of cells because retraction fibers and migrasoems are located in these layers. APEX2‐fused constructions were stably expressed in 786‐O cells and seeded the cells in 35 mm culture dishes for 24 h. Cells were fixed in the cultured dishes with 2.5% glutaraldehyde and then stained with DAB and H_2_O_2_. The samples were then reacted with OsO_4_ to produce EM contrast. After reaction, samples were dehydrated and embedded with resin. The bottom layers were sectioned for observation with JEM‐2100HC (JEOL).

### Isolation of Cell Body and Migrasome

Isolation of the migrasome was performed as previously described^[^ ^11^ ^]^ with modifications based on the type of centrifuges. Cells were seeded in 150 mm dishes precoated with 4 µg mL^−1^ fibronectin for 36 h. Removed the supernatants of cultured cells and the cells were washed with PBS. Trypsin (0.25%, 3 mL) was used to detach cells and migrasomes, FBS‐containing culture mediums (10%, 3 mL) were added to stop digestion. The resuspensions were collected and centrifuged at 1000 g for 10 min to pellet the cell bodies. The collected supernatants were centrifuged at 4000 g for 20 min, and then recentrifuged at 20 000 g for another 30 min to obtain crude migrasomes. Crude migrasomes were performed with Optiprep density gradient centrifugation to get purified migrasomes. First, crude migrasomes were resuspended in 1 × extraction buffer (400 µL) and then mixed with Optiprep (10%, 400 µL). Second, Optiprep density gradient was built with crude migrasomes (5%, 800 µL), 10% (400 µL), 15% (400 µL), 20% (400 µL), 25% (400 µL), 30% (400 µL), 35% (400 µL), 40% (400 µL) and 50% (400 µL) in 4 mL centrifuge tubes. Then, the prepared tubes were centrifuged at 15 00 00 g for 2 h at 4 °C in SW 60 Ti Swinging‐Bucket rotor (335649, Beckman Coulter). Lastly, collected 15%, 20% and 25% Optiprep fractions (where migrasomes were enriched) and each fraction was mixed with double volume (800 µL) PBS and centrifuged at 20,000 g for 30 min to get purified migrasomes. Then migrasomes were resuspended in the appropriate buffer for the following expriments.

For the isolation of migrasome from human urine, 80–120 mL of early morning urine from normal or RCC patients was collected and performed as described above. Purified migrasomes were dissolved in PMSF‐containing lysis buffer to measure the concentrations of migrasome proteins.

### Isolation of ITM2B‐containing Vesicles

ITM2B‐containing vesicles were isolated based on an immune purification strategy to isolate organelles as previously described,^[^ ^57^ ^]^ with some modifications. Since the N‐terminal of ITM2B is located on the outer membrane of vesicles (a typic characteristic of type II transmembrane protein), the HA or Flag tag was coupled to the N‐terminal of ITM2B and utilized the tags of ITM2B (exposure on the outer of vesicles) as handles for immunocapture. First, HA‐ or Flag‐tagged ITM2B and ITM2B mutants were stably expressed in 786‐O cells and 4 × 10^7^ cells were prepared for vesicle isolation. Cultured cells were rapidly washed once with KPBS (136 mm KCl, 10 mm KH_2_PO_4_, pH 7.25) and then scraped in KPBS. Cells were centrifuged at 1000 g for 2 min and then resuspended cell pellets in 1 mL KPBS. Cell suspensions were homogenized for 50 times in a 2 mL complete KIMBLE Dounce tissue grinder set (#D8938, Sigma) to release cell contents (including cytoplasm and organelles). The homogenized suspensions were centrifuged at 1000 g for 5 min twice to remove cells and large cell debris. The collected supernatants were further centrifuged at 10 00 00 for 40 min to precipitate organelles. The pellets in KPBS were resuspended and HA‐ or Flag‐tag magnetic beads were applied to capture ITM2B‐containing vesicles.

### Incubation of Macrophage with Tumor Migrasome

THP‐1 cells (1 × 10^6^) were seed in 6‐well plates. 100 ng mL^−1^ PMA (#TQ0198, TargetMol) were added and incubated for 24 h to induce the differentiation of THP‐1 cells into macrophages. Migrasomes derived from 786‐O cells were purified, and 10 µg migrasomes (≈9.5 × 10^7^ particles) were used to incubate a well of macrophages for 24 h. Then, macrophages were prepared for Western blots. For the detection of IL‐6 secretion, incubated macrophages were washed with serum‐free medium for twice, and fresh serum‐free medium was added to the culture cells for another 12 h. The culture medium was collected to measure IL‐6 concentration by Human IL‐6 ELISA Kit (#RK00004, ABclonal), according to the manufacturer's directions.

Mouse macrophages (BMDMs) were differentiated from bone marrow cells of BALB/c mice with 20 ng mL^−1^ M‐CSF (#CB34, Novoprotein)‐supplemented media for 7 days. Migrasomes derived from Renca cells were purified, and 10 µg migrasomes (≈1.35 × 10^8^ particles) were used to incubated with mouse macrophages. IL‐6 secretion from these cells was measured by Mouse IL‐6 ELISA Kit (#RK00008, ABclonal).

### Immunoprecipitation

Cells were lysed with protease inhibitor cocktail‐ and phosphatase inhibitor cocktails‐containing lysis buffer (20 mm Tris, 150 mm NaCl, 1 mm EDTA, 1 mm EGTA, 2.5 mm sodium pyrophosphate, 1% Triton X‐100) on ice. A small amount of cell lysates was used to prepare WCL (whole cell lysate) and remaining cell lysates were incubated with protein G‐sepharose beads and corresponding antibodies for 3 h in 4 °C. The beads were subsequently precipitated with a centrifugation at 3000 rpm for 1 min and washed three times with PMSF‐containing lysis buffer.

### His Pull‐Down Assay

Cells were lysed with a protease inhibitors cocktail and 1% Triton X‐100‐containing lysis buffer (50 mm NaH_2_PO_4_, 300 mm NaCl, 10 mm imidazole, pH 8.0) on ice. A small amount of cell lysates was used to prepare WCL, and remaining cell lysates were incubated with Ni‐NTA agarose for 90 min in 4 °C. The Ni‐NTA agarose was washed for three times (10 min per times) with 8 m urea‐containing lysis buffer (50 mm NaH_2_PO_4_, 300 mm NaCl, 50 mm imidazole, pH 8.0) to remove non‐covalent protein bindings. Then elution buffer (50 mm NaH_2_PO_4_, 300 mm NaCl, 500 mm imidazole, pH 8.0) was used to eluate his‐tagged proteins from Ni‐NTA agarose and prepared the elution buffers for subsequent western blot analysis.

### Measurement of Uric Acid Concentration of Mice Serum

The concentrations of uric acid in mice serum were measured by the Uric Acid (UA) Content Assay Kit (#D799285, Sangon Biotech) according to the manufacturer's directions.

### Observation of Migrasome from Mouse Urine

The observation of migrasome from mouse urine refers to the previous report^[^ ^58^ ^]^ with some modifications. Briefly, fibronectin (20 µg/mL) was used to precoated dish for 2 h. Mouse urine was centrifuged at 3000 g for 5 min, and the supernatants were collected. Then 10 µL urine was added on a 0.5 mm diameter circle and incubated for 2 h to allow migrasomes adhering to the fibronectin‐precoated bottom. The urine was removed and washed with PBS twice. WGA‐containing PBS was used to stain the adhered urine migrasomes for 10 min and imaged under a confocal microscope.

### Mass Spectrometry

Migrasomes and ITM2B‐containing vesicles were isolated and 1% sodium deoxycholate solution was used to dissolve migrasomes and vesicles. A total of 30 µg migrasome protein solution per group were prepared for label‐free mass spectrometric detection and 786‐O cells (4 × 10^7^) were prepared to isolate ITM2B‐containing vesicles for proteomic identification. Proteins were analyzed by timsTOF Pro (Bruker). The data were analyzed by Peaks Studio X software (Bioinformatics Solutions Inc.) and searched against the human UniProt Reference Proteome with isoform.

### Ethics Approval Statement

All animal experiments were approved by the Animal Ethics Committee of Xiamen University (approval No. XMULAC20230326). Clinical RCC samples were obtained from Zhongshan Hospital, Xiamen University, with patient informed consent and the approval of the Medical Ethical Committee of Zhongshan Hospital (approval No. xmzsyyky2024‐136).

### Statistical Analysis

Statistical analyses were performed in GraphPad Prism 9. The statistical analyses between two groups were performed with two‐tailed Student's *t*‐test. The statistical analyses among multiple groups were performed with one‐way or two‐way ANOVA, followed by Tukey's or Sidak's multiple comparison test. Data were presented as the Mean with SEM. Statistical significance was indicated as follows: ^*^ *p *< 0.05, ^**^ *p *< 0.01, ^***^ *p *< 0.001.

## Conflict of Interest

The authors declare no conflict of interest.

## Author Contributions

Q.‐t.C., Q.‐l.H., M.‐z.H., and X.‐h.H. contributed equally to this work. Correspondence should be addressed to Q.W. and H.‐z.C. Q.W. and H.‐z.C. conceived this study, including generating the hypothesis, wrote and reviewed the manuscript. Q.‐t.C., Q.‐l.H., M.‐z.H., Y.C., and Y.‐y.H. performed biological experiments and mouse models. X.‐h.H. collected and analyzed clinical samples. D.‐y.F. performed bioinformatic analyses. L.‐m.Y. provided support for the electron microscope. W.‐b.H. analyzed protein structure.

## Supporting information



Supporting Information

Supplemental Data

Supplemental Movie 1

Supplemental Movie 2

Supplemental Movie 3

Supplemental Movie 4

Supplemental Movie 5

Supplemental Movie 6

Supplemental Movie 7

Supplemental Movie 8

Supplemental Movie 9

Supplemental Movie 10

Supplemental Movie 11

## Data Availability

The mass spectrometry proteomics data have been submitted to ProteomeXchange via the PRIDE database with identifier PXD054746 and PXD068814. All materials and reagents are available from the corresponding author upon reasonable request.
