# Supplementary material for: ITM2B Truncation Promotes Migrasome Formation to Accelerate Renal Cell Carcinoma Growth
Source: Adv Sci (Weinh). 2025 Nov 30;13(4):e11683. doi: 10.1002/advs.202511683 (PMC12822405; doi:10.1002/advs.202511683)
Supplement: Supplementary file 1 — Supporting Information [file ADVS-13-e11683-s009.pdf]

Supporting Information

ITM2B Truncation Promotes Migrasome Formation to Accelerate Renal Cell Carcinoma Growth

Qi-tao Chen, Qiao-ling Huang, Ming-zhi Han, Xue-hui Hong, De-yi Feng, Lu-ming Yao, Wen-bin Hong, Yue Chen, Ya-ying Huang, Hang-zi Chen, Qiao Wu**

Q. Chen, Q. Huang, M. Han, D. Feng, L. Yao, W. Hong, Y. Chen, Y. Huang, H. Chen, Q. Wu, State Key Laboratory of Cellular Stress Biology, School of Life Sciences, Xiamen University, Xiamen, Fujian 361102, China

X. Hong and Q. Wu

Department of Gastrointestinal Surgery, Zhongshan Hospital of Xiamen University, School of Medicine, Xiamen University, Xiamen, Fujian 361004, China

Equal contribution: Qi-tao Chen, Qiao-ling Huang, Ming-zhi Han and Xue-hui Hong contribute equally to this work.

Correspondence: qiaow@xmu.edu.cn (Qiao Wu) and chenhz@xmu.edu.cn (Hang-zi Chen).

Supplemental figures

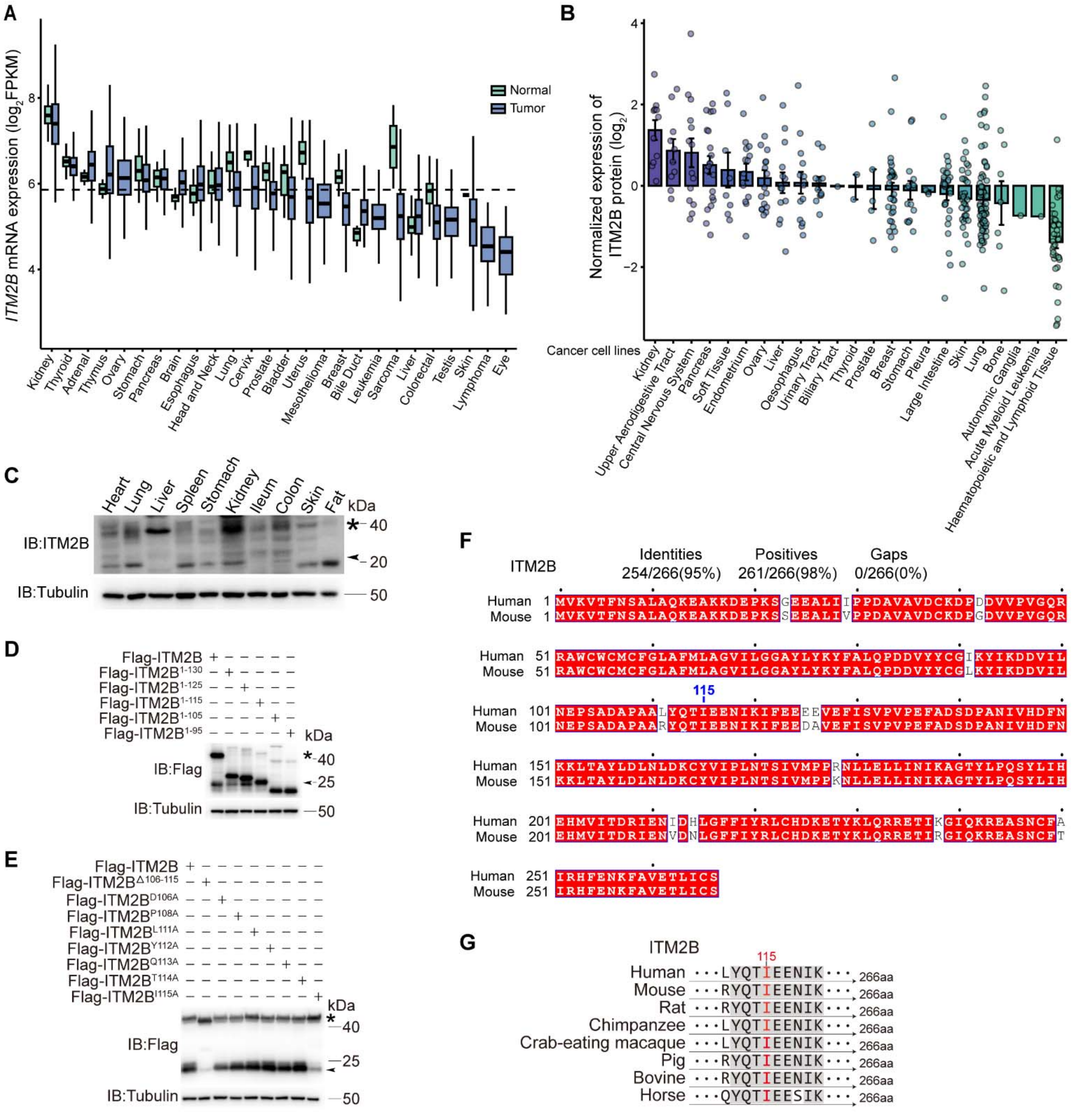

Figure S1 Site I115 of ITM2B is Crucial for Its Cleavage.

(A) Analysis of *ITM2B* mRNA expression levels in different normal tissues and their corresponding tumors. Source data is obtained from TCGA. **(B)** Analysis of ITM2B protein expression levels in different cancer cell lines. Source data is obtained from CCLE. **(C)**

Detection of ITM2B protein in indicated mouse tissues. **(D)** Expression pattern of ITM2B and its fragments in 786-O cells. **(E)** Expression pattern of ITM2B, ITM2B deletion and ITM2B point mutants in 786-O cells. **(F)** The comparison of amino acid sequence homology between human and mouse ITM2B proteins. **(G)** The amino acid sequences around site 115 of ITM2B in different species.

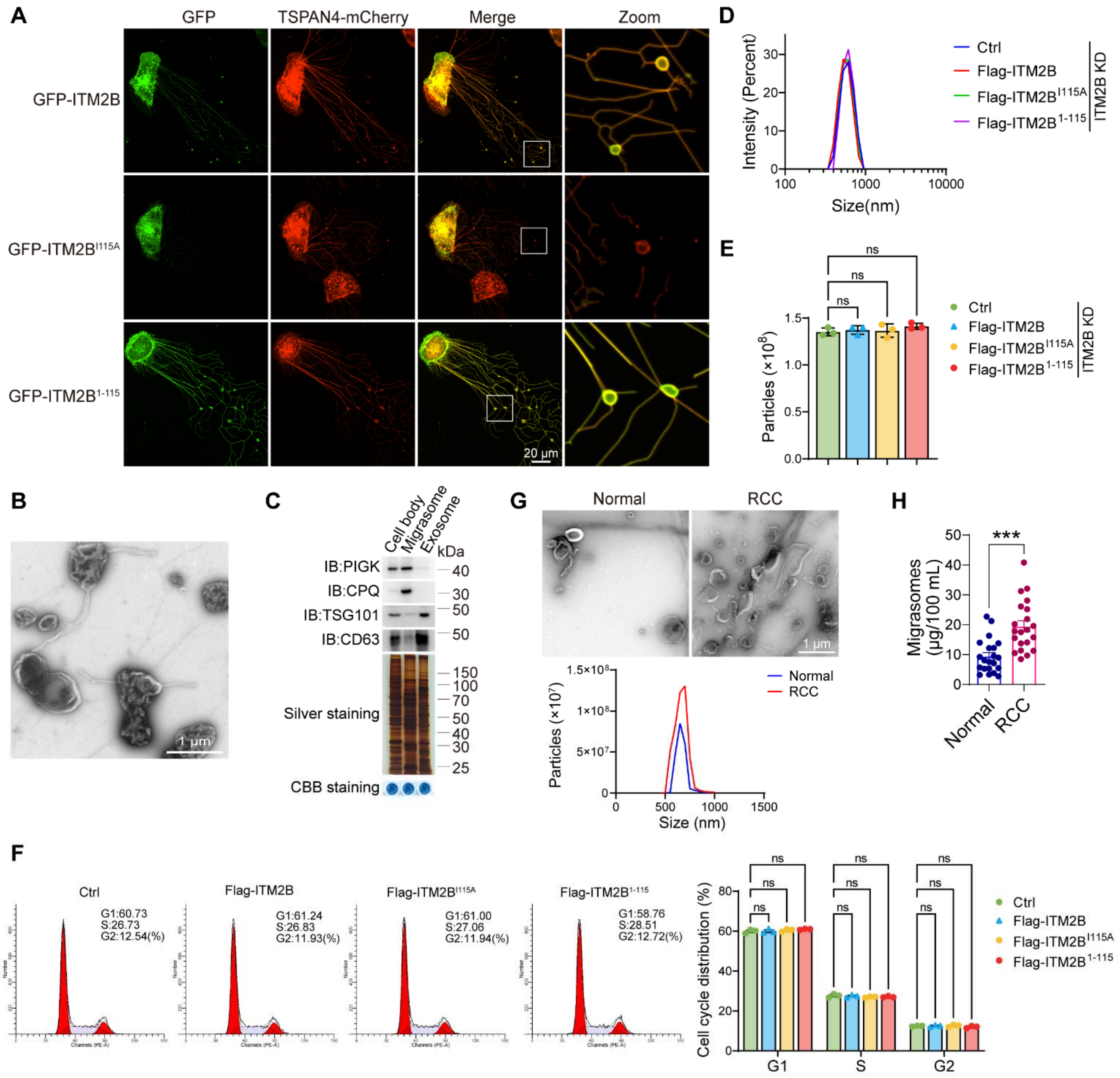

Figure S2 RCC Patients Show Higher Levels of Uric Migrasomes.

(A) TSPAN4-mCherry and GFP-ITM2B or its mutants were transfected into 786-O cells. Live-cell imaging was applied to show these cells. **(B)** A transmission electron microscopy was applied to visualize purified migrasomes from 786-O cells. **(C)** Cell body, migrasome and exosome were prepared in 786-O cells. PIGK and CPQ were utilized as migrasome markers, while TSG101 and CD63 were employed as exosome markers. Migrasome and exosome

makers were indicated in equal protein amounts. Coomassie brilliant blue (CBB) staining was used for equal protein quantification and silver staining was used for equal loading validation. **(D-E)** Migrasomes were purified from indicated Renca cells. Dynamic light scattering (DLS) was applied to measure the sizes of migrasomes (D). Nanoflow cytometry was applied to count the migrasome numbers in 10 μg purified migrasomes (E). **(F)** Cell cycle was analyzed by flow cytometry with propidium iodide (PI) staining. **(G-H)** Migrasomes were purified from early morning urine (100 mL) of normal volunteer (n=21) and RCC patients (n=21), uric migrasomes were then characterized by electron microscopy (G, top) and nanoflow cytometry (G, bottom). Protein quantification were performed to indicate the amounts of uric migrasome (H). Data were shown as the mean ± s.e.m. *P < 0.05, **P < 0.01, ***P < 0.001.

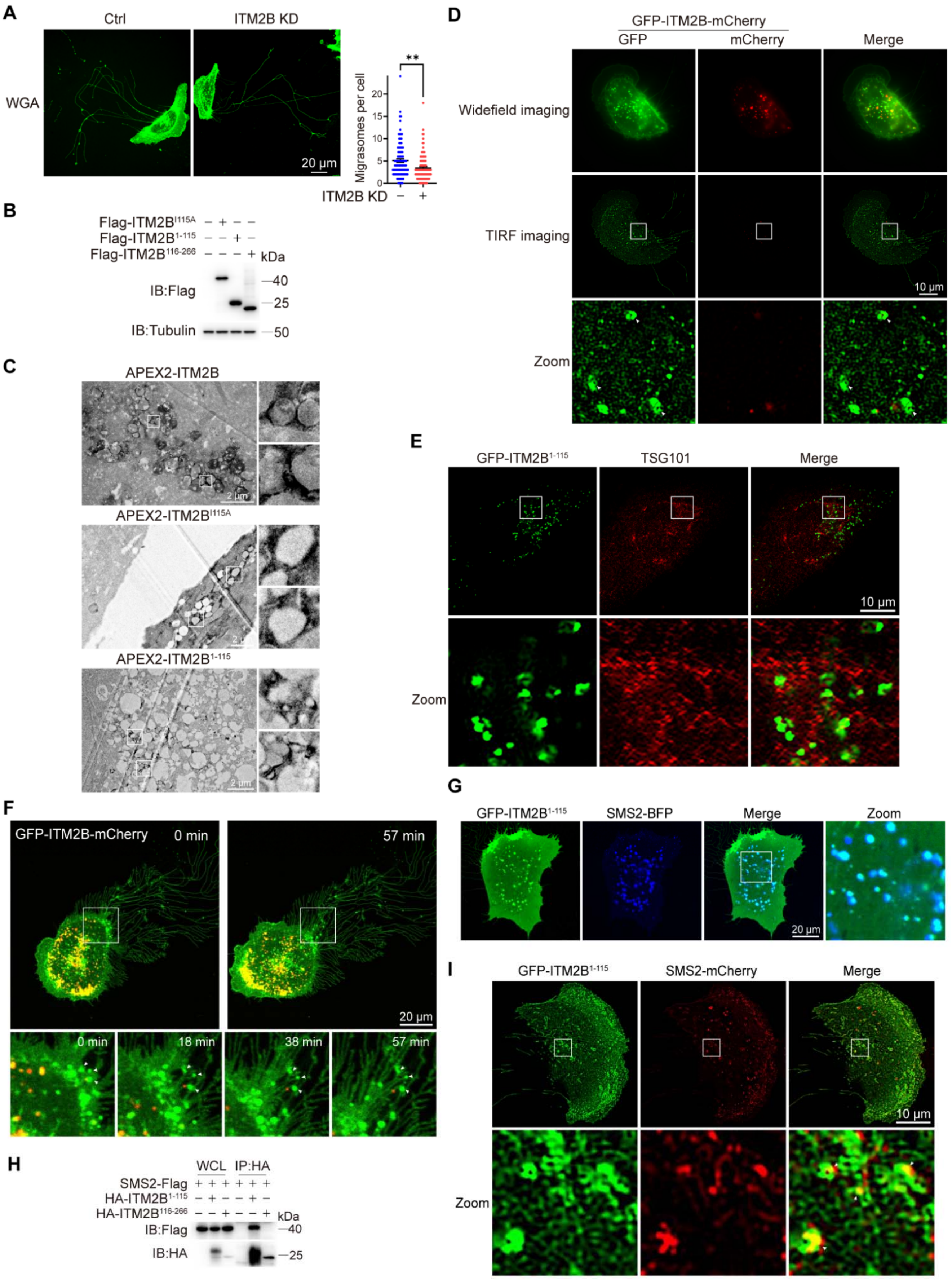

Figure S3 ITM2B Truncation Transfers to SMS2 Foci at Basal Membrane of Cells.

(A) ITM2B-knockdown 786-O cells and the control cells were stained with WGA to show migrasomes (left), and the migrasome numbers within 100 cells were counted in each group (right). **(B)** Overexpression of ITM2B^{I115A}, ITM2B$^{1\text{-}115}$ and ITM2B$^{116\text{-}266}$ in 786-O cells, **(C)** APEX2-ITM2B, APEX2-ITM2B^{I115A} and APEX2-ITM2B$^{1\text{-}115}$ were expressed in 786-O cells, and the APEX2 signals in cell body were visualized using a transmission electron microscope. **(D)** Widefield imaging and TIRF imaging were applied to visualize the same GFP-ITM2B-mCherry-expressing 786-O cells. **(E)** TIRF imaging indicated the location of GFP- ITM2B$^{1\text{-}115}$ and TSG101 in 786-O cells. **(F)** Time-lapse imaging was applied to observe GFP-ITM2B-mCherry-expressing 786-O cells. **(G)** SMS2-BFP and GFP-ITM2B$^{1\text{-}115}$ was expressed in 786-O cells, the cells were fixed and observed. **(H)** ITM2B fragments as indicated and SMS2 were expressed in 786-O cells, their interactions were detected. **(I)** GFP-ITM2B$^{1\text{-}115}$ and SMS2-mCherry were expressed in 786-O cells. TIRF imaging was applied to visualize the cells.

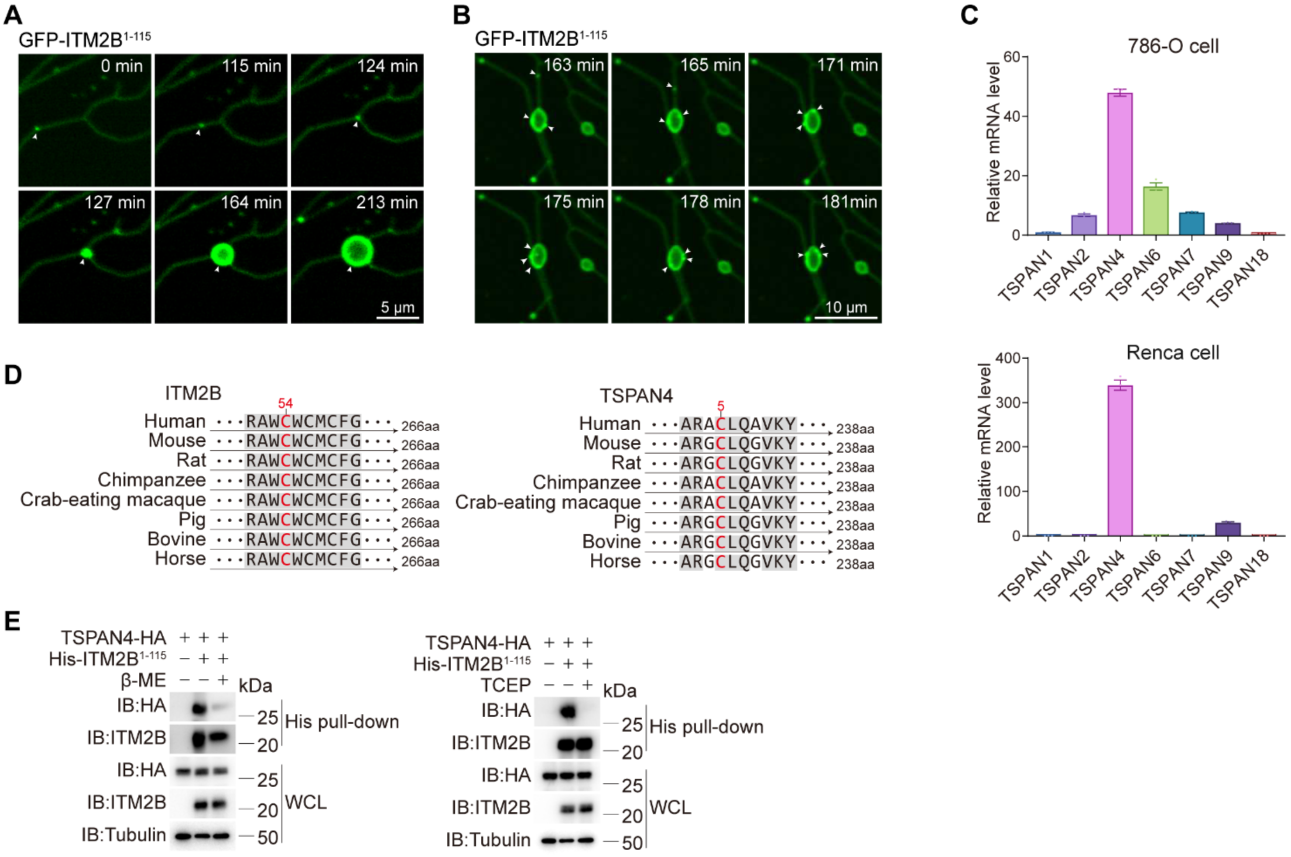

Figure S4 Both Cys54 of ITM2B and Cys5 of TSPAN4 Are Conserved.

(A-B) Time-lapse imaging of ITM2B truncation moving towards migrasomes (A) and facilitating migrasome swelling (B). **(C)** The mRNA expression levels of different tetraspanins in 786-O cells (top) and Renca cells (bottom). **(D)** The amino acid sequences around site 54 of ITM2B (left) and site 5 of TSPAN4 (right) in different species. **(E)** TSPAN4 and ITM2B$^{1\text{-}115}$ were expressed in 786-O cells, and His pull-down assay was performed to indicate their interaction. Ni-NTA agarose beads were incubated with β-ME (100 mM) and TCEP (5 mM) for 10 min before washing with 8 M urea-containing buffer.

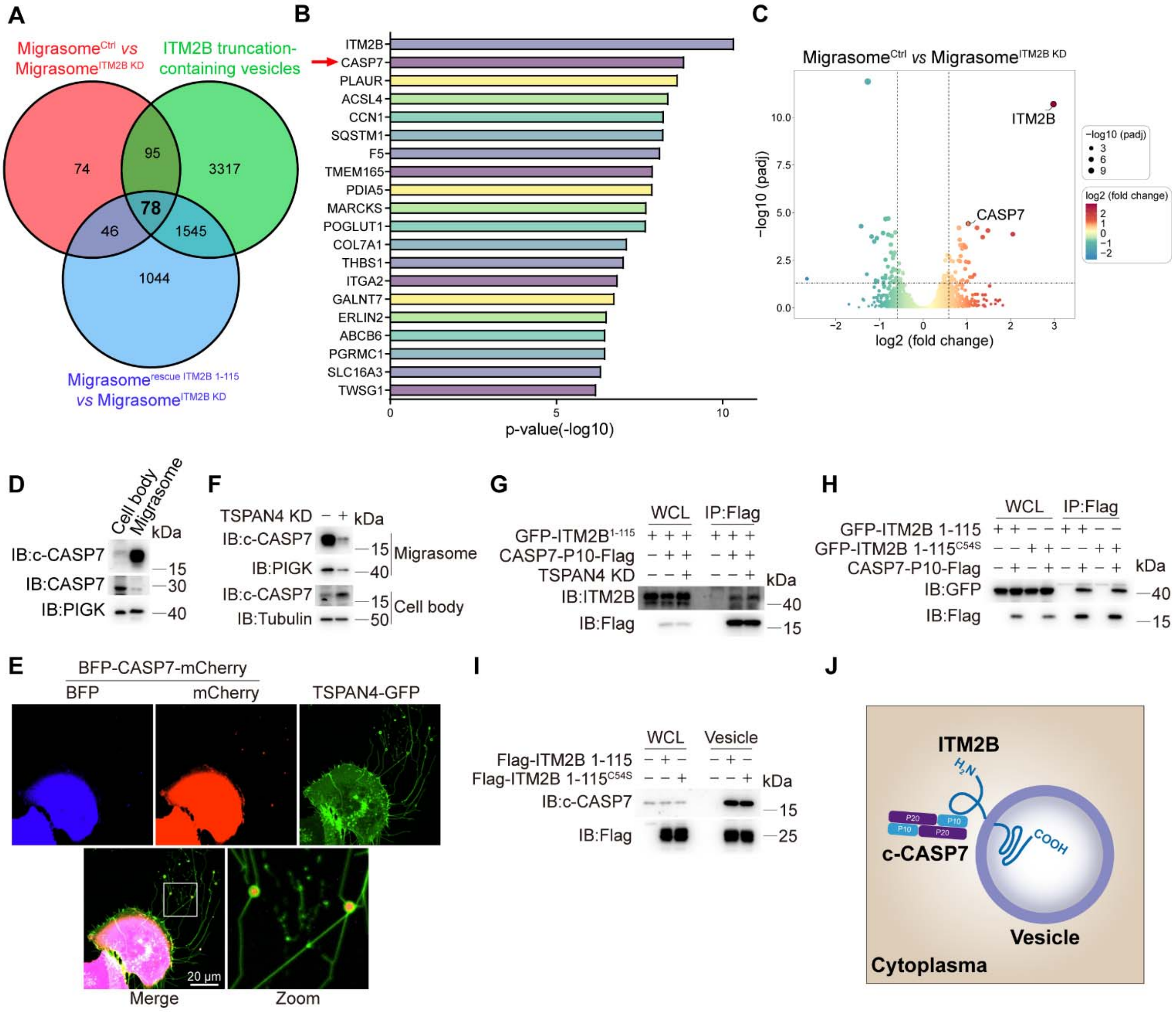

Figure S5 ITM2B Truncation Interacts with Active Caspase-7.

(A) Quantitative proteomics was conducted not only to identify the decreased migrasome substrates (foldchange > 1.5) by comparing ITM2B-knockdown 786-O cells to control 786-O cells (red circle), but also to identify the increased migrasome substrates (foldchange > 1.5) by comparing the re-introducing ITM2B$^{1\text{-}115}$ 786-O cells to ITM2B knockdown 786-O cells (blue circle). ITM2B$^{1\text{-}115}$-containing intracellular vesicles were isolated and analyzed by mass spectrometry (green circle). The overlapping proteins are shown with the form of the Venn diagram. **(B)** Top 20 proteins among the 78 overlapping proteins are listed. **(C)** The change of

proteins between control migrasomes and ITM2B knockdown migrasomes was shown by the volvano plot. **(D)** Cell body and migrasome of Renca cells were collected. The levels of CASP7 and c-CASP7 were detected. **(E)** BFP-CASP7-mCherry and TSPAN4-GFP were expressed in 786-O cells, and live-cell imaging showed the different statuses of caspase-7 in the cell body and migrasomes. **(F)** The cell body and migrasome from equal amounts of TSPAN4-knockdown 786-O cells were collected, the indicated proteins were detected. **(G)** ITM2B$^{1\text{-}115}$ and p10 subunit of caspase-7 were expressed in TSPAN4-knockdown 786-O cells, their interaction was detected. **(H)** ITM2B truncation or its mutant and p10 subunit of CASP7 were expressed in 786-O cells, their interaction was detected. **(I)** Flag-tagged ITM2B truncation and its mutant were expressed in 786-O cells, anti-Flag magnetic beads was used to immunoprecipitate corresponding vesicles. **(J)** Schematic diagram of active caspase-7 tethered to the outer surface of ITM2B truncation-containing vesicles.

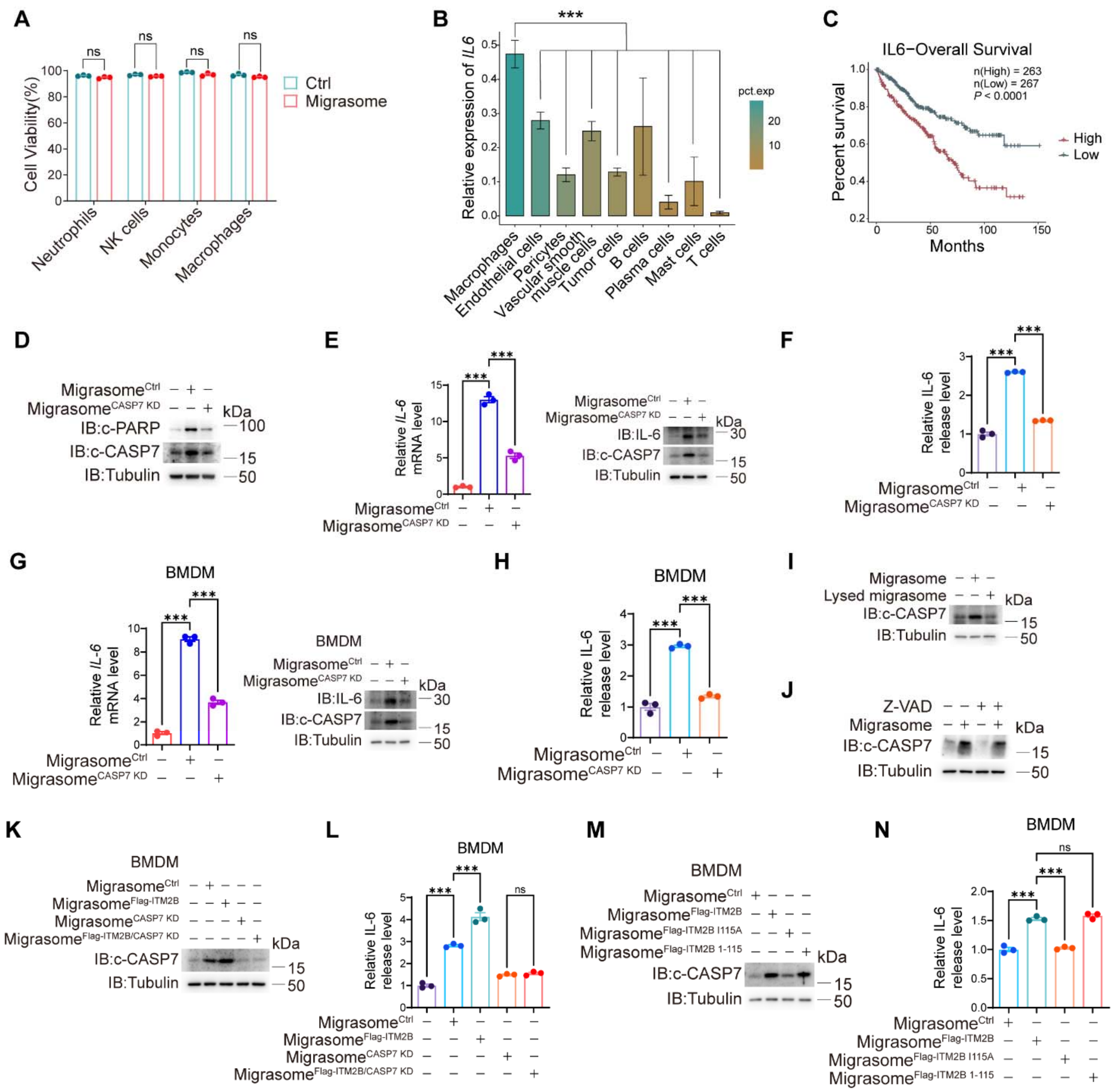

Figure S6 IL-6 Secretion from Macrophages is Correlated with Poor Survivals of RCC.

(A) Various stroma cells were incubated with migrasomes derived from 786-O cells for 24 hours, and cell death was measured. **(B)** Analysis of *IL-6* mRNA expression levels with scRNA-seq (GSE159115) of human RCC. **(C)** RCC data from TCGA were used to analyze the correlation between IL-6 gene expression and RCC survival (n = 530 patients). **(D-F)** THP-1 cells were pretreated with PMA for 24 hours to differentiate into macrophages. The cells were

incubated with migrasomes derived from 786-O cells and CASP7-knockdown 786-O cells for 24 hours. The levels of c-CASP7 and c-PARP1 were detected (D), corresponding IL-6 mRNA and protein expression (E) and IL-6 secretion (F) were detected (n = 3). **(G-H)** Mouse BMDM cells were incubated with migrasomes derived from Renca cells. IL-6 expression (G) and IL-6 secretion (H) in BMDM cells were detected (n = 3). **(I)** Migrasomes form 786-O cells were purified. THP-1 cells-differentiated macrophages were incubated with either intact migrasomes or migrasomes lysed by ultrasound. The levels of active caspase-7 was detected. **(J)** THP-1 cells-differentiated macrophages were incubated with 786-O cells-derived migrasomes or pan caspase inhibitor Z-VAD (20 μM) for 24 hours, the level of active caspase-7 was detected. **(K-N)** Mouse BMDM cells were incubated with indicated migrasomes derived from Renca cells. The expression of active caspase-7 in BMDM cells (K and M) and IL-6 secretion from these cells (L and N) were detected (n=3). Data were shown as the mean ± s.e.m. $^{*}P < 0.05$, $^{**}P < 0.01$, $^{***}P < 0.001$.

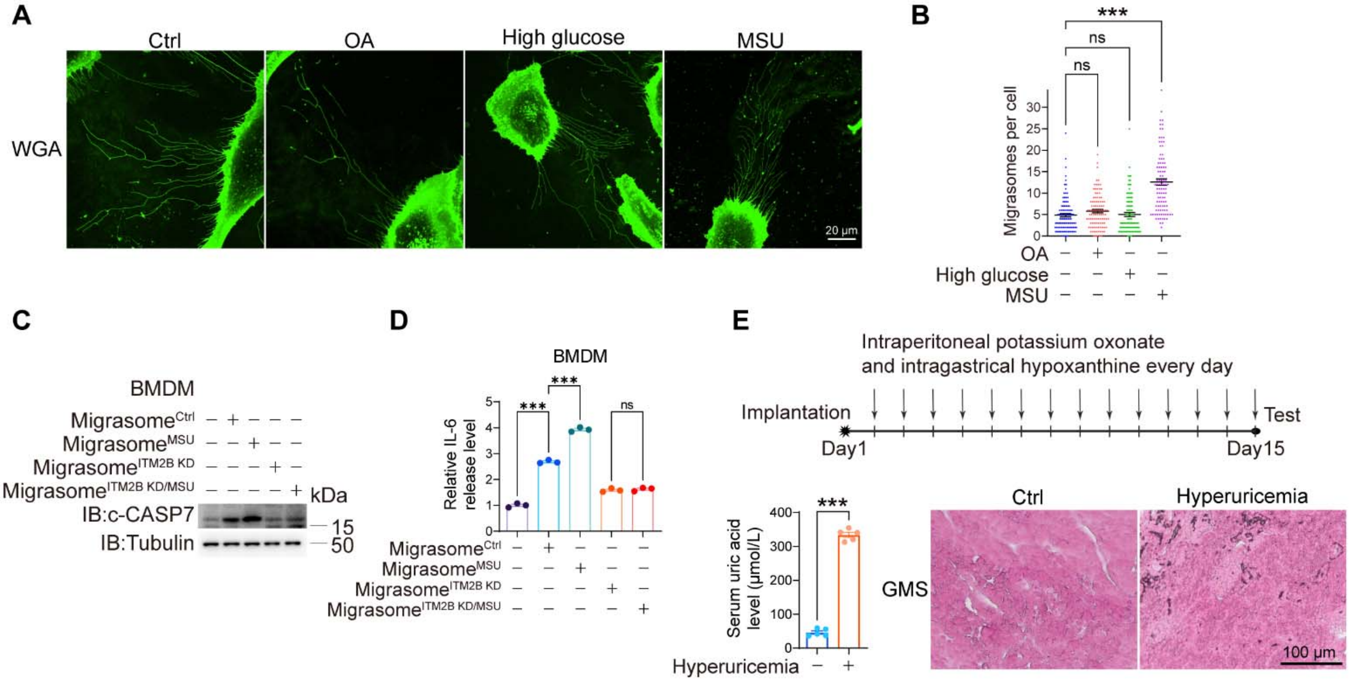

Figure S7 MSU Crystal Induces Migrasome Formation.

(A) 786-O cells were treated with OA (500 μM), high glucose (25 mM) and MSU crystal, and cells were stained with WGA. **(B)** The migrasome numbers with 100 cells were counted in each group. **(C-D)** Mouse BMDM cells were incubated with migrasomes derived from indicated Renca cells. The expression levels of c-CASP7 in BMDM cells (C) and IL-6 secretion from BMDM cells (D) were detected (n=3). **(E)** Experimental scheme was indicated (top). The serum uric acid levels and MSU crystal deposition in tumor tissues were indicated (bottom). Data were shown as the mean ± s.e.m. *$P < 0.05$, **$P < 0.01$, ***$P < 0.001$.

Table S1 Corresponding Oligonucleotide Sequences for the shRNA-targeted mRNA (5'-3')

pLKO.1 human Ctrl shRNA	CAACAAGATGAAGAGCACCAA
pLKO.1 human ITM2B shRNA	GGTGTTGGTGCATGTGCTTTG
pLKO.1 human DGAT2 shRNA	GCTGTGCTCTACTTCACTT
pLKO.1 human gp78 shRNA	GGAATGCACACCTTGGCTT
pLKO.1 human CAV1 shRNA	CCACCTTCACTGTGACGAAAT
pLKO.1 human NPC1 shRNA	ATGTCTTCACCGTTGTAAT
pLKO.1 human ADAM10 shRNA	GGGTCTGTTATTGATGGAAGA
pLKO.1 human RHBDL1 shRNA	CCTCACCTACATGTTCATGCA
pLKO.1 human RHBDL2 shRNA	GGGCTTCTTAATGTAGAAA
pLKO.1 human RHBDL3 shRNA	GCTTGAAGAACTCCCTGGTTT
pLKO.1 human RHBDL4 shRNA	GTACTTACTGGAGTGGTAT
pLKO.1 human PARL shRNA	TCGCAGGTTTAACTTCTTTATT
pLKO.1 human TAPAN4 shRNA	GACCTTCGCCATGACCATG
pLKO.1 human Caspase-7 shRNA	GCTTCTGAAGAGGACCATA
pLKO.1 mouse ITM2B shRNA	GGACAAGTGCTACGTGATT
pLKO.1 mouse DGAT2 shRNA	GGTCATCTCAGTACTACAA
pLKO.1 mouse RHBDL2 shRNA	TGCATTGATGGGAGGATATTT
pLKO.1 mouse TSPAN4 shRNA	GCATCATCCAGACTGATTT
pLKO.1 mouse Caspase-7 shRNA	GCCGCCGTTCGTTGATGAA

Table S2 Corresponding Primer Sequences for Quantitative Real-time PCR (5'-3')

Human β-actin	forward	CAGCCTTCCTTCCTGGGCATG
	reverse	ATTGTGCTGGGTGCCAGGGCAG
Human TSPAN1	forward	CATGCAGTTTGTCAACGTGGG
	reverse	CACTTGCTCTCAGTCTTAGCAC
Human TSPAN2	forward	ATCGGCCGTCATTGCTTTTG

	reverse	CAAGCACACATTGCGACTCC
Human TSPAN4	forward	GCTGTGGCGTCTCCAACTAC
	reverse	CTTGGCAGTACATGGTCATGG
Human TSPAN6	forward	CGCTGCCATCGTAGGATTTG
	reverse	GGTGACACCACAACAATGCAA
Human TSPAN7	forward	ACCAAACCTGTGATAACCTGTCT
	reverse	AGGGAGATATAGGTGCCCAGA
HumanTSPAN9	forward	GTTTGTCAGCAGCCAAGGTG
	reverse	CAAAGTTGCCTTGGGACACG
Human TSPAN18	forward	GCTGTTACACGGTGATCCTCA
	reverse	CATGGCGAAAAGCTCGATGG
Human IL-6	forward	GGCACTGGCAGAAAACAACC
	reverse	GCAAGTCTCCTCATTGAATCC
Mouse β-actin	forward	GATGGCCACTGCCGCATCCTC
	reverse	GGTCTTTACGGATGTCAACGTCAC
Mouse ITM2B	forward	GAAGGTGACGTTCAACTCGG
	reverse	CTCTCCTCTGTCCAACCGGA
Mouse TSPAN1	forward	GAATCTGGGTGTCCGTCGATG
	reverse	AGTAGCCCACGTTGACAAACT
Mouse TSPAN2	forward	ACCGTAATCAGTGCTAAACTGC
	reverse	GTTCCGTATTGCACAGCAAAG
Mouse TSPAN4	forward	TACCTCATGTTCGCCTTCAAC
	reverse	GATAAGGTGGCAAAGTTTCCCT
Mouse TSPAN6	forward	ATCACTGGTGTTATCCTTCTTGC
	reverse	ACAGGTAGCGAAACAACCAAAG
Mouse TSPAN7	forward	ATGGCATCGAGGAGAATGGAG
	reverse	TGAGCACATAGGGAGCATTTG
Mouse TSPAN9	forward	CTGTGGTTTGATGACAACAAGC
	reverse	TTTTTACCTGTCCGGTGGATG

Mouse TSPAN18	forward	GGTGCCTACATCGTACTGGC
	reverse	TGCTTGGTAAGCTCCTTGGTG
