# Supplementary material for: ITM2B Truncation Promotes Migrasome Formation to Accelerate Renal Cell Carcinoma Growth
Source: Adv Sci (Weinh). 2025 Nov 30;13(4):e11683. doi: 10.1002/advs.202511683 (PMC12822405; doi:10.1002/advs.202511683)
Supplement: Supplementary file 2 — Supplemental Data [file ADVS-13-e11683-s008.zip › Supplementary file for uncropped blots.pdf]

Uncropped western blots

Figure 1B

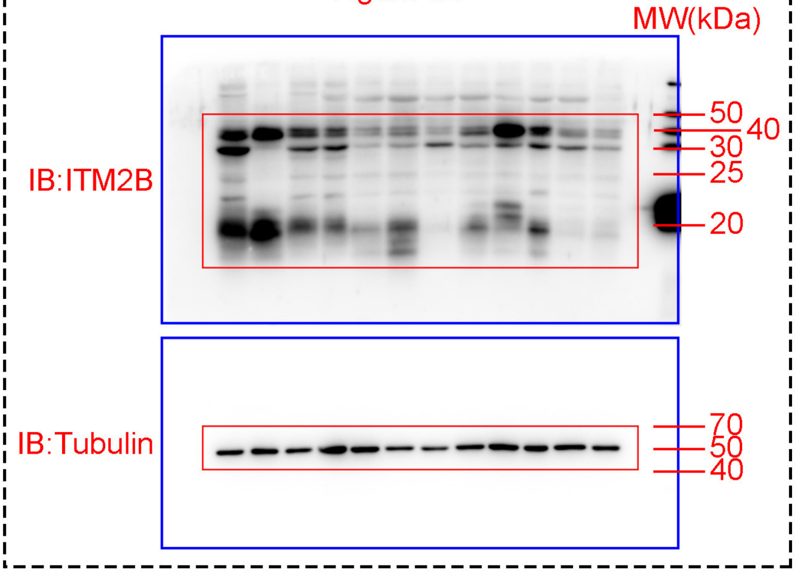

Figure 1C

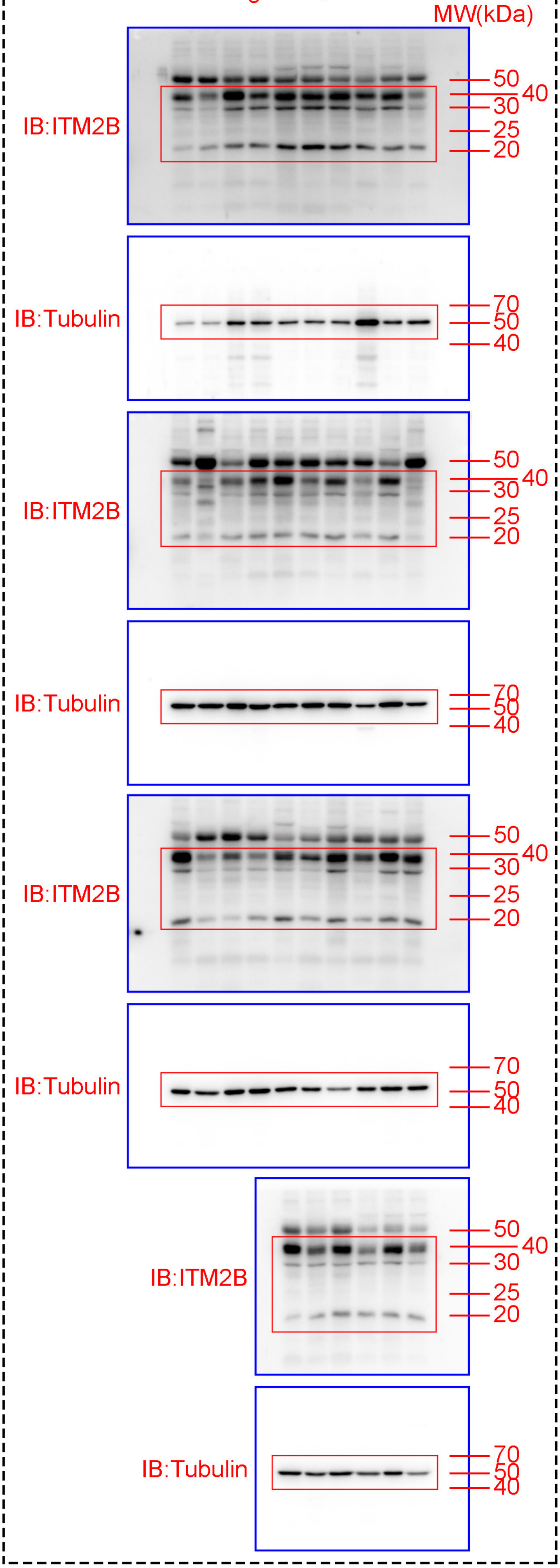

Figure 1E-1

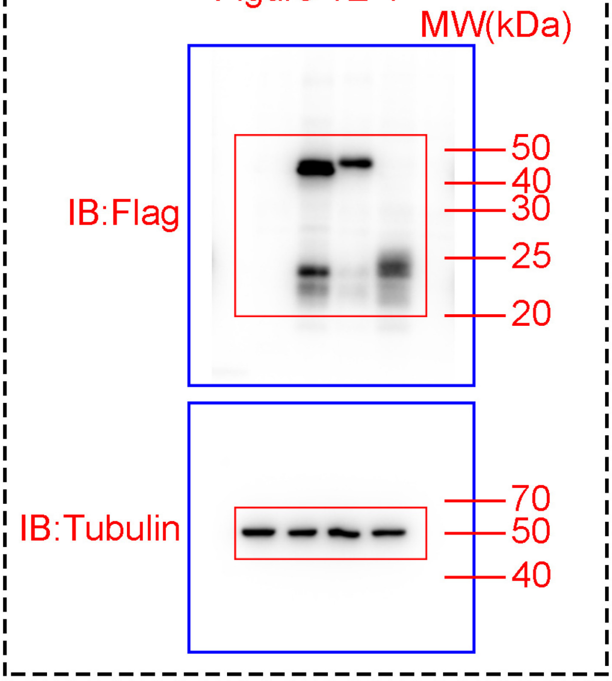

Figure 1E-2

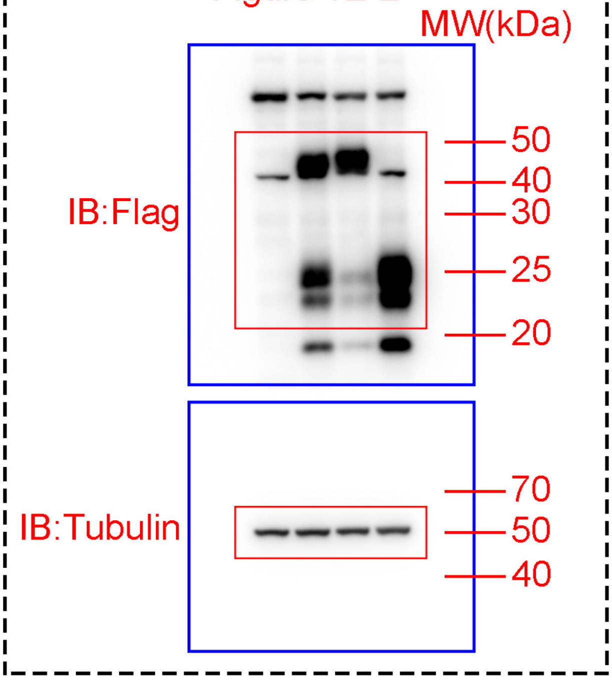

Figure 2D

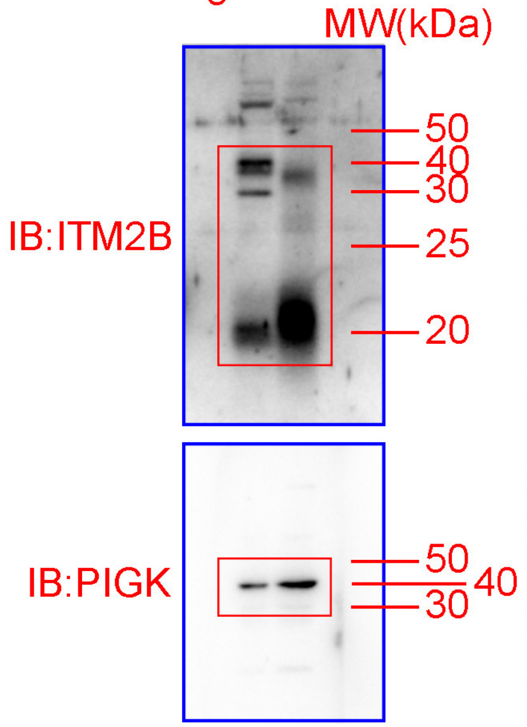

Figure 2E

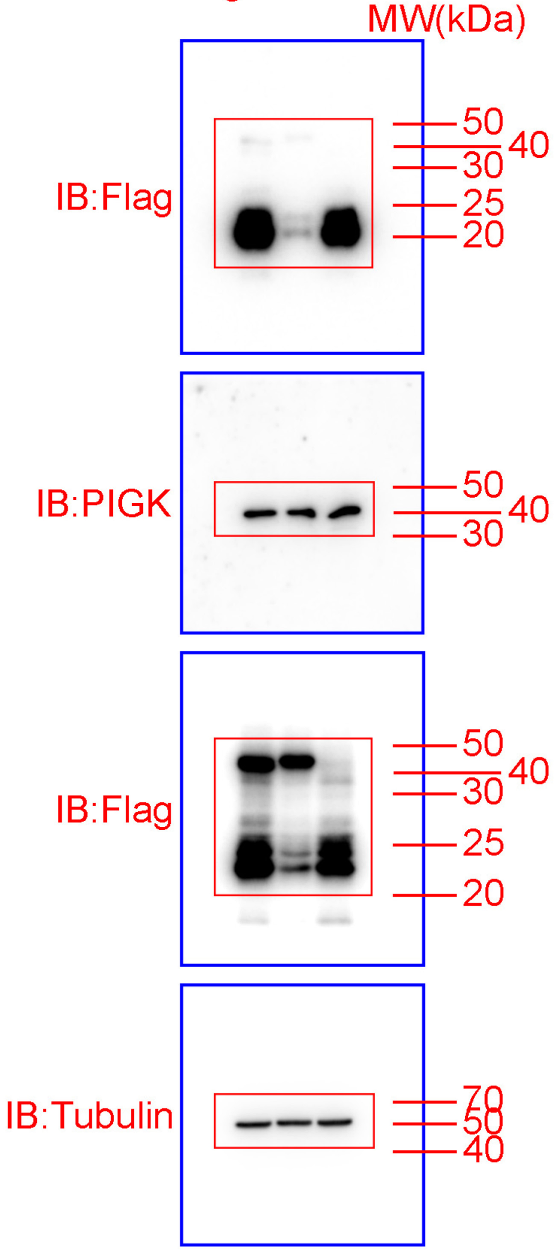

Figure 2K (1-12)

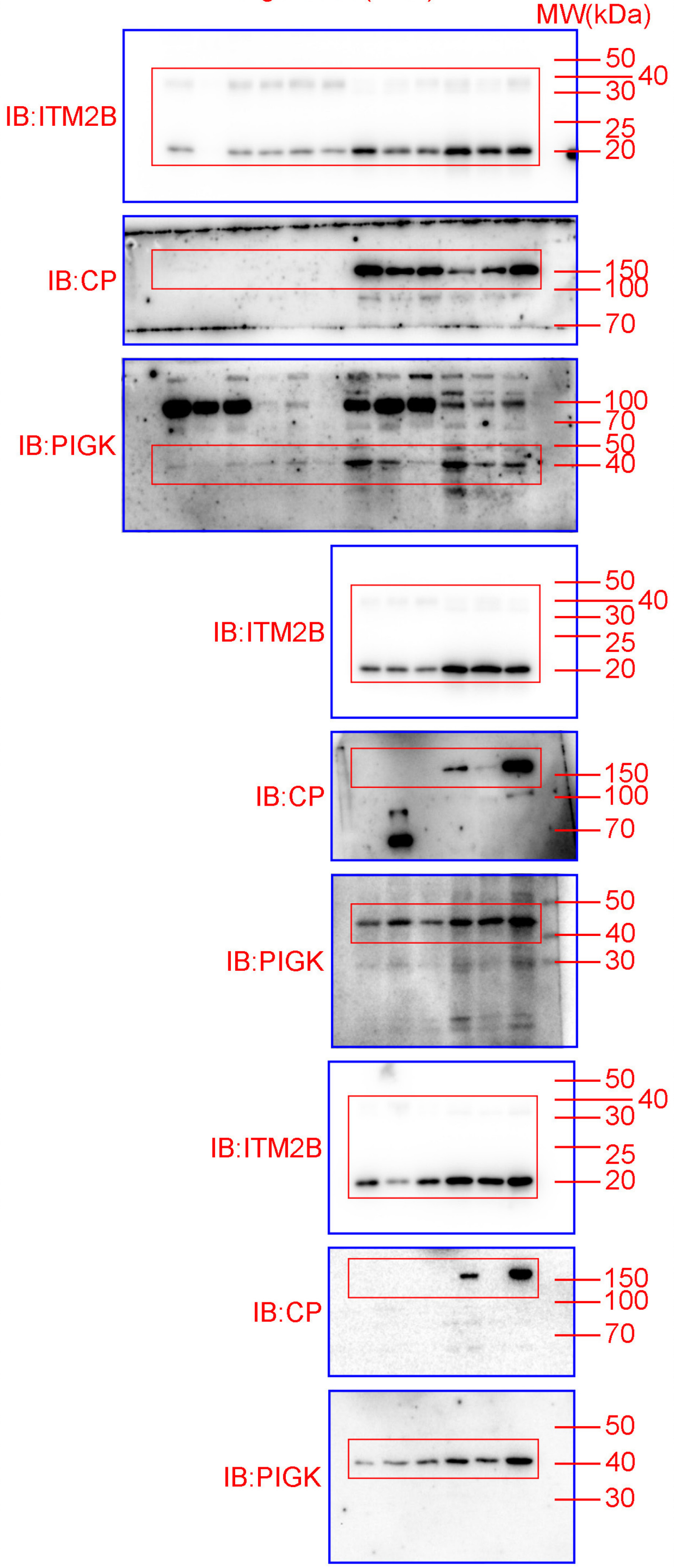

Figure 2K (13-21)

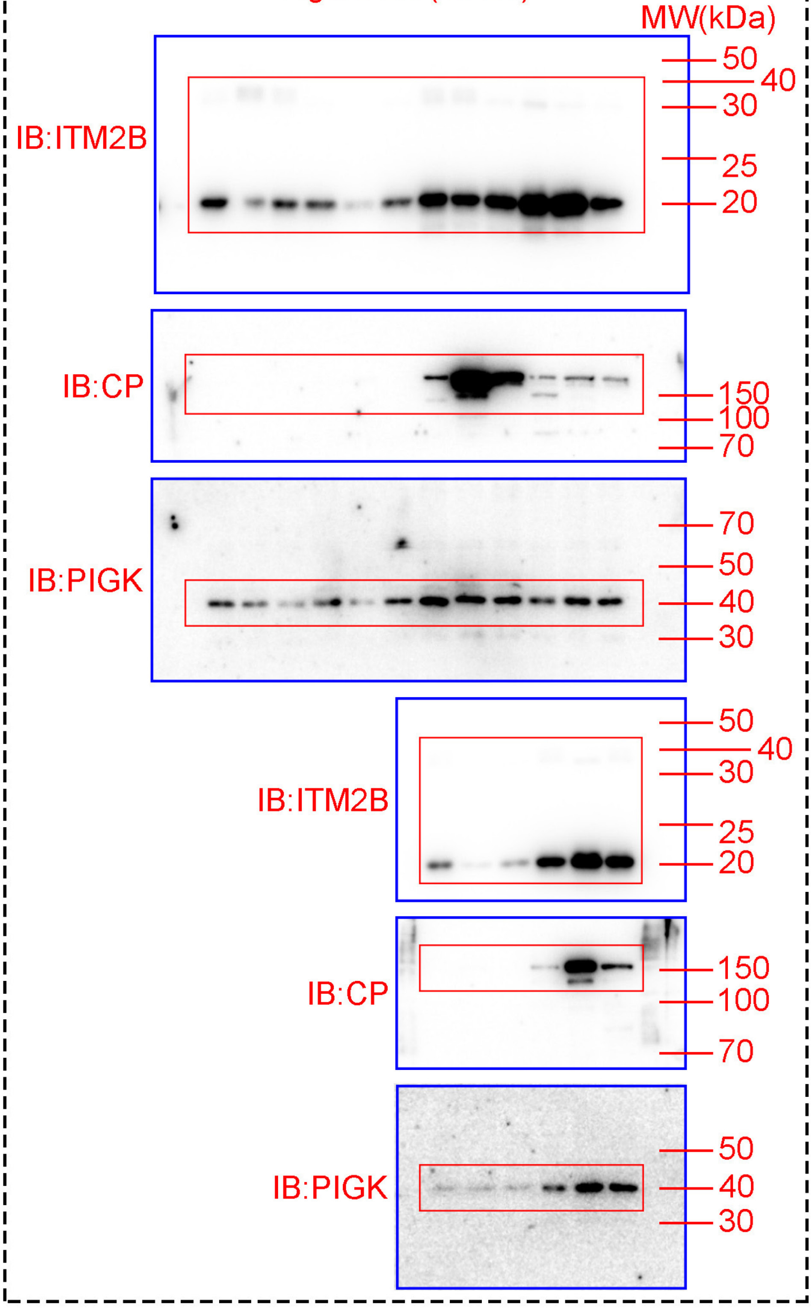

Figure 4C

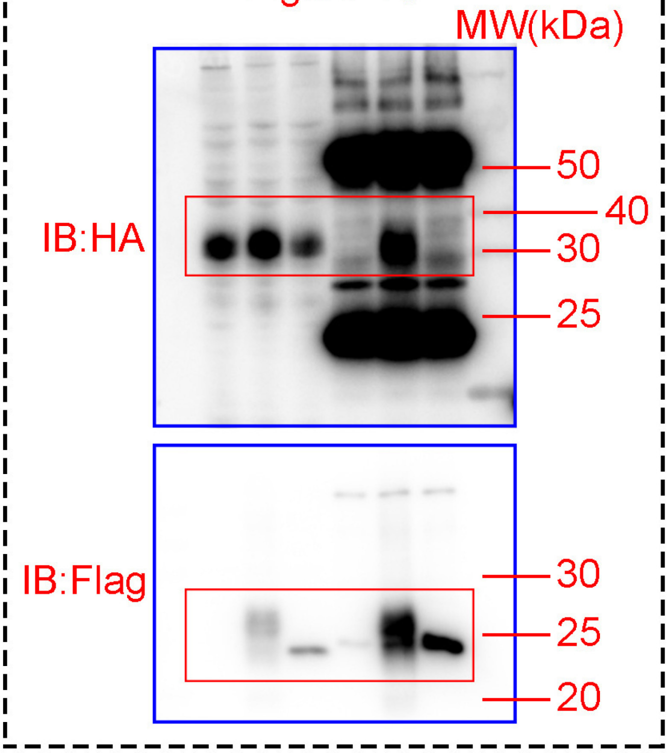

Figure 4E

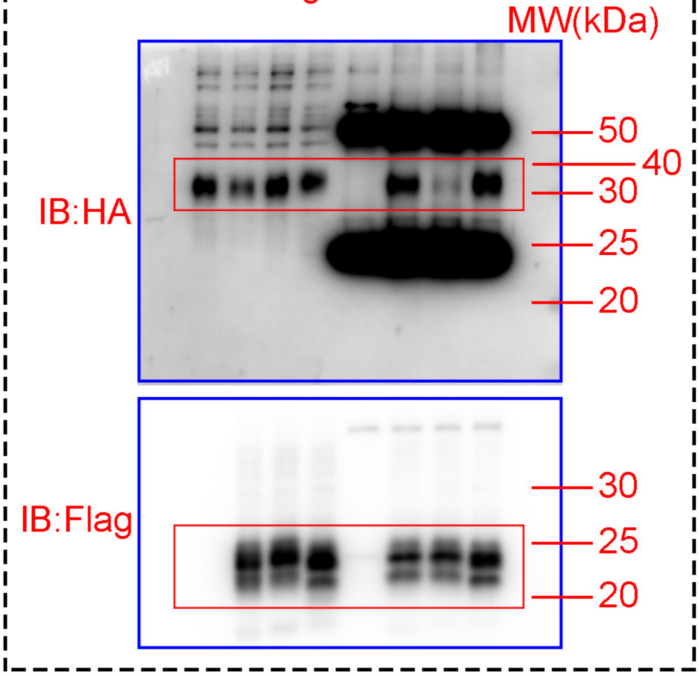

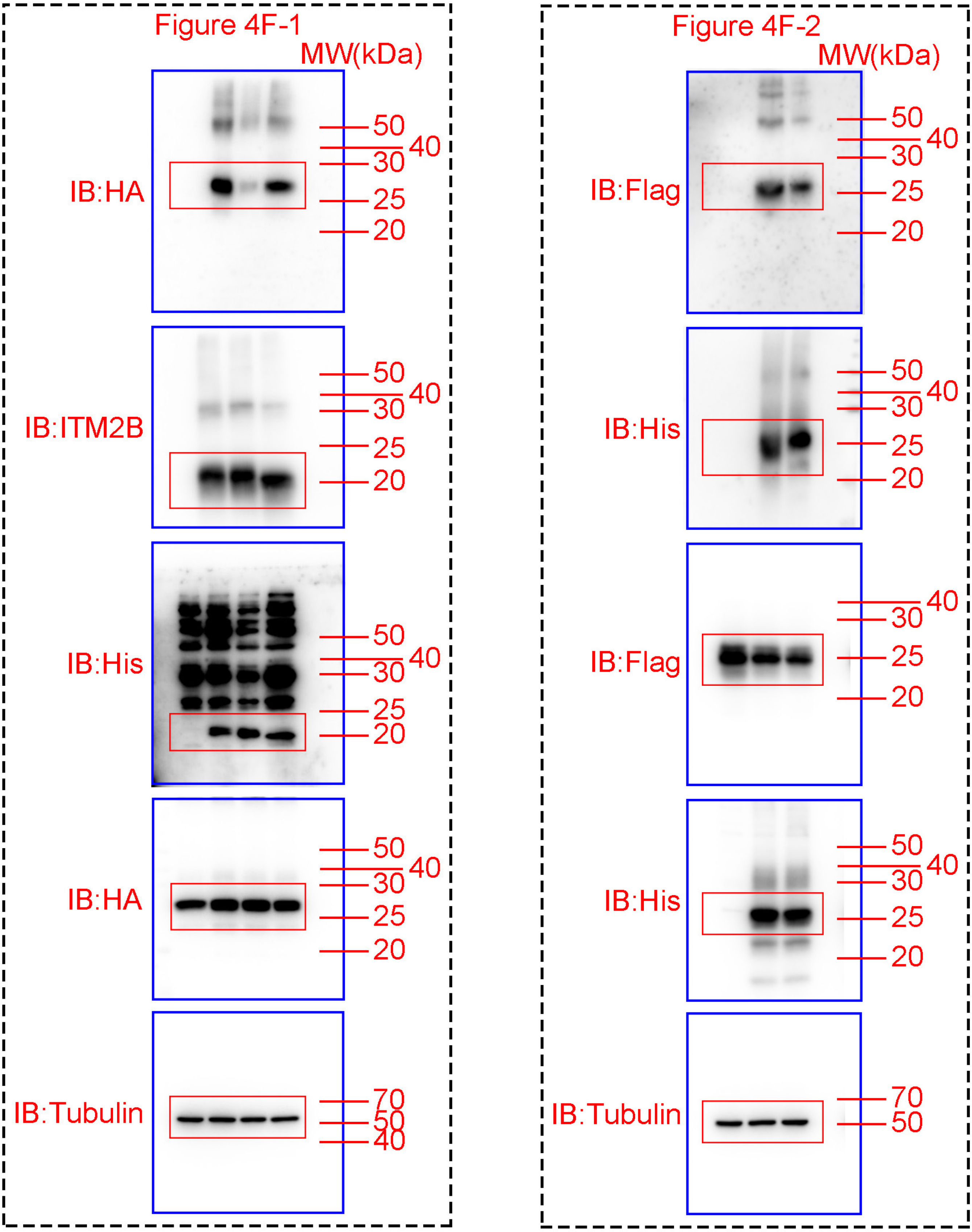
Figure 4F-1
MW(kDa)
IB:HA
50
40
30
25
20
IB:ITM2B
50
40
30
25
20
IB:His
50
40
30
25
20
IB:HA
50
40
30
25
20
IB:Tubulin
70
50
40
Figure 4F-2
MW(kDa)
IB:Flag
50
40
30
25
20
IB:His
50
40
30
25
20
IB:Flag
40
30
25
20
IB:His
50
40
30
25
20
IB:Tubulin
70
50

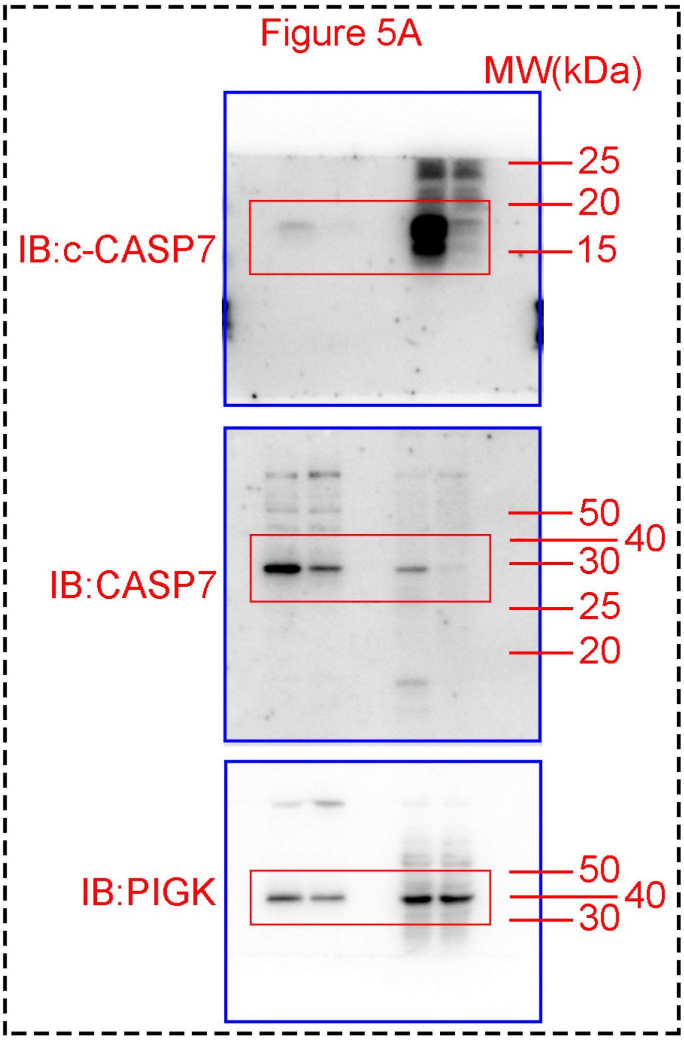
Figure 5A
MW(kDa)
IB:c-CASP7
25
20
15
IB:CASP7
50
40
30
25
20
IB:PIGK
50
40
30

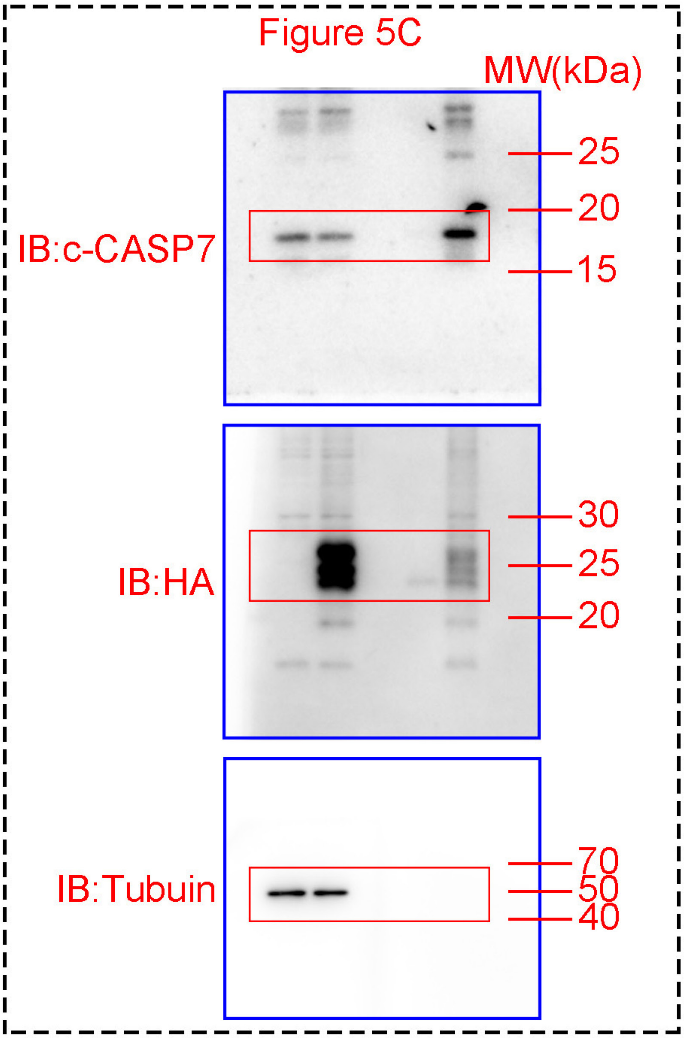
Figure 5C
MW(kDa)
IB:c-CASP7
25
20
15
IB:HA
30
25
20
IB:Tubuin
70
50
40

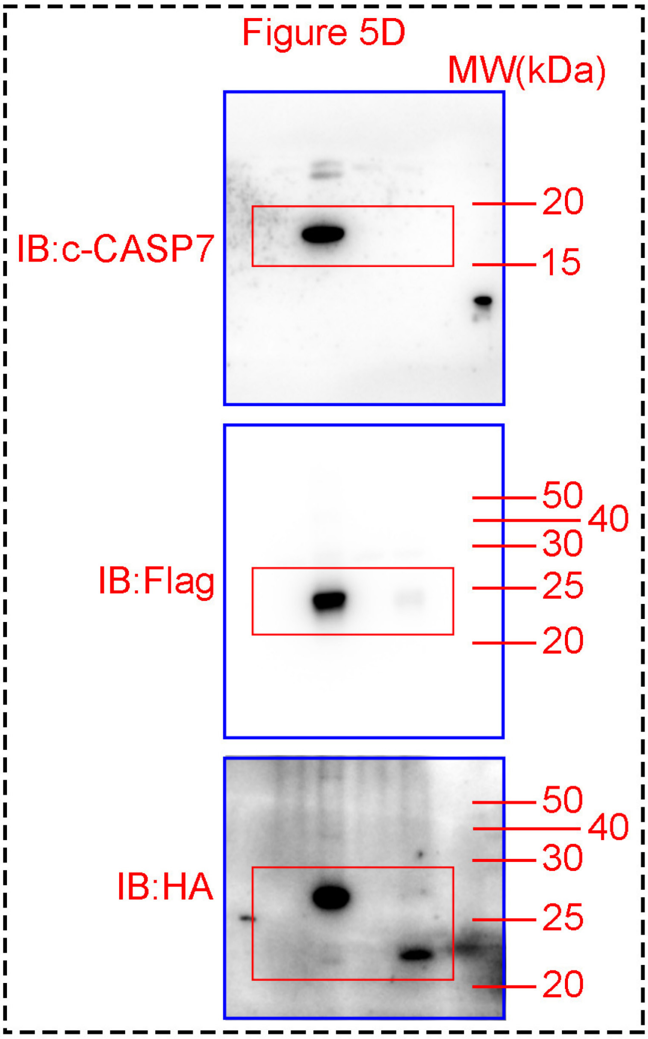
Figure 5D
MW(kDa)
IB:c-CASP7
20
15
IB:Flag
50
40
30
25
20
IB:HA
50
40
30
25
20

Figure 5F

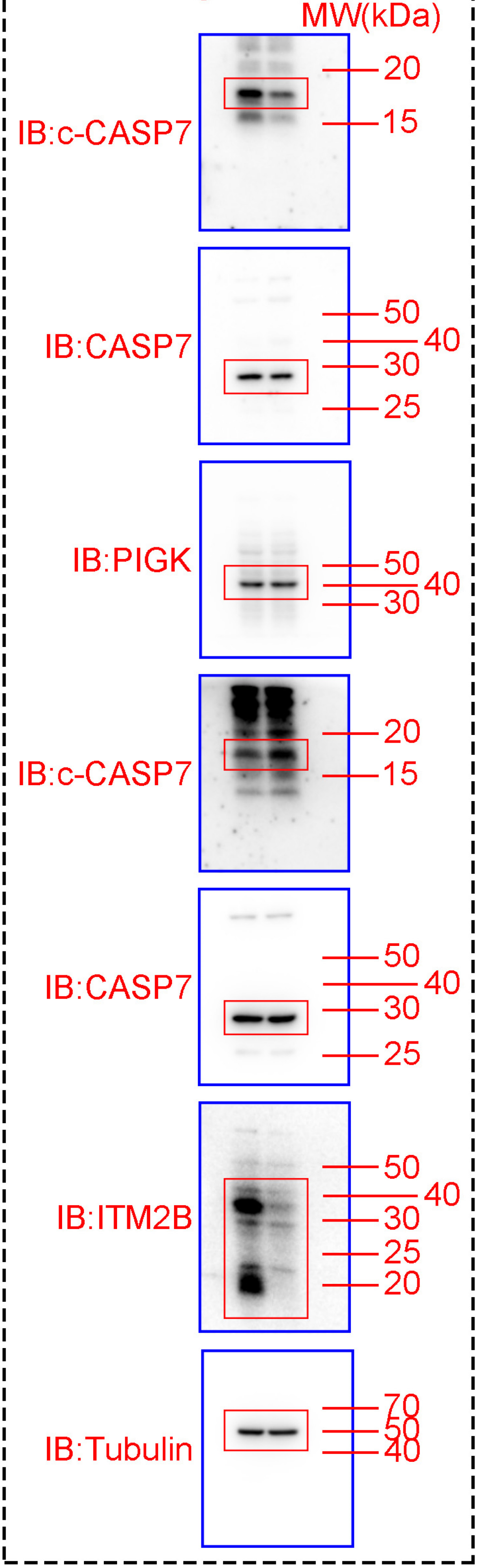

Figure 5G

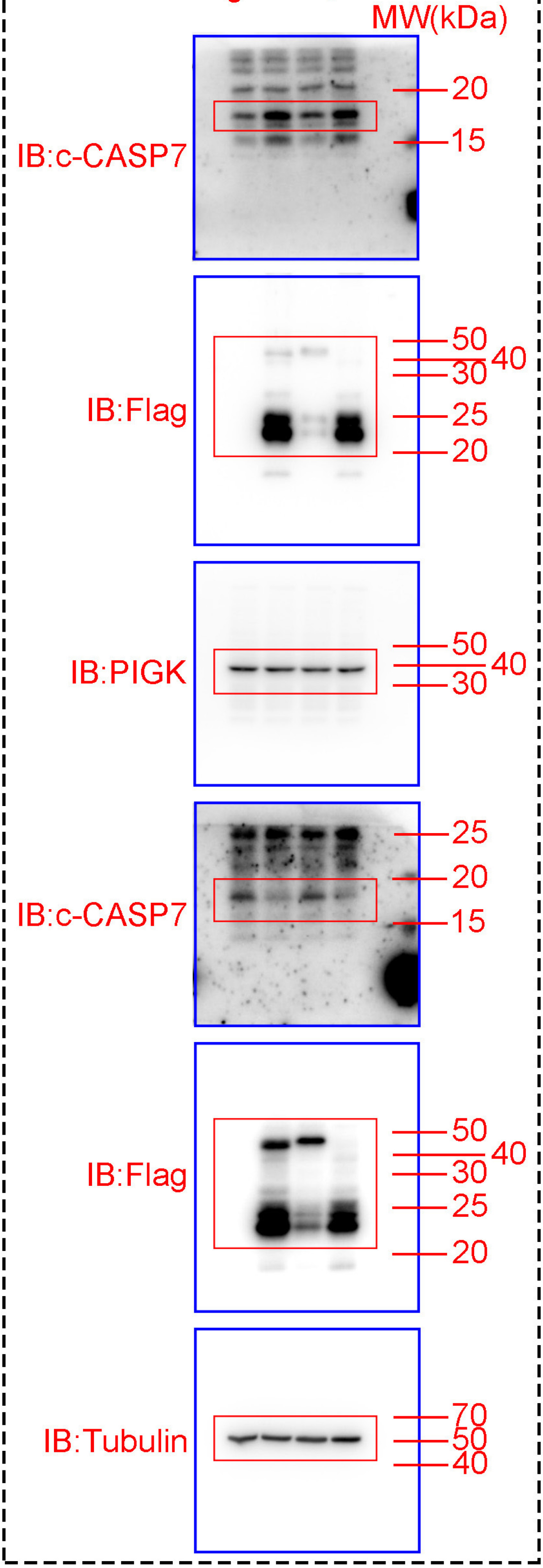

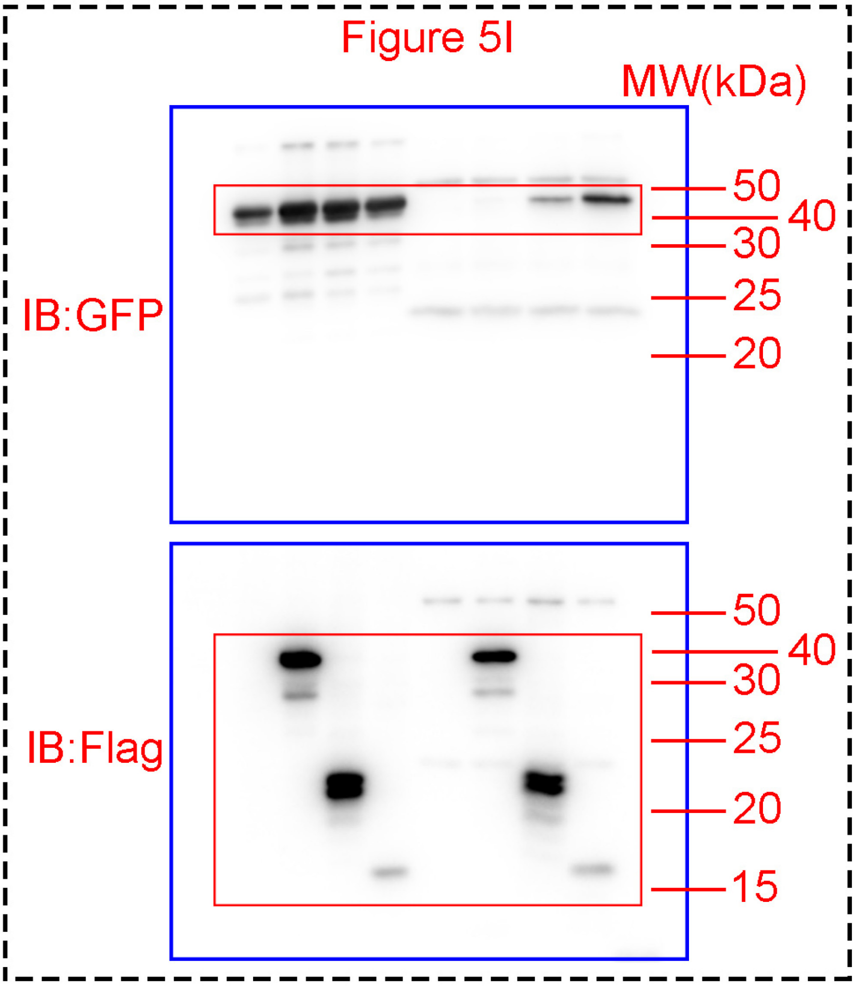
Figure 5I
MW(kDa)
IB:GFP
50
40
30
25
20
IB:Flag
50
40
30
25
20
15

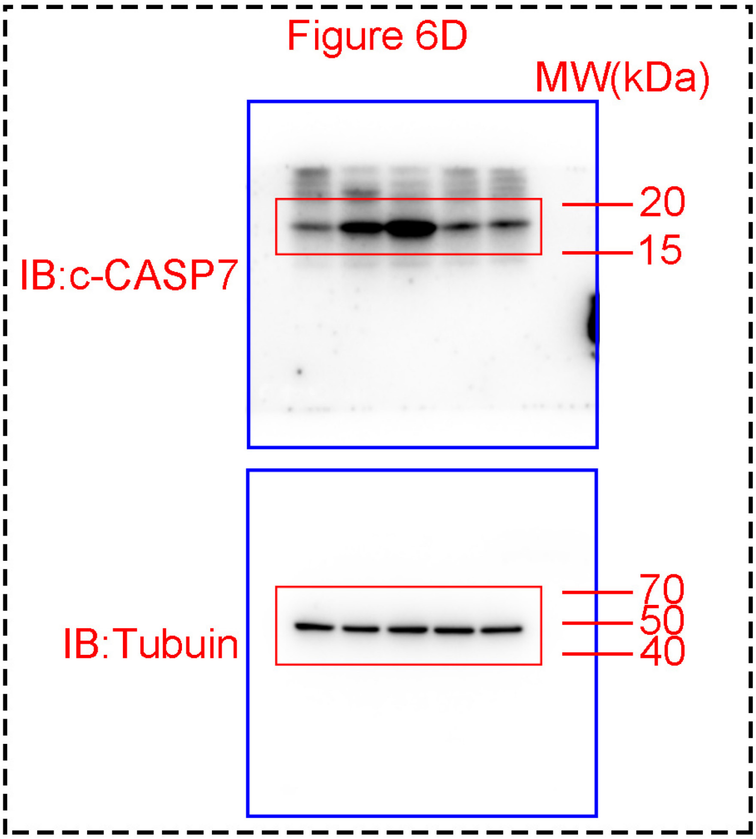
Figure 6D
MW(kDa)
IB:c-CASP7
20
15
IB:Tubuin
70
50
40

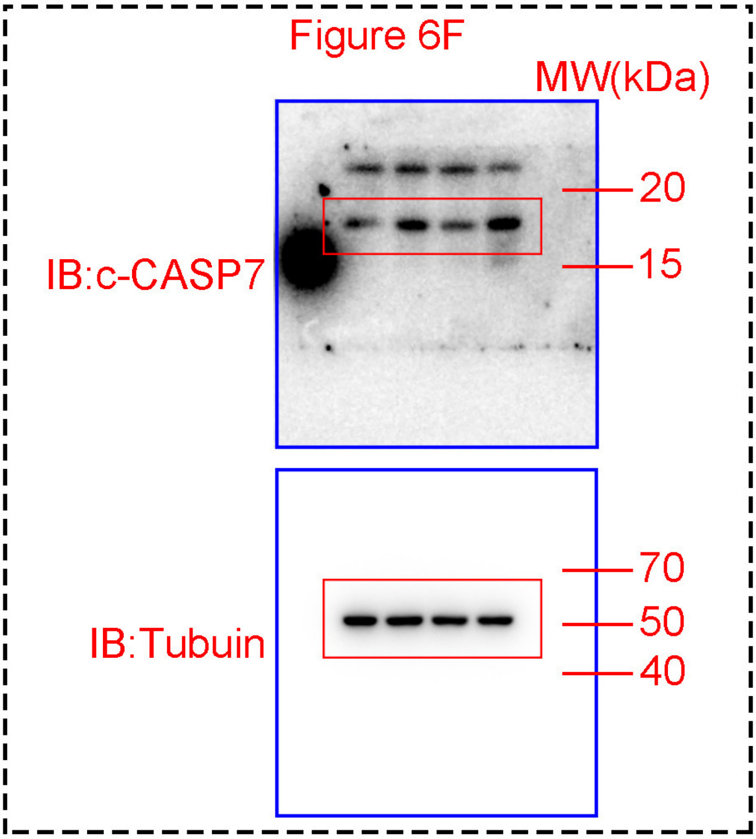
Figure 6F
MW(kDa)
IB:c-CASP7
20
15
IB:Tubuin
70
50
40

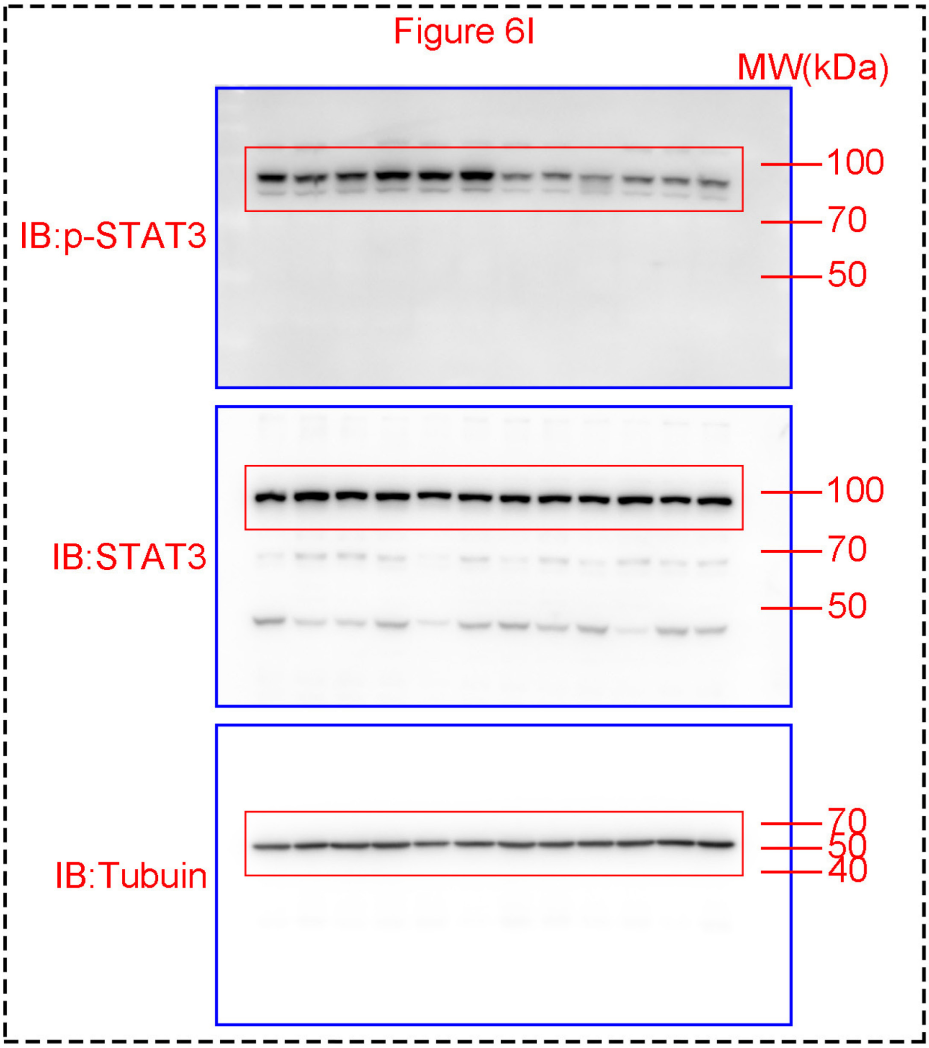
Figure 6I
MW(kDa)
IB:p-STAT3
100
70
50
IB:STAT3
100
70
50
IB:Tubuin
70
50
40

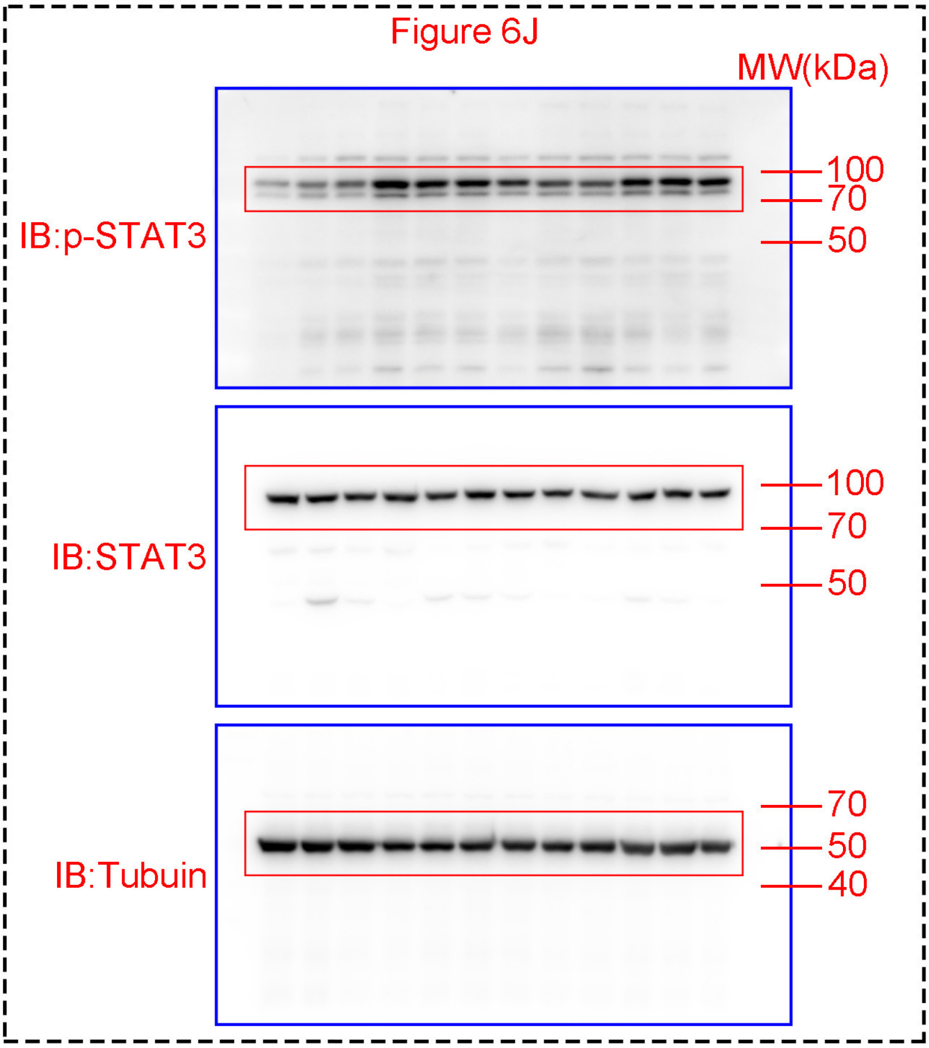
Figure 6J
MW(kDa)
IB:p-STAT3
100
70
50
IB:STAT3
100
70
50
IB:Tubuin
70
50
40

Figure 6K

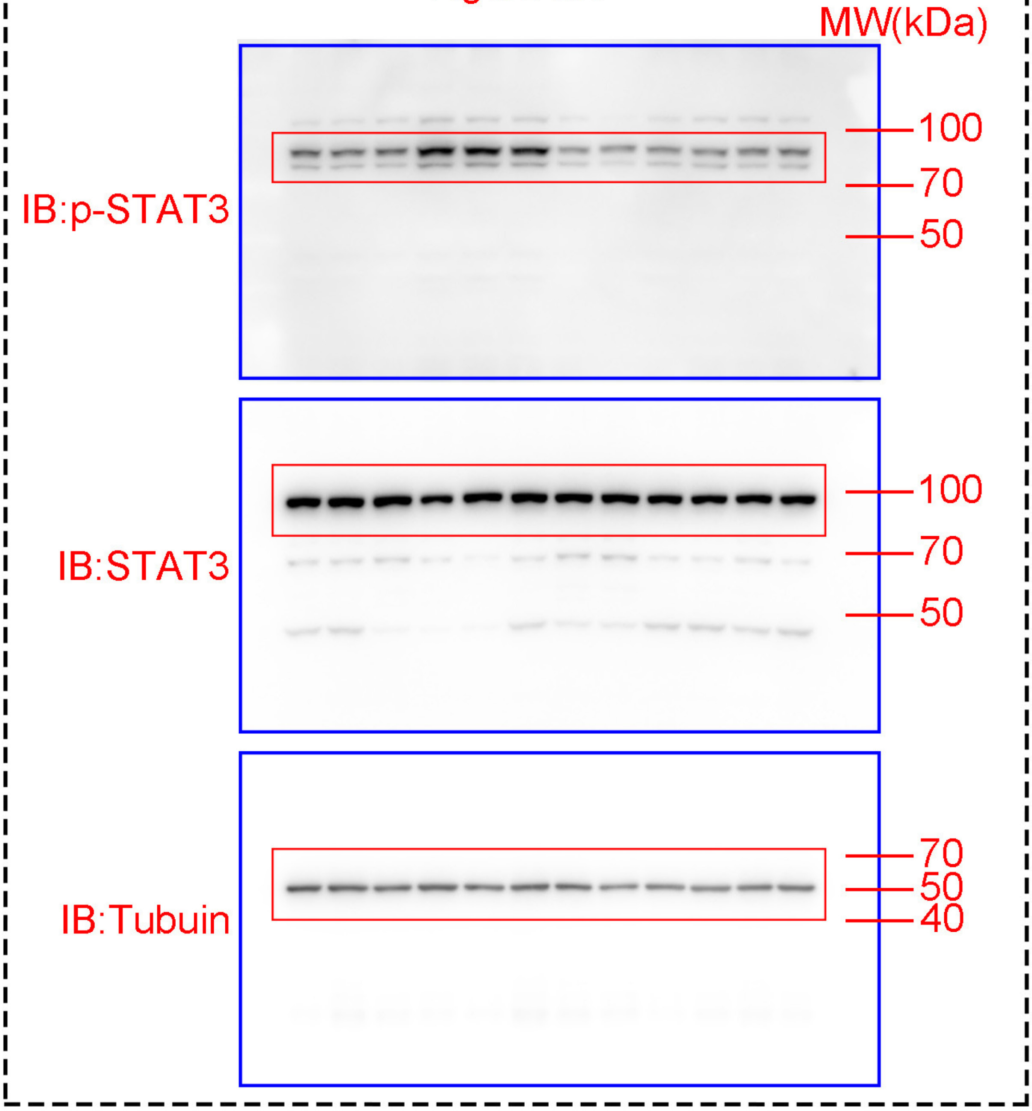

Figure 7A

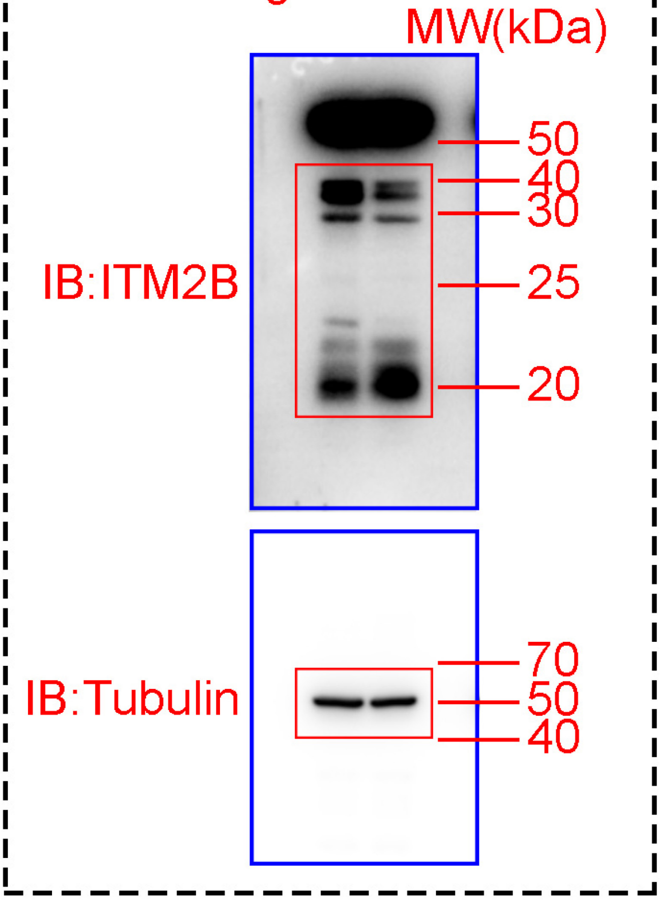

Figure 7B

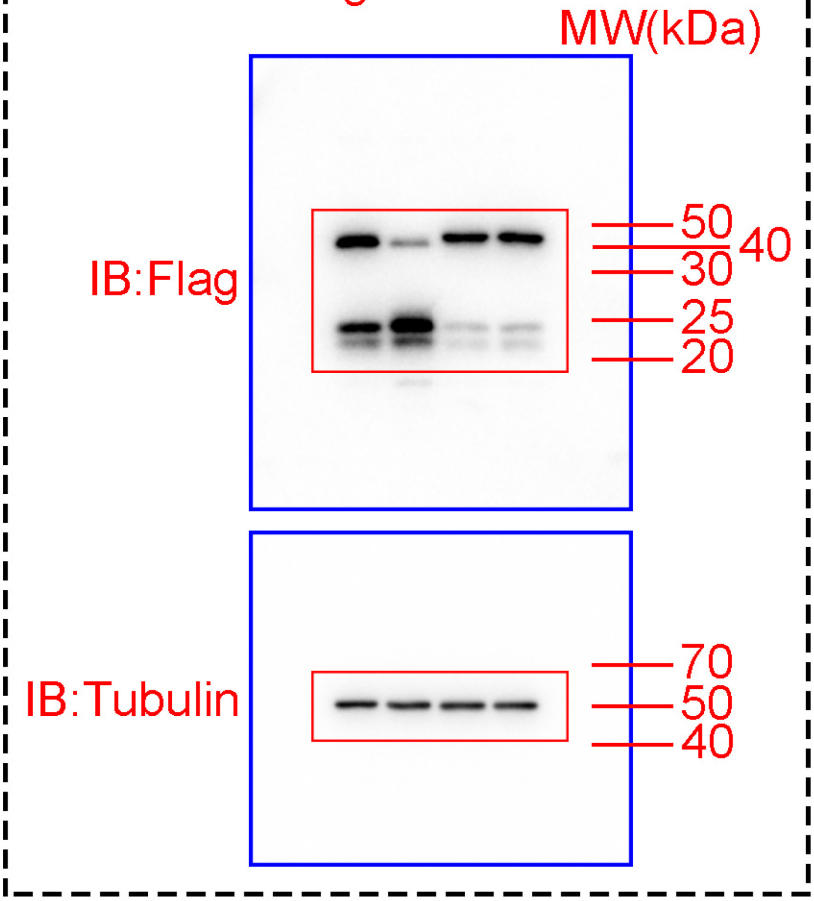

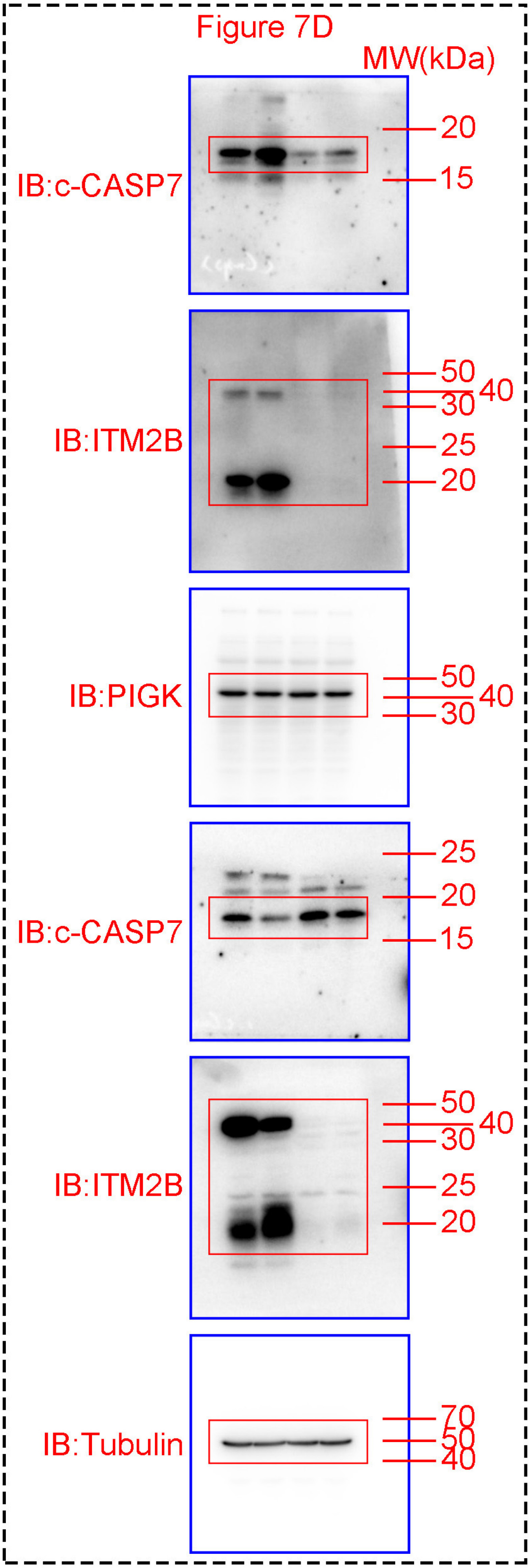
Figure 7D
MW(kDa)
IB:c-CASP7
20
15
IB:ITM2B
50
40
30
25
20
IB:PIGK
50
40
30
IB:c-CASP7
25
20
15
IB:ITM2B
50
40
30
25
20
IB:Tubulin
70
50
40

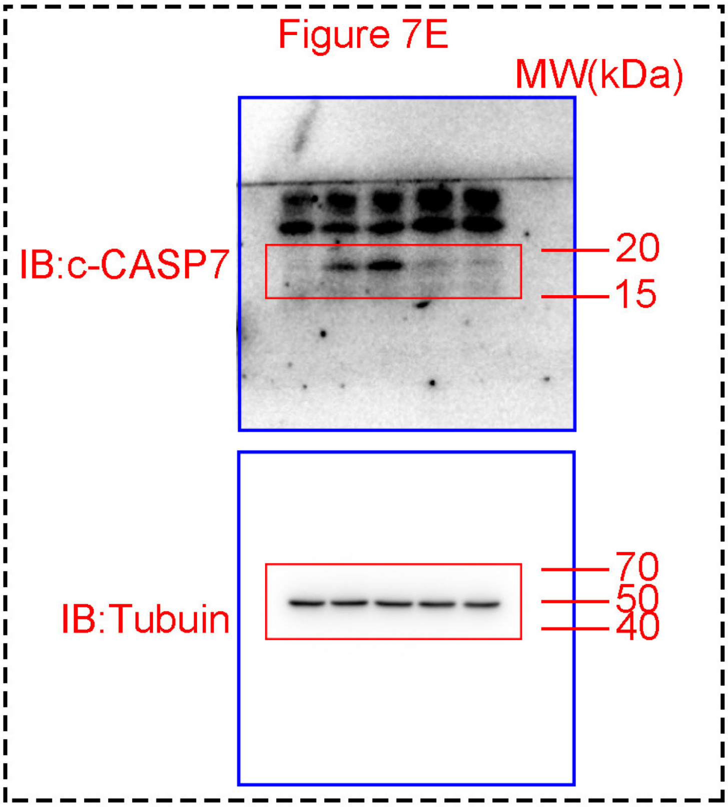
Figure 7E
MW(kDa)
IB:c-CASP7
20
15
IB:Tubuin
70
50
40

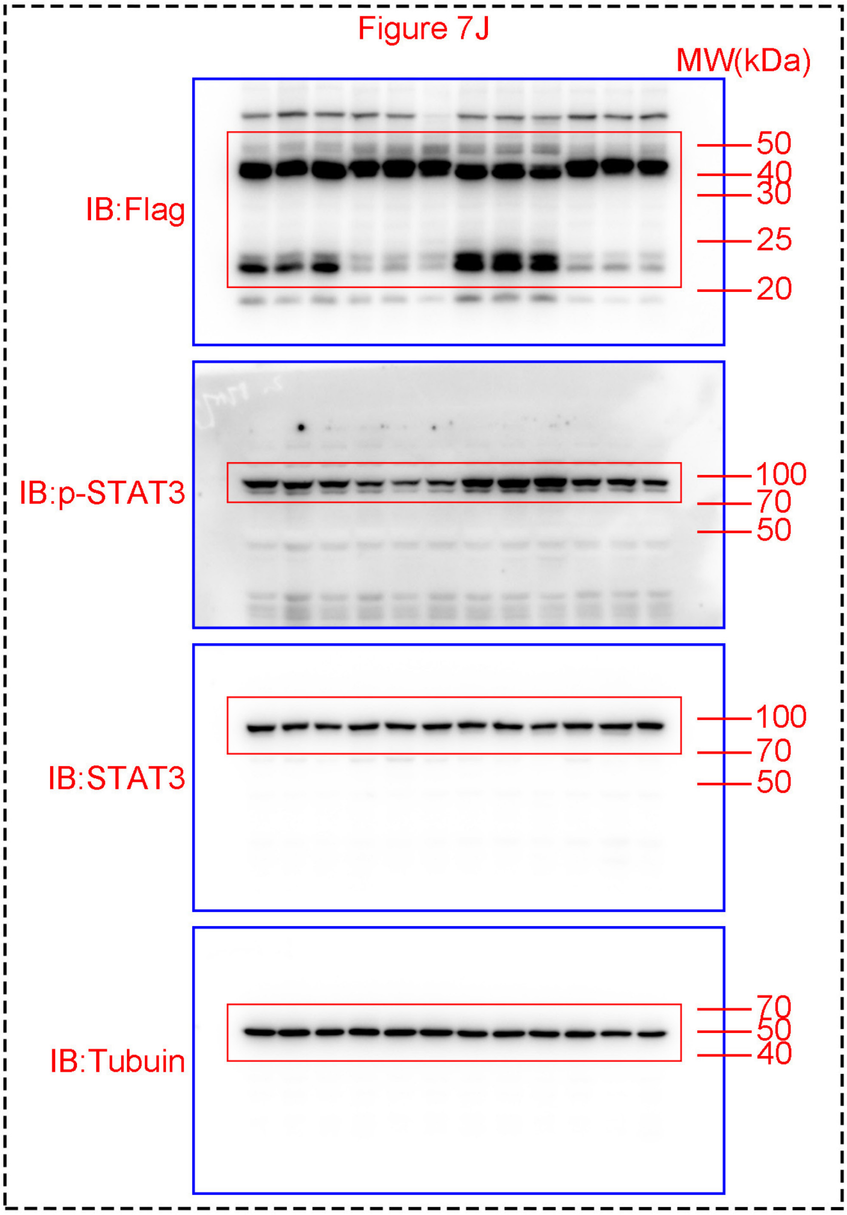
Figure 7J
MW(kDa)
IB:Flag
50
40
30
25
20
IB:p-STAT3
100
70
50
IB:STAT3
100
70
50
IB:Tubuin
70
50
40

Figure S1C

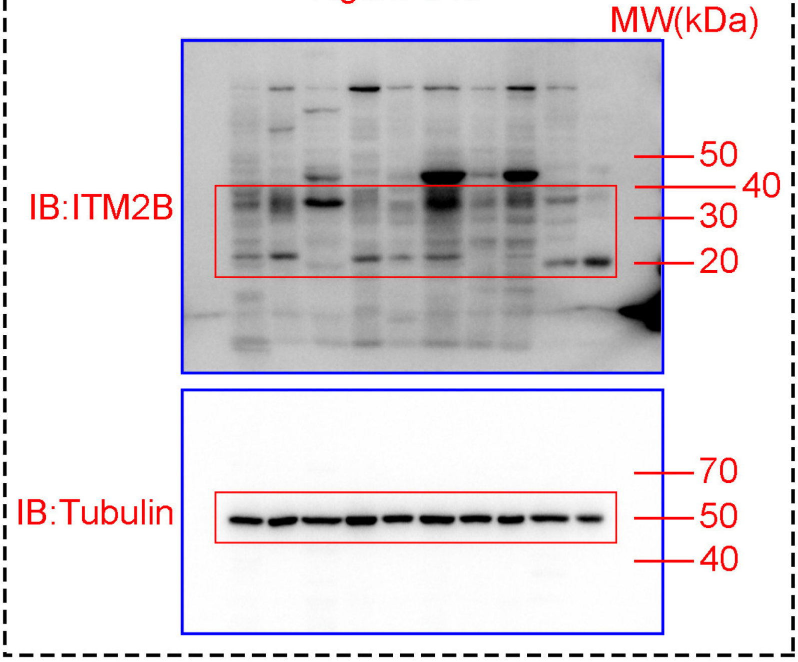

Figure S1D

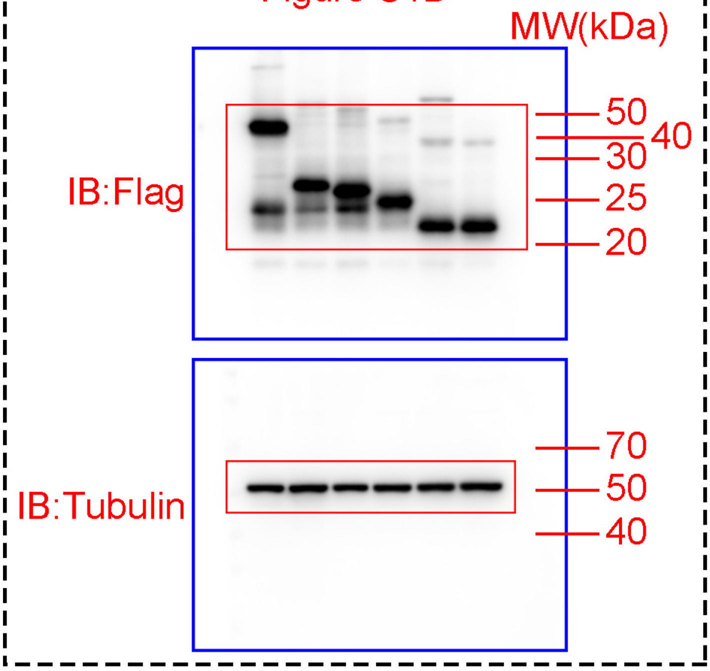

Figure S1E

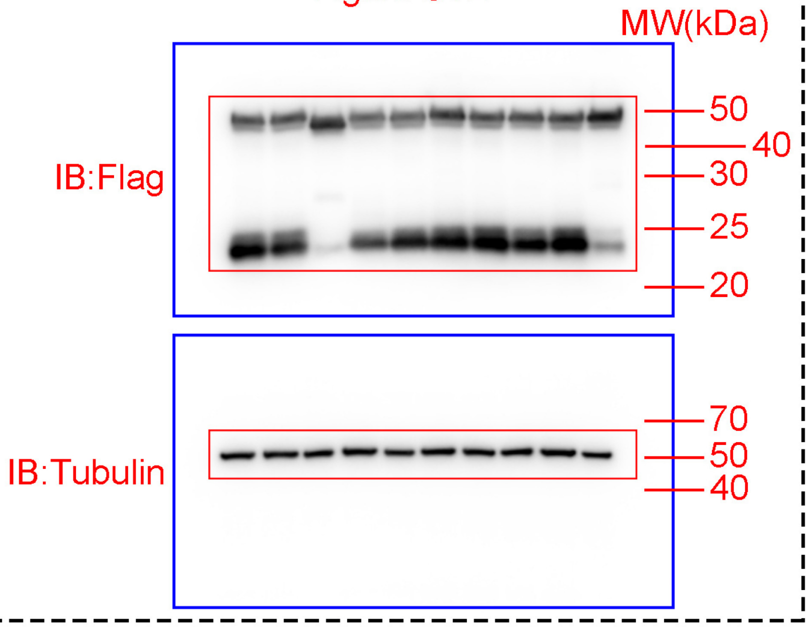

Figure S2C

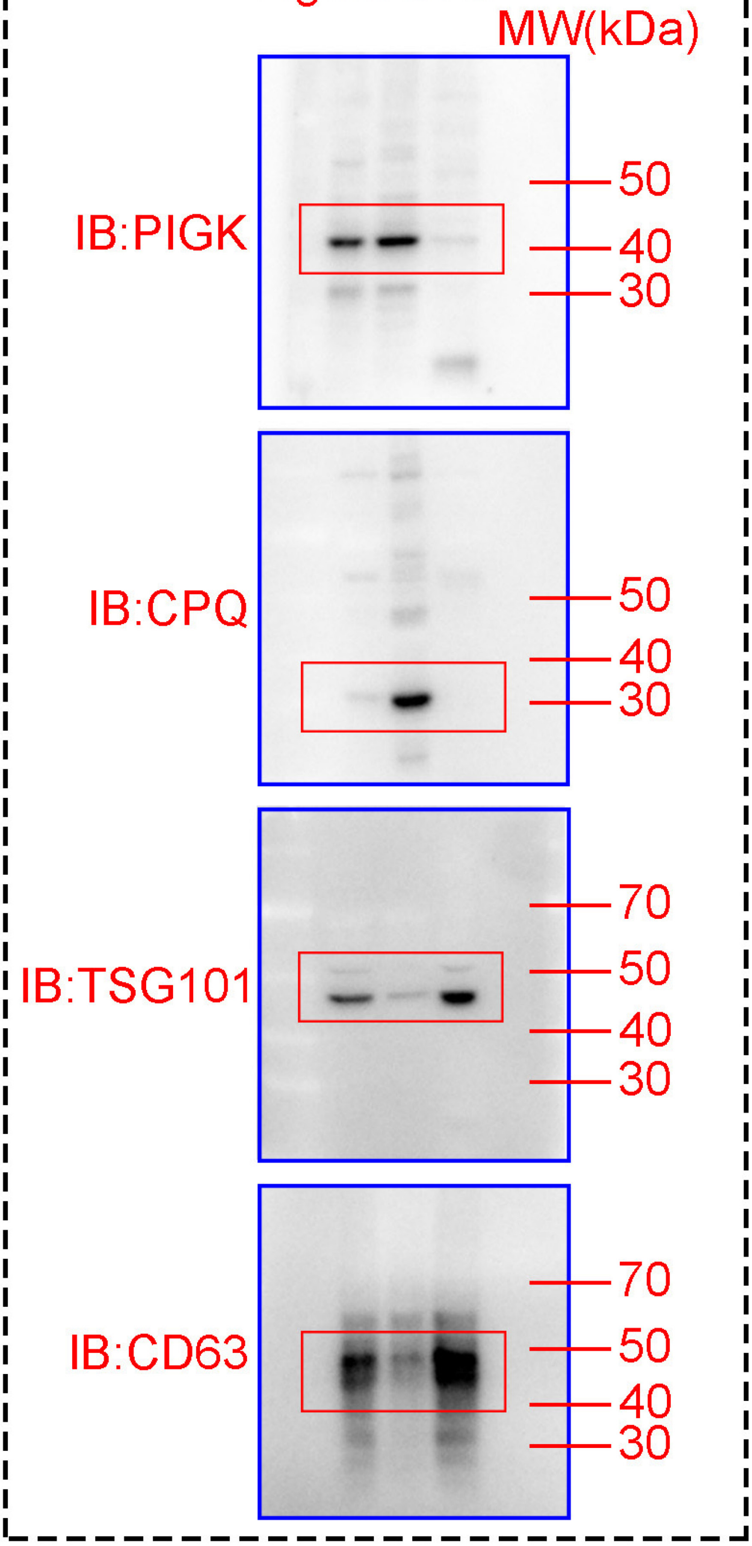

Figure S3B

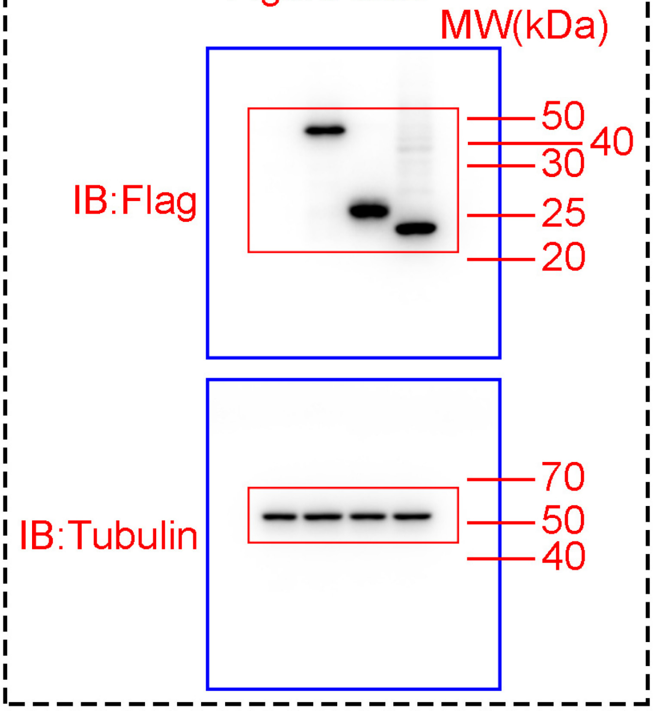

Figure S3H

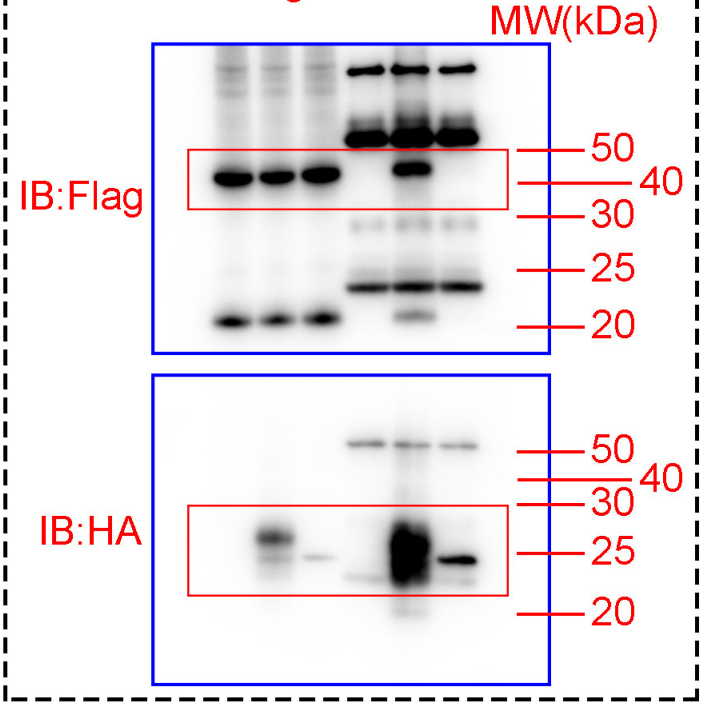

Figure S4E-1

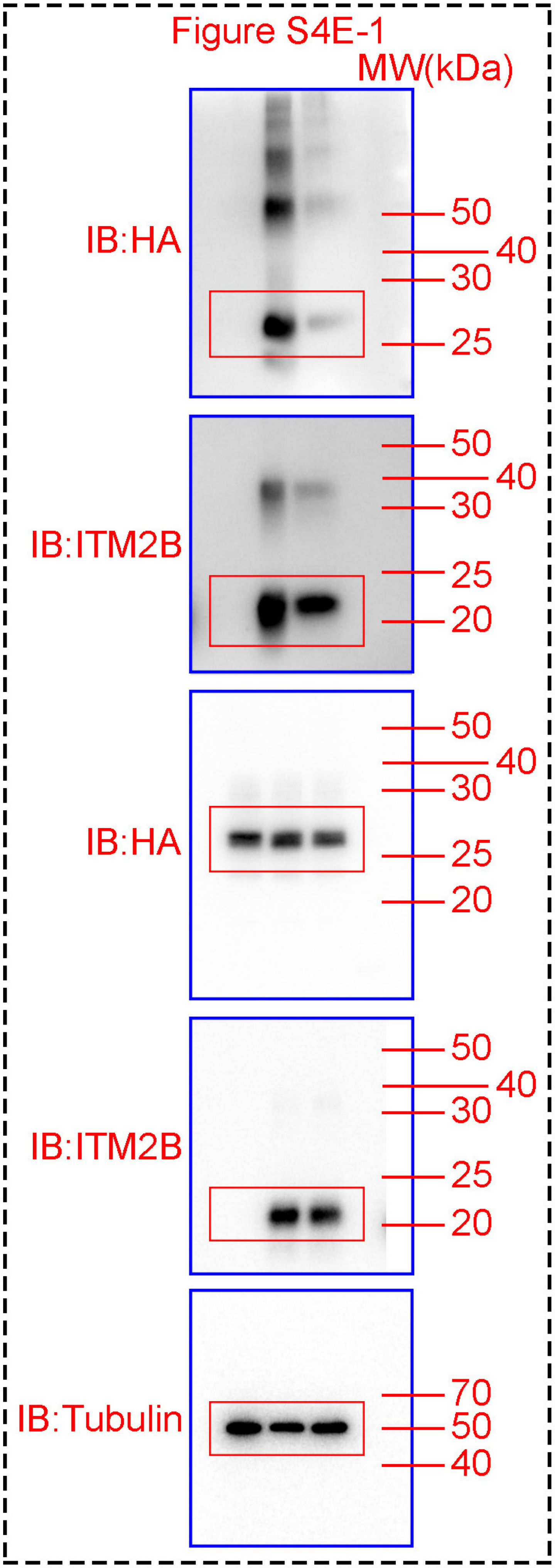

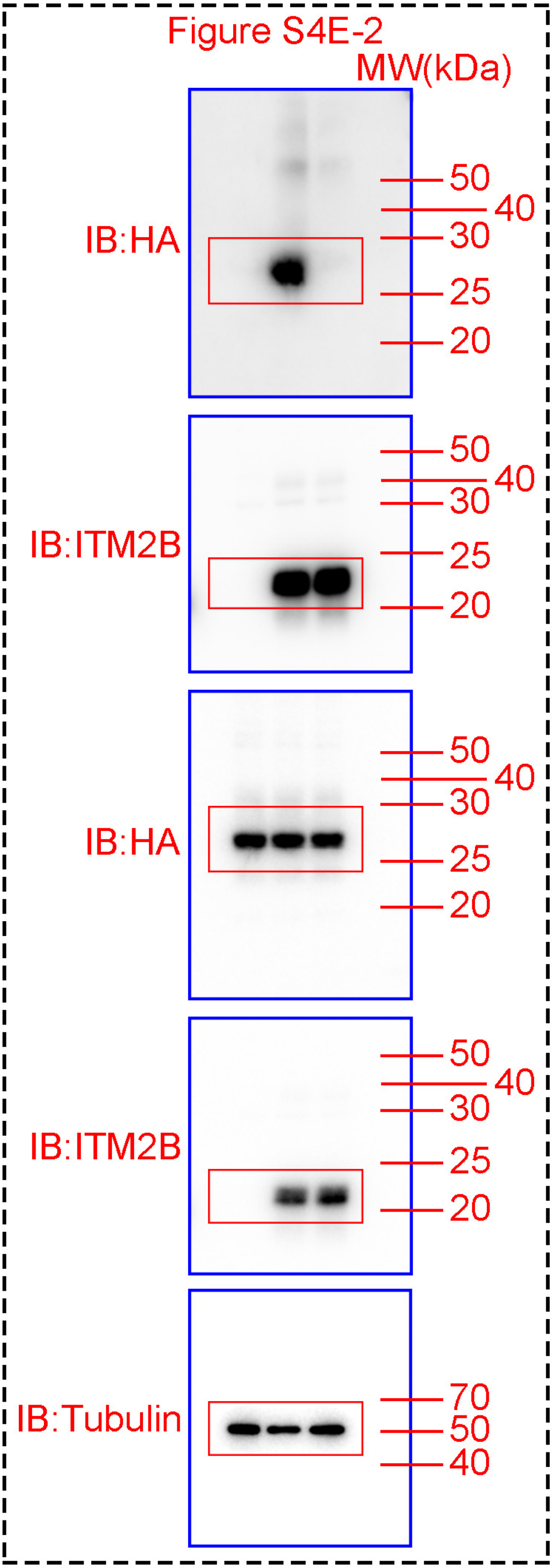
Figure S4E-2
MW(kDa)
IB:HA
50
40
30
25
20
IB:ITM2B
50
40
30
25
20
IB:HA
50
40
30
25
20
IB:ITM2B
50
40
30
25
20
IB:Tubulin
70
50
40

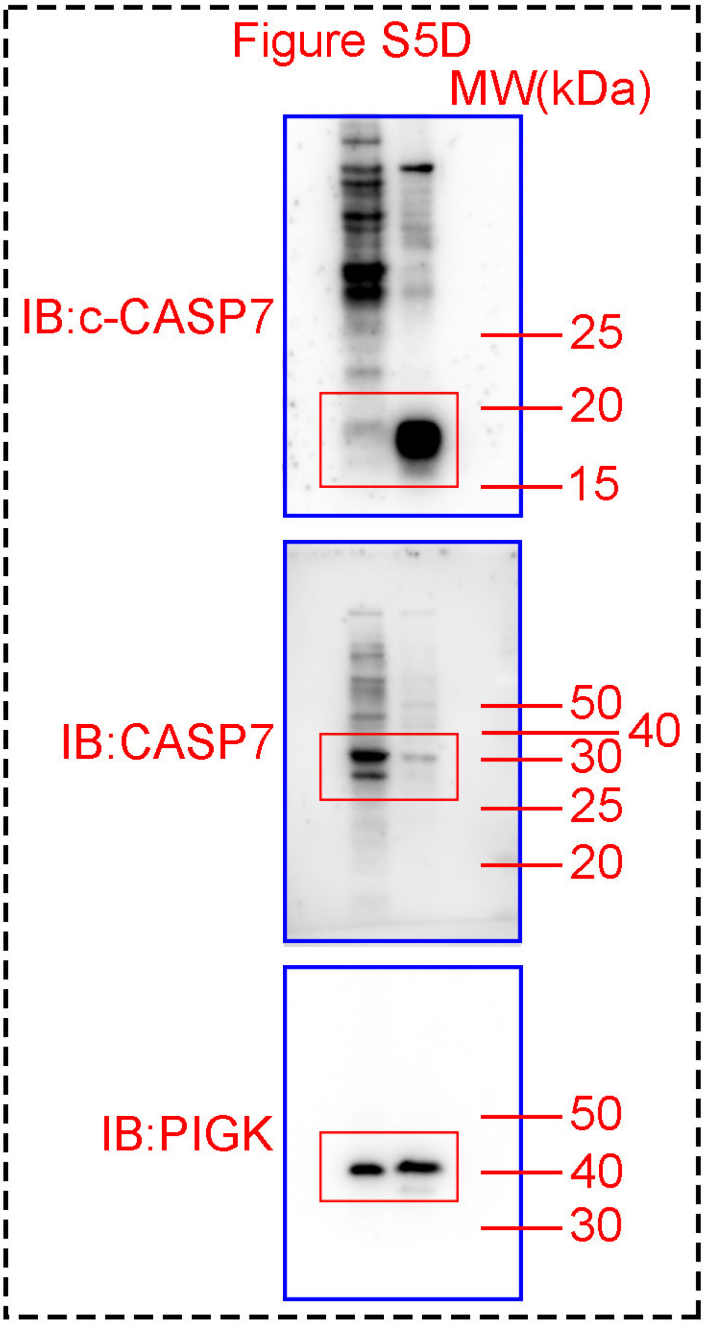
Figure S5D
MW(kDa)
IB:c-CASP7
25
20
15
IB:CASP7
50
40
30
25
20
IB:PIGK
50
40
30

Figure S5F

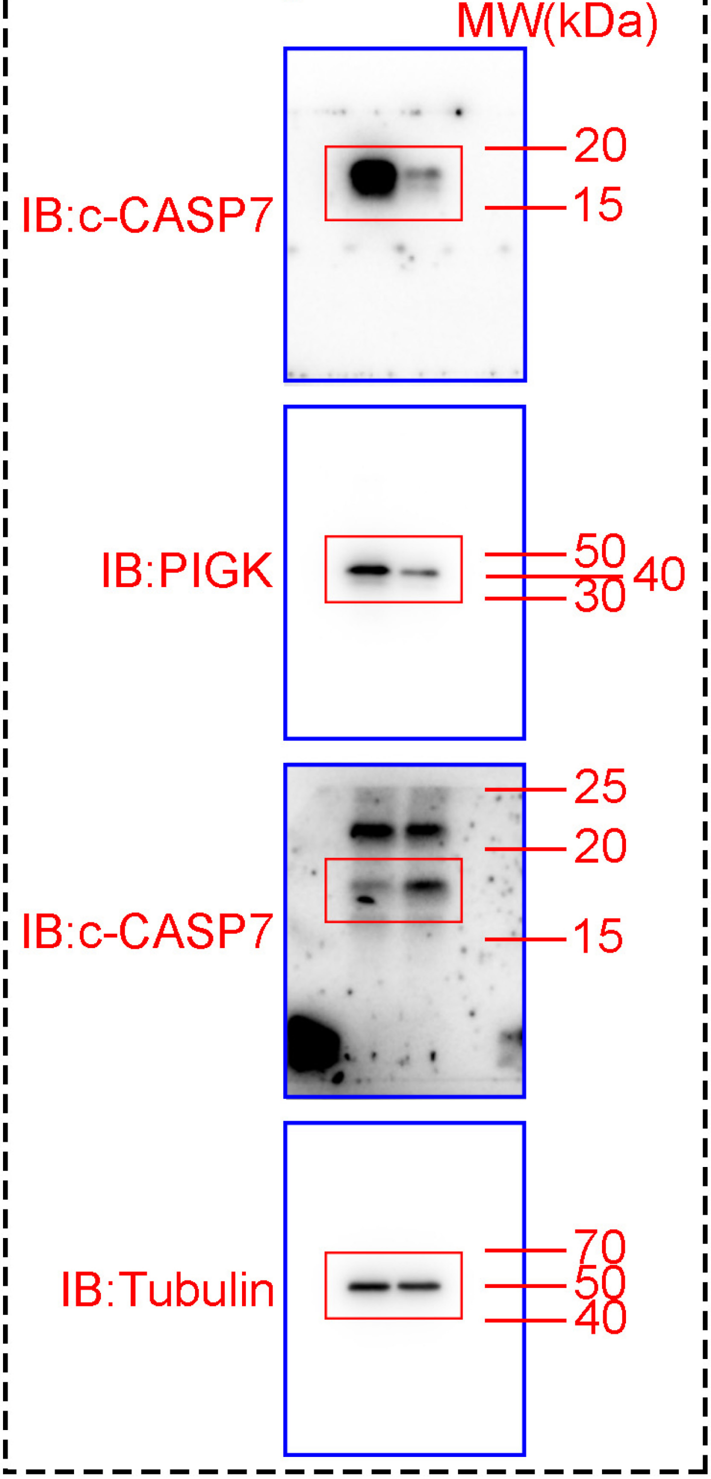

Figure S5G

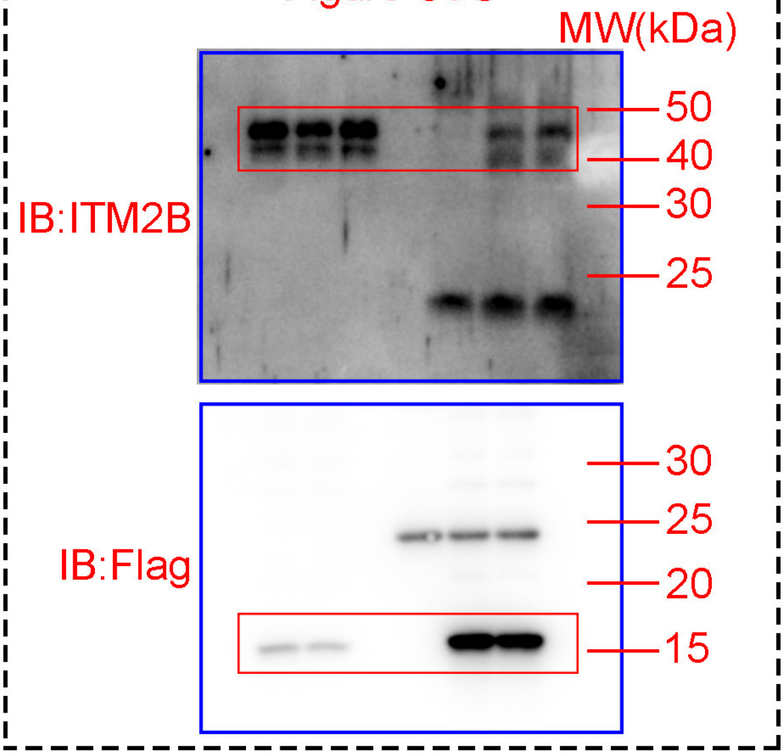

Figure S5H

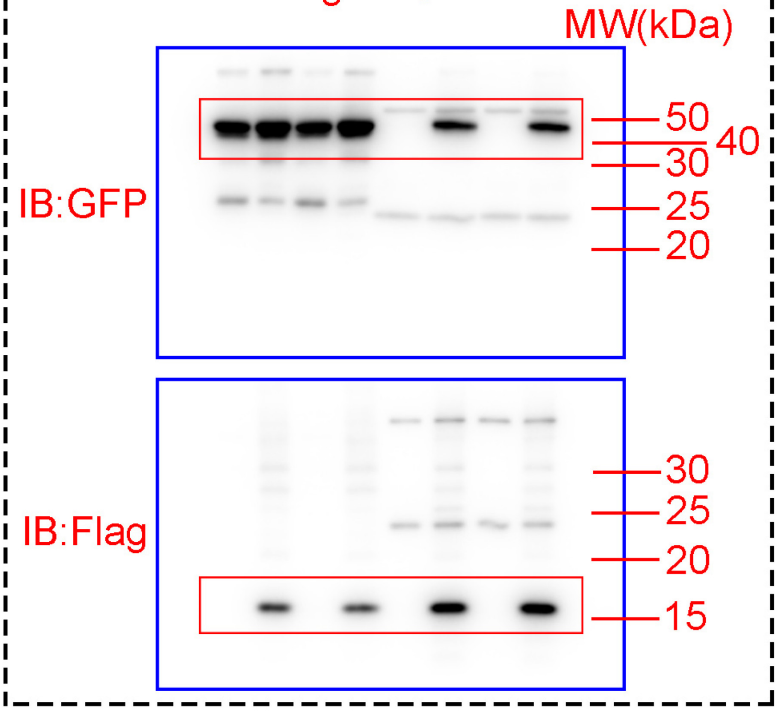

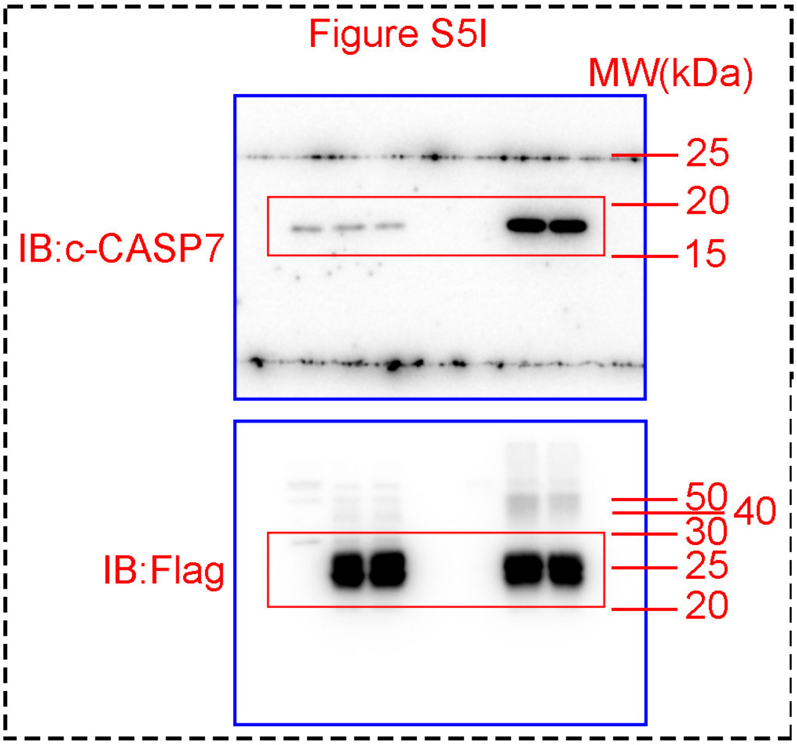
Figure S5I
MW(kDa)
IB:c-CASP7
25
20
15
IB:Flag
50
40
30
25
20

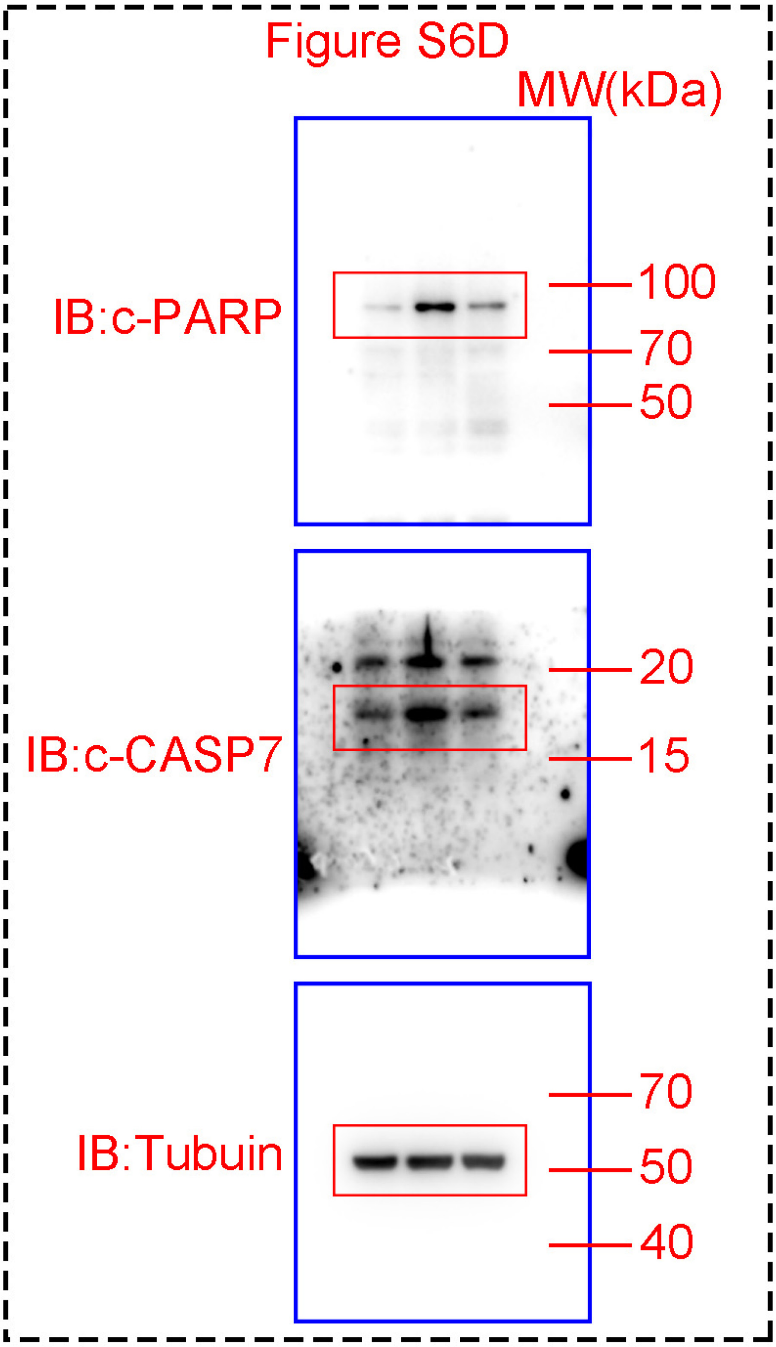
Figure S6D
MW(kDa)
IB:c-PARP
100
70
50
IB:c-CASP7
20
15
IB:Tubuin
70
50
40

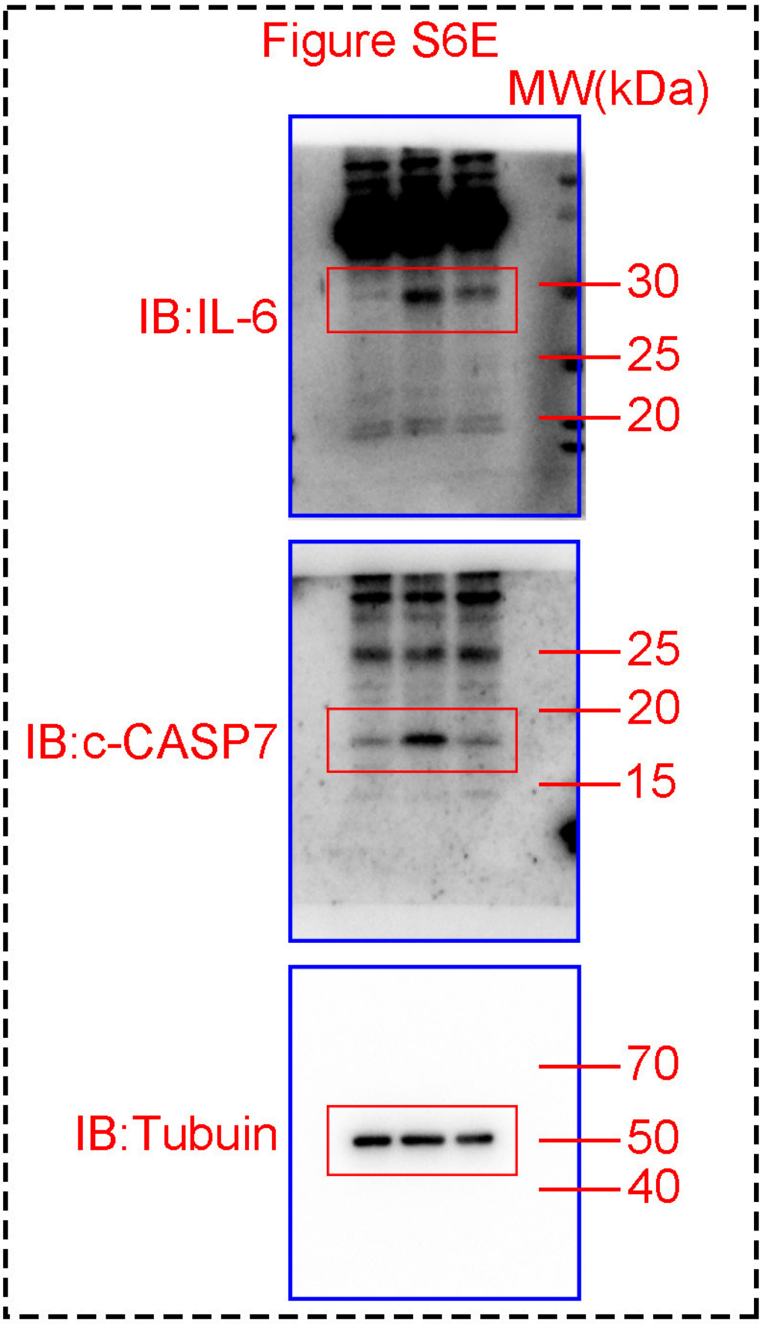
Figure S6E
MW(kDa)
IB:IL-6
30
25
20
IB:c-CASP7
25
20
15
IB:Tubuin
70
50
40

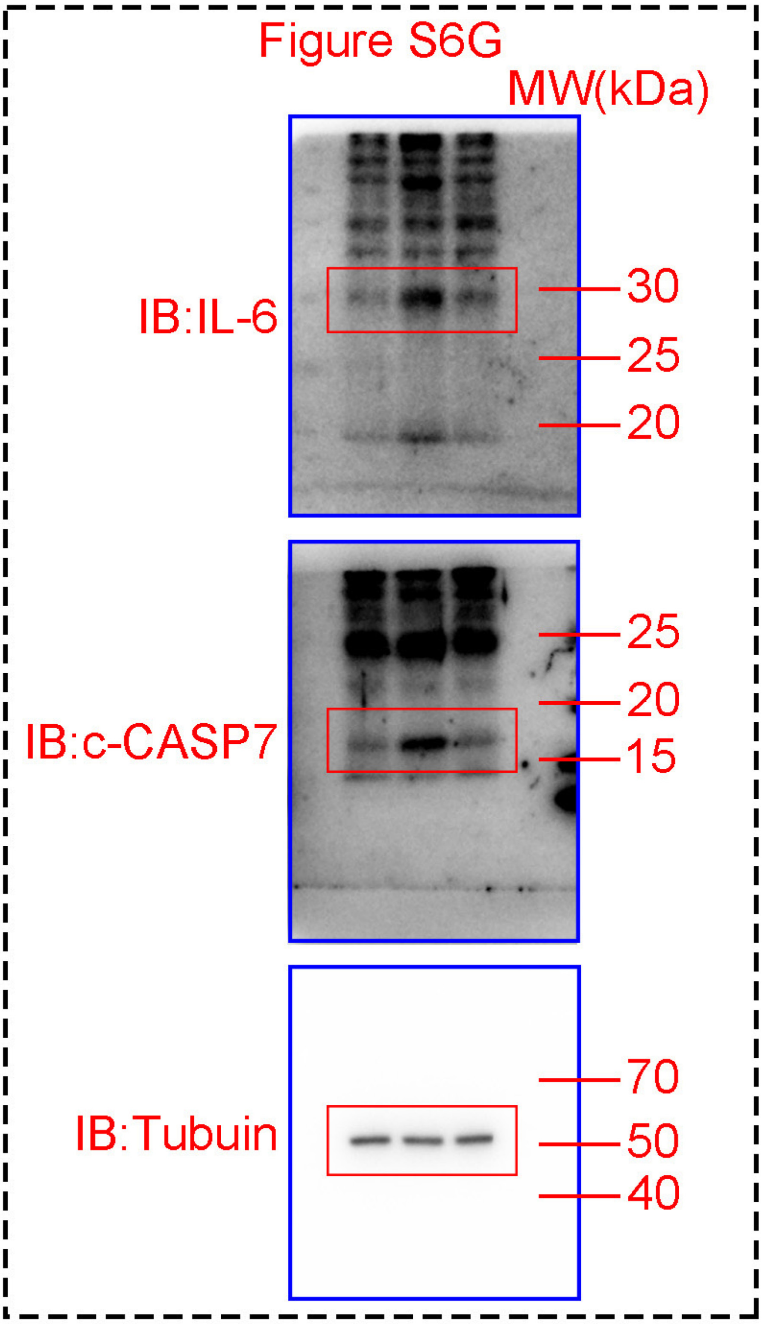
Figure S6G
MW(kDa)
IB:IL-6
30
25
20
IB:c-CASP7
25
20
15
IB:Tubuin
70
50
40

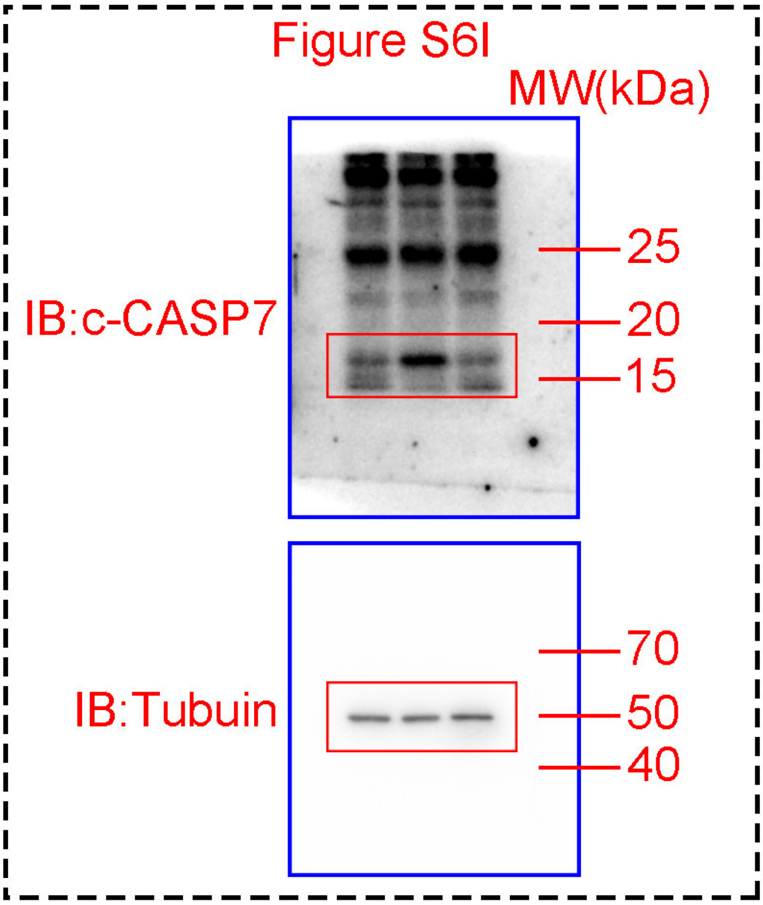
Figure S6I
MW(kDa)
IB:c-CASP7
25
20
15
IB:Tubuin
70
50
40

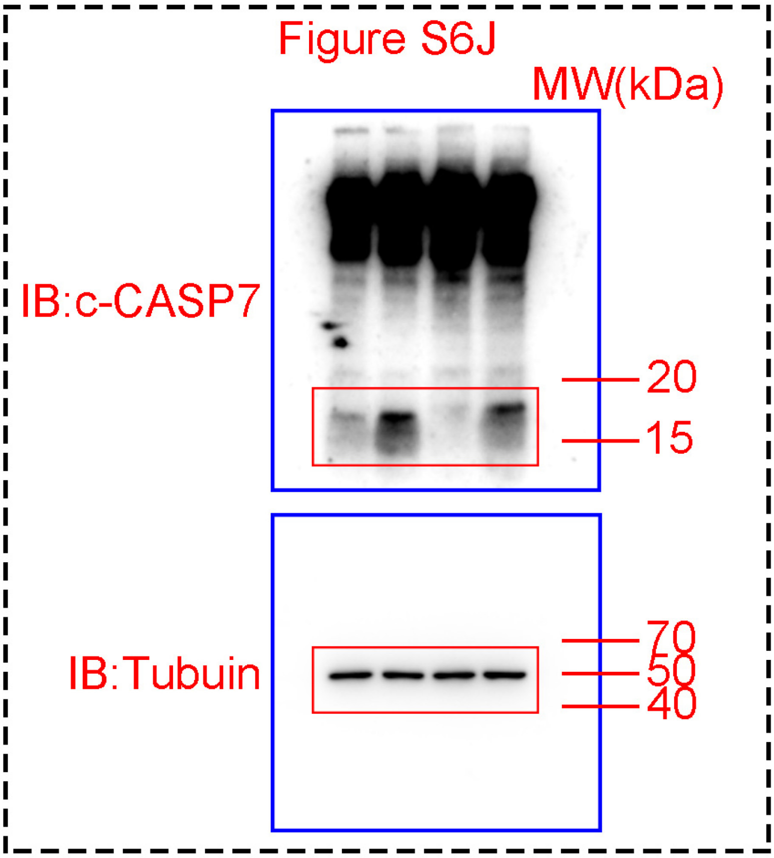
Figure S6J
MW(kDa)
IB:c-CASP7
20
15
IB:Tubuin
70
50
40

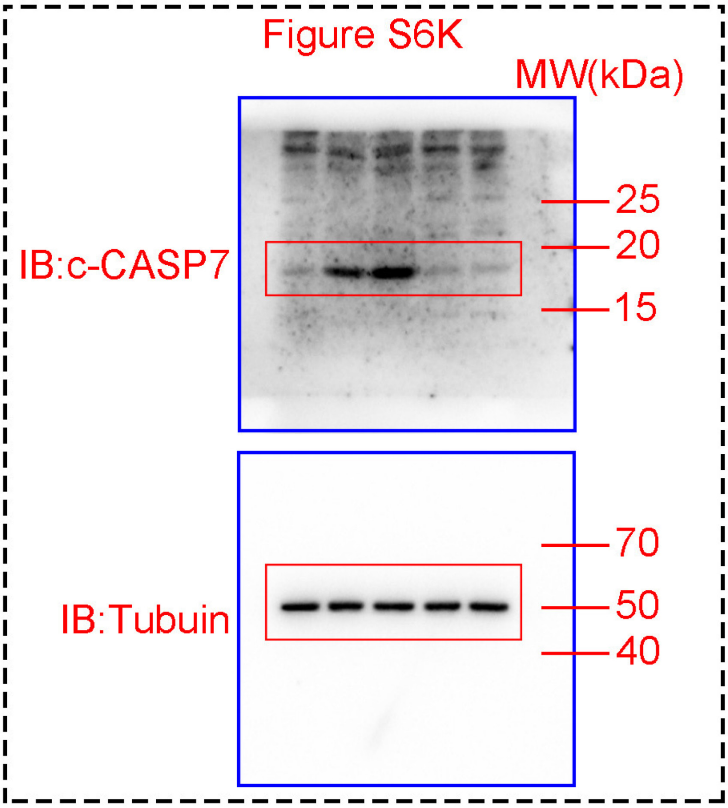

Figure S6K
MW(kDa)
IB:c-CASP7
25
20
15
IB:Tubuin
70
50
40

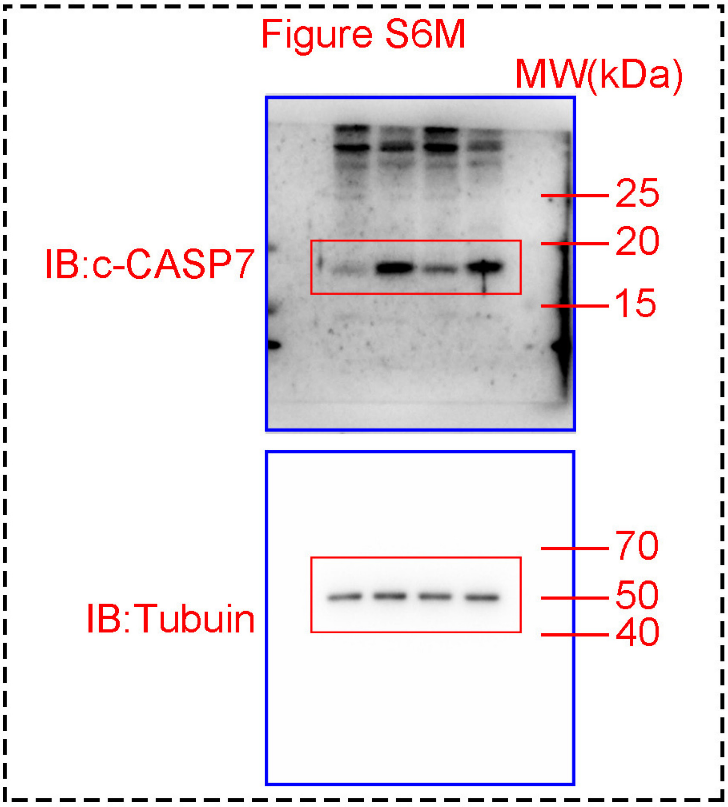

Figure S6M
MW(kDa)
IB:c-CASP7
25
20
15
IB:Tubuin
70
50
40

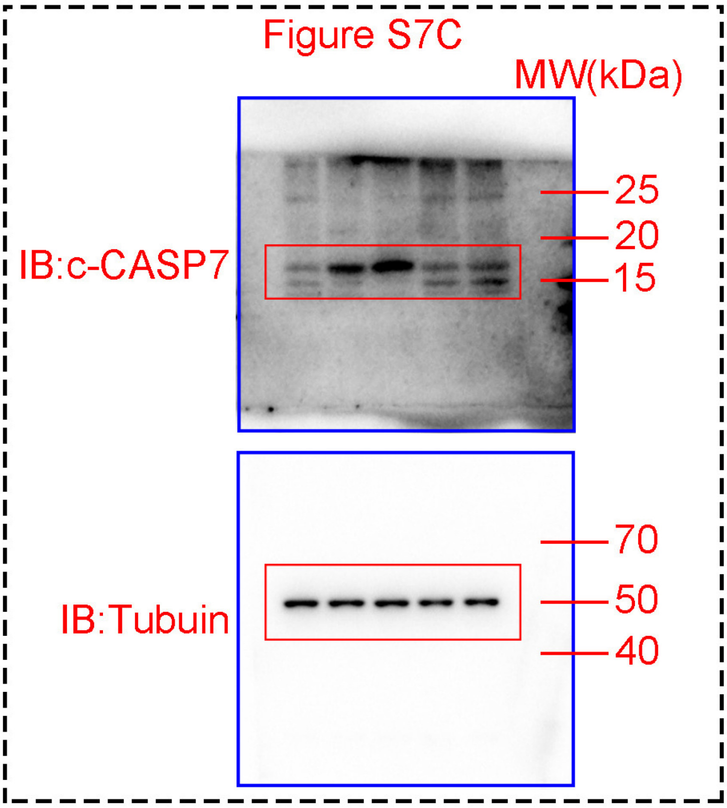

Figure S7C
MW(kDa)
IB:c-CASP7
25
20
15
IB:Tubuin
70
50
40
